# Structure of cytoplasmic RNA polymerase II

**DOI:** 10.1038/s41467-026-75416-8

**Published:** 2026-07-13

**Authors:** Annamaria Hlavata, Benjamin Neuditschko, Ulla Schellhaas, Clemens Plaschka, Franz Herzog, Carrie Bernecky

**Affiliations:** 1 Institute of Science and Technology Austria (ISTA), Klosterneuburg, Austria; 2 Institute Krems Bioanalytics, IMC University of Applied Sciences Krems, Krems, Austria; 3 Research Institute of Molecular Pathology (IMP), Vienna BioCenter (VBC), Vienna, Austria

**Keywords:** Cryoelectron microscopy, Enzymes, Transcription

## Abstract

RNA polymerase II (Pol II) must be assembled in the cytoplasm before it enters the nucleus, where it transcribes protein-coding genes. Although transcription by Pol II is intensively studied, how this central multi-subunit enzyme is made and the role of dedicated assembly factors remains unclear. Here, we report the integrative structural analysis of a native human Pol II from the cytoplasm captured near the end of biogenesis. The complex contains Gdown1 and three biogenesis factors – RPAP2 and the critical small GTPases GPN1 and GPN3. Cryo-EM analysis of the complex reveals how Gdown1 and RPAP2 associate with Pol II and prevent the premature association of transcription factors. Further biochemical and cryo-EM analysis reveals how RPAP2 tethers GPN1–GPN3 to the complex and how the assembly of the RPAP2–GPN1–GPN3 complex is controlled by GTP hydrolysis. The combined results uncover a network of interactions that chaperone cytoplasmic Pol II to prevent aberrant interactions, reveal a molecular switch regulating biogenesis factor association, and suggest a general mechanism for the action of GPN-loop GTPase family of enzymes.

## Abstract (web summary)

Although Pol II transcription is well-studied, its cytoplasmic assembly is poorly understood. Here, the authors reveal the molecular basis for how Pol II is chaperoned before nuclear import, and how GTP hydrolysis controls the interaction of the essential biogenesis factors RPAP2 and GPN1 GPN3.

## Introduction

RNA polymerase II (Pol II) is the eukaryotic enzyme responsible for mRNA synthesis. Although Pol II carries out transcription in the nucleus, it is first assembled in the cytoplasm from 12 distinct subunits before being transported into the nucleus as a fully formed complex^1^. Regulated Pol II assembly and nuclear import are critical for gene expression and its regulation. Pol II biogenesis provides sufficient enzyme to daughter cells after cell division and replenishes Pol II levels after its DNA damage-induced degradation^2^. Furthermore, changes in Pol II levels have been shown to control gene regulation^2–5^. Despite its importance, the mechanisms governing how this essential multi-subunit enzyme is made are poorly understood.

The assembly and import of Pol II utilize several dedicated and general protein factors, which have been identified through Pol II interactome analysis and knockout and depletion experiments^6^. However, the roles of these factors and the mechanisms involved have remained unclear. Pol II degradation followed by mass spectrometry has revealed that partially assembled Pol II complexes associate with chaperones^1^, RNA polymerase-associated proteins (RPAPs)^7^, small GPN-loop GTPases (GPNs)^8,9^, and Gdown1^1^, a factor known to stably associate with 12-subunit Pol II^10^. Thus, these proteins appear to have a role in early stages of Pol II assembly, potentially participating in the merging of Pol II subcomplexes^1^. The assembled cytoplasmic Pol II has also been shown to associate with RPAP2 and GPN1-GPN3^9^, suggesting a potential role of these proteins in late assembly and nuclear import. RPAP2, GPN1-3, and Gdown1 are each essential or important for cell growth and development, as evidenced by CRISPR screens in cancer cell lines^11,12^ and knockout and depletion experiments^7–9,13^.

Functional studies have indicated important roles for RPAP2 and GPN proteins in Pol II assembly and import^7–9^. GPN1 forms a heterodimer with GPN2 or GPN3, with GPN2 implicated in early steps of assembly, whereas GPN3 appears to function later^14,15^. The GPN1-GPN3 complex is the predominant form of GPN1 in mammalian cells^16^, and depletion of either protein or mutation of the GTP-binding pocket of GPN1 reduces nuclear Pol II levels^9^. Similarly, loss of RPAP2 leads to defects in Pol II assembly or nuclear import. GPN1 and the C-terminus of RPAP2 have been reported to interact and interestingly, GPN1 is required for the nuclear export and cytoplasmic recycling of RPAP2^7^.

Gdown1 (also POLR2M, GRINL1A) has been identified as a transcriptional regulator^17–19^, but its role in Pol II assembly remains unclear^1^. Gdown1 contains two nuclear export sequences (NESs) and a cytoplasmic anchoring sequence (CAS)^17^, which keeps Gdown1 predominantly localized to the cytoplasm except during transcriptionally silent developmental stages^13^, stress^17^, and mitosis^18^. Gdown1 becomes phosphorylated during mitosis, particularly at S270^20^, and Gdown1 phosphorylation reduces its binding to Pol II^20^. A plethora of in vitro biochemical data indicates that Gdown1 interferes with all stages of transcription (including initiation, elongation, and termination), but acute Gdown1 depletion has minimal effects on transcription^18^. Altogether, these findings indicate a role for Gdown1 during mitosis and during the cellular stress response, likely helping to shut down transcription. However, the interplay between newly assembled Pol II, biogenesis factors, and Gdown1 remains unclear.

Here, we obtain molecular insights into Pol II assembly and how Pol II is chaperoned in the cytoplasm prior to its import into the nucleus. We apply integrative structural analysis of endogenous and in vitro reconstituted complexes accompanied by biochemical assays, revealing how the essential factors Gdown1, RPAP2, GPN1, GPN3, and a GTP-gated switch may control the final stages of cytoplasmic Pol II biogenesis.

## Results

### Identification of the Gdown1-associated proteome in cytoplasmic and nuclear fractions

**Fig. 1:**
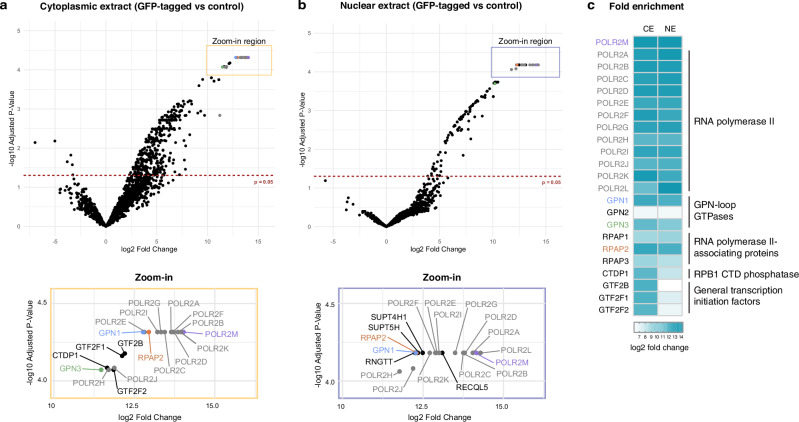
Identification of Gdown1-interacting factors. **a** Volcano plot (full and zoom-in view) of the proteins identified in MS analysis following IP of Gdown1 (gene name POLR2M) from cytoplasmic extract. Red dashed line indicates significant enrichment (*p* < 0.05). Zoom-in view of enriched proteins with *p* < 0.0001. **b** Volcano plot (full and zoom-in view, as in (a)) of the proteins identified in MS analysis following IP of Gdown1 from nucleoplasmic extract. Statistical significance of enriched proteins in (**a**, **b**) was calculated using limma^56^ (two-sided significance test, *P*-values adjusted for multiple testing). **c** Heatmap comparing the abundance of top cytoplasmic Gdown1-associating proteins, RPAP proteins, and GPN proteins recovered from the cytoplasmic and nuclear fractions, with darker blue color representing increasing fold change. Source data are provided as a Source data file.

To investigate late events in human Pol II biogenesis, we purified cytoplasmic Pol II complexes from proliferating cells. To avoid perturbation of any Pol II surface, we utilized Gdown1 as a purification handle, given the high stability of the Gdown1-Pol II interaction^20^, Gdown1’s predominantly cytoplasmic localization^13,17,18^, and the significant fraction (30–50%) of Pol II-Gdown1 complexes in whole-cell extracts^10^. We generated a K562 cell line in which both endogenous copies of Gdown1 were tagged with eGFP followed by a 3C protease cleavage site, to allow affinity purification of Gdown1-associated complexes and their elution from the resin under mild conditions (Supplementary Fig. 1a–c). We then characterized the Gdown1 interactome in both the cytoplasm and nucleoplasm using immunoprecipitation and mass spectrometry analysis, observing many significantly enriched proteins (*p* < 0.05; Fig. 1a, b, Supplementary Fig. 1d–f). All subunits of Pol II (RPB1-12) were significantly enriched, consistent with the predominant accumulation of assembled Pol II at steady state. We also identified many proteins previously shown to interact with Pol II, including RPAP1-3, GPN1-3, DSIF (SUPT5H/SUPT4H), RECQL5, TFIIS (TCEA1), and the Mediator and Integrator complexes^1,8,21,22^ (Supplementary Fig. 1e, f).

Comparison of the most significantly enriched cytoplasmic and nucleoplasmic Gdown1 interactors revealed high enrichment of RPAP2, GPN1, and GPN3 in both cytoplasm and nucleoplasm (Fig. 1c). Given the enrichment of known Pol II biogenesis factors, a significant fraction of the nuclear Pol II-Gdown1 complex may represent Pol II that was newly imported into the nucleus, but which has not yet engaged in transcription, and may suggest that Gdown1, RPAP2, GPN1 and GPN3 are imported together to the nucleus from the cytoplasm. Although no structural information regarding GPN1 and GPN3 interaction with Pol II is available, structures of Pol II-RPAP2 have revealed how the N-terminal Pol II-interacting domain of RPAP2 binds to the Pol II cleft and blocks nucleic acid association^23,24^. Altogether, the proteomic analysis suggested that a Pol II complex enriched in Gdown1, RPAP2, GPN1, and GPN3 could be isolated from the cytoplasm.

### Architecture of endogenous cytoplasmic RNA polymerase II

**Fig. 2:**
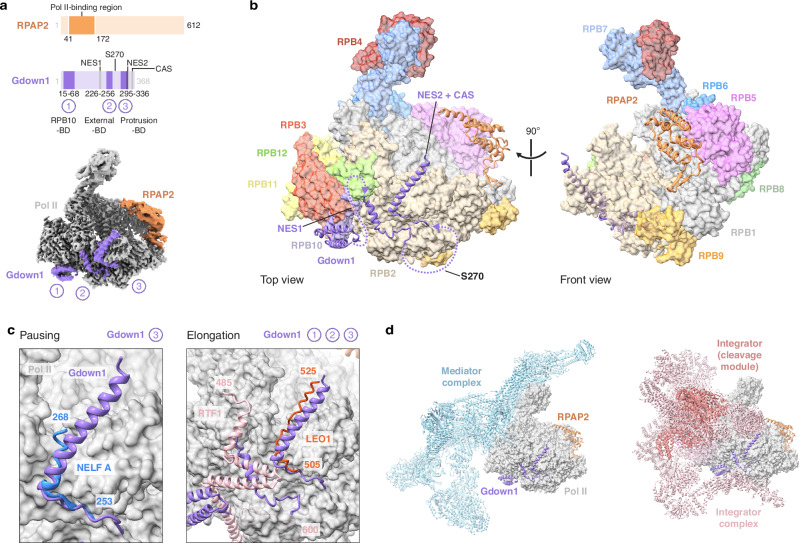
Structural analysis of cytoplasmic Pol II. **a** Top, schematic of RPAP2 and Gdown1, with regions resolved in the cytoplasmic Pol II structure shown in dark orange and purple, respectively. The Gdown1 RPB10-binding domain (interface 1), external-binding domain (interface 2), and protrusion-binding domain (interface 3) are indicated. Bottom, top view of the composite cytoplasmic Pol II reconstruction. Pol II, gray; RPAP2, orange, Gdown1, purple. **b** Top and front view of the Pol II-Gdown1-RPAP2 structure, with Pol II colored by subunit. **c** Superposition of the Pol II-Gdown1-RPAP2 structure with a structure of paused Pol II bound to DSIF and NELF (left, PDB ID 8UHD^76^) and a structure of an activated Pol II PAF1C and RTF1-containing elongation complex (right, PDB ID 6TED^77^). The Gdown1 regions involved in clashes are noted with circled numbers. Colored as follows: NELF A, blue; LEO1, red; RTF1, light pink. **d** Comparison of the Pol II binding interfaces of the Mediator (left, PDB ID 7ENA^32^) and Integrator (right, PDB 8RBX^29^) complexes with those of Gdown1 and RPAP2. Colored as follows: Mediator, light blue; Integrator, pink; Integrator cleavage module, dark pink.

To gain insight into the architecture of the cytoplasmic Pol II complex prior to nuclear import, we aimed to determine its structure. Gdown1-containing complexes were purified from the cytoplasm of K562 cells endogenously expressing GFP-tagged Gdown1, followed by GraFix separation to enrich for Pol II-containing complexes. Cryo-EM analysis of the resulting complexes revealed the structure of Pol II bound to Gdown1 and RPAP2 at a nominal resolution of 2.7 Å. In the reconstruction, 12-subunit Pol II, the Pol II-binding region of RPAP2, and three distinct Pol II-binding regions of Gdown1, but no GPN1-GPN3 density, could be resolved (Fig. 2a, b and Supplementary Fig. 2). As expected for an RPAP2-bound Pol II complex, no nucleic acid density was visible. RPAP2 was bound to the RPB1 jaw and RPB5, essentially as previously observed^23,24^, and the Pol II clamp and active site were not resolved, even after extensive 3D classification. Thus, the Pol II clamp domain appears highly flexible, consistent with previous studies of nucleic acid-free Pol II complexes^13,23–25^. Due to improved density, a previously existing model for Pol II-Gdown1^13^ could be revised. In the updated model, the N-terminal Pol II-binding regions of Gdown1 were revealed to be discontinuous, and the sequence register of both regions of Gdown1 could be assigned. Furthermore, the model for the Gdown1 region binding to the RPB2 protrusion could be extended (Supplementary Fig. 2k). Altogether, a more extensive binding interface with RPB2 could be observed and three Gdown1-Pol II interfaces could be identified, including an RPB10-binding domain (interface 1), an external-binding domain (interface 2), and a protrusion-binding domain (interface 3). Two NESs and one CAS are localized in unstructured regions, and thus likely to be functional in the context of the Pol II-Gdown1 complex. Phosphorylation of Gdown1 at serine 270 has been shown to occur during mitosis and reduce the affinity of Gdown1 for Pol II early elongation complexes^20^. This residue of Gdown1 is located in a disordered region, suggesting that it is available for phosphorylation in the context of the Pol II-Gdown1 complex, and that additional protein or nucleic acid factors may be required to decrease affinity to Pol II. To validate our model, we performed chemical crosslinking combined with mass spectrometry (XL-MS) of the cytoplasmic sample. Although few inter-subunit crosslinks were observed, all were consistent with the Pol II-Gdown1-RPAP2 structure and previous XL-MS analysis of a Pol II-Gdown1 binary complex^13^, suggesting that the Pol II-Gdown1 interface is the same in the presence of additional factors present in the cytoplasm (Supplementary Fig. 3a–c).

Gdown1 has been shown in vitro to inhibit all stages of transcription, including initiation^10^, promoter-proximal pausing^26^, elongation^18^, and termination^20^. Structural and biochemical competition between Gdown1 and TFIIF has been well characterized, and as expected, we observed a structural clash between the Gdown1 protrusion-binding domain and the small subunit of TFIIF, as well as a small clash with TFIIB (Supplementary Fig. 3d). This structural incompatibility, together with biochemical disruption of the Pol II-TFIIF interaction by RPAP2^24^, suggests that the cytoplasmic enrichment of TFIIF (GTF2F1, GTF2F2) in the cytoplasmic Gdown1 IP-MS is due to an alternative protein-protein interaction (Fig. 1a and Supplementary Note 1). Comparison of the Pol II-Gdown1-RPAP2 structure to an AlphaFold3-predicted structure of Pol II and the mitotic transcription termination factor TTF2 revealed that the Gdown1 RPB10-binding domain is responsible for the observed biochemical competition between the factors (Supplementary Fig. 3d, e). Gdown1 has also been shown to relieve repression by the pausing factor NELF^26^. This effect can be explained by a structural clash between the Gdown1 protrusion-binding domain and the NELF A subunit of the NELF complex (Fig. 2c). Furthermore, previously observed functional competition between Gdown1 and the PAF1 complex (PAF1C) with RTF1^18^ can be explained by a clash between the PAF1C subunit LEO1 and the protrusion-binding domain of Gdown1 and between RTF1 and the RPB10- and external-binding domains of Gdown1 (Fig. 2c).

Notably, Gdown1 does not appear to interfere with the binding of many essential transcription factors. For example, comparison to a structure of Mediator within a TFIID-containing pre-initiation complex revealed no direct competition between Mediator and Gdown1 (Fig. 2d). Thus, the previously reported activity of the Mediator complex in relieving Gdown1-based transcriptional repression^10^ is likely due to Mediator-dependent stimulation of pre-initiation complex assembly, for example through stabilization of TFIIB binding^27^, allowing general transcription initiation factors to displace Gdown1 binding to their interfaces. Structural compatibility is also observed between Gdown1 and the transcription elongation factor DSIF, a platform for the binding of many transcriptional cofactors and which associates with Pol II shortly after promoter escape until transcription termination^28^, consistent with previous biochemical data^26^ (Supplementary Fig. 3f). Furthermore, Gdown1 binding is also compatible with association of the Integrator complex, despite the extensive binding interfaces between Integrator and Pol II^29,30^ (Fig. 2d, Supplementary Fig. 3g). Integrator is a large multi-subunit complex with RNA cleavage activity and key roles in termination of noncoding RNAs and promoter-proximally paused Pol II^31^. Thus, Gdown1 would be located in a way consistent with displacement of the pausing factor NELF without impeding the pausing termination activity of Integrator. Inspection of enriched proteins in the Gdown1 IP-MS experiments revealed that Mediator, DSIF, and the Integrator complex were all enriched, particularly in the pulldown from nucleoplasmic extracts, consistent with the interactions of Mediator and Integrator with the CTD of the largest subunit of Pol II^32,33^ and the structural compatibility between Gdown1 and these complexes when bound to Pol II (Supplementary Fig. 1e, f).

### Analysis of a reconstituted RNA polymerase II-Gdown1-RPAP2-GPN1-GPN3 complex

**Fig. 3:**
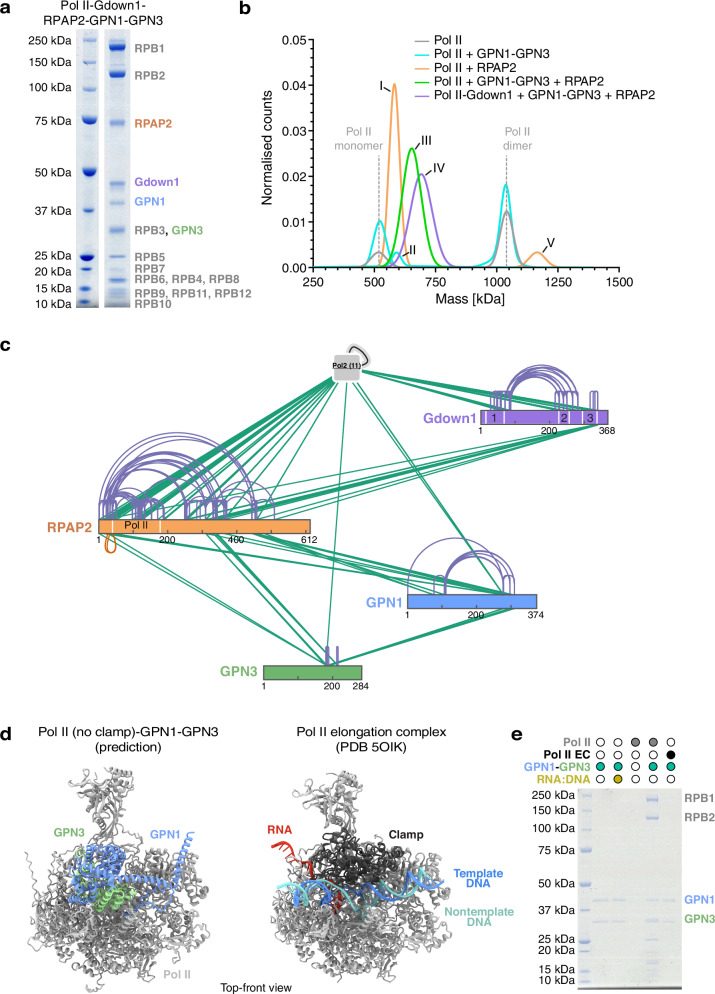
Architecture of the Pol II-Gdown1-RPAP2-GPN1-GPN3 complex. **a** SDS-PAGE analysis (Coomassie) of size-exclusion-purified recombinant Pol II-Gdown1-RPAP2-GPN1-GPN3. The experiment was repeated three times, with similar results. **b** Mass photometry analysis (fitted mass distributions) of Pol II alone or in the presence of RPAP2, GPN1-GPN3, and/or Gdown1. Molecular weights corresponding to the unbound Pol II monomer and dimer are indicated with dashed gray lines. The molecular weights of peaks I-V were consistent with those of the following species: I – Pol II-RPAP2; II – Pol II-GPN1-GPN3; III – Pol II-RPAP2-GPN1-GPN3; IV – Pol II-Gdown1-RPAP2-GPN1-GPN3; V – Pol II-RPAP2 dimer. **c** Crosslinking-mass spectrometry of recombinant Pol II-Gdown1-RPAP2-GPN1-GPN3. Violet, self crosslinked peptides; green, heteromeric crosslinked peptides. **d** Top-front views of the Pol II (no clamp)-GPN1-GPN3 structure prediction and the Pol II elongation complex (Pol II EC, PDB ID 5OIK^78^). Colored as follows: Pol II, gray; GPN1, blue; GPN3, green; Pol II EC clamp, black; RNA, red; template DNA, blue; non-template DNA, cyan. **e** Pull-down of Pol II and Pol II elongation complex (Pol II EC) via immobilized His-GPN1-GPN3. The experiment was repeated three times, with similar results. Source data are provided as a Source data file.

Analysis of the proteins that co-migrated with Pol II in sucrose gradient centrifugation (Supplementary Fig. 4a), as well as a body of previously published work^9,21^ suggests that Pol II, RPAP2, Gdown1, and GPN1-3 exist as part of a single complex in the cytoplasm. Additionally, previous work has indicated a direct interaction between GPN1 and both Pol II and RPAP2^7^. However, cryo-EM and XL-MS analysis of the endogenous cytoplasmic Pol II complex did not reveal the location of GPN1 and GPN3, despite their relatively high enrichment in IP-MS in both cytoplasmic and nuclear Gdown1 complexes. To investigate the interactions of GPN1-GPN3 within the cytoplasmic Pol II complex, we reconstituted a complex containing Pol II, Gdown1, RPAP2, and GPN1-GPN3 from purified components (Supplementary Fig. 4b, c). We assessed complex formation by mass photometry and size-exclusion chromatography, both of which indicated assembly of the Pol II–Gdown1–RPAP2–GPN1–GPN3 complex (Fig. 3a, b, Supplementary Fig. 4d, and Supplementary Note 2). Mass photometry was applied to independently assess the interactions of Pol II with RPAP2 and GPN1-GPN3. Isolated Pol II was primarily dimeric, in line with previous work showing that purified Pol II forms a dimeric complex, which has been suggested to be a storage form of Pol II^34,35^. Recombinant RPAP2 readily bound the Pol II complex, incorporating into both Pol II monomeric and dimeric forms, but decreasing the fraction of Pol II dimers. Separately, recombinant GPN1-GPN3 shifted a small fraction of monomeric Pol II, but did not incorporate into the dimer, suggesting a direct but relatively weak Pol II-GPN interaction. When added together, the presence of Pol II dimer was strongly reduced, and Pol II was quantitatively shifted into a monomeric Pol II-RPAP2-GPN1-GPN3 complex, indicating that RPAP2 is required to assemble a stable complex with GPN1-GPN3. Gdown1, which has been previously shown to reduce Pol II dimer formation on its own^35^, readily incorporated into this monomeric Pol II-RPAP2-GPN1-GPN3 complex (Fig. 3b). Taken together, these observations strongly suggest that cytoplasmic Pol II exists predominantly in a monomeric form in proliferating cells, consistent with the lack of observed dimers in cryo-EM of the native, cytoplasmic Pol II.

To gain further insights into the architecture of the complex, we identified chemical crosslinks of the reconstituted Pol II-Gdown1-RPAP2-GPN1-GPN3 by mass spectrometry. The obtained crosslinks were consistent with the cytoplasmic Pol II structure (Fig. 3c and Supplementary Fig. 4e, f). We observed a large number of inter-protein crosslinks, including multiple links between the unstructured N-terminal region of RPAP2 and Pol II, which were consistent with a localization near the Pol II clamp and DNA-binding cleft. This region also crosslinked to the Gdown1 protrusion-binding domain (interface 3), consistent with a localization near the Pol II DNA-binding cleft, and to GPN1 and GPN3. We observed multiple crosslinks between GPN1-GPN3 and the C-terminus of RPAP2, consistent with previous observations that the C-terminus of RPAP2 is required for its association with GPN1^7^. Furthermore, GPN1-GPN3 crosslinked to RPB1, the largest subunit of Pol II, through its clamp and C-terminal domains (Supplementary Fig. 4g).

To explore the direct interaction between GPN1-GPN3 and Pol II, we performed structure prediction of a Pol II-GPN1-GPN3 complex using AlphaFold3^36^. Clashes predicted between Pol II and GPN1-GPN3 suggested that an open clamp conformation would be required for binding of GPN1-GPN3 to Pol II (Supplementary Fig. 4h, i). Structure prediction of Pol II-GPN1-GPN3 lacking the clamp domain, which was unresolved and presumably mobile in our experimental Pol II-Gdown1-RPAP2 structure, yielded a higher-confidence model without clashes (Supplementary Fig. 4j, k). Analysis of this model suggested that GPN1-GPN3 interacts with the DNA-binding cleft of Pol II through electrostatic interactions mediated by negatively charged regions of GPN1 and GPN3. Extensive interactions were observed between the negatively charged GPN3 C-terminal tail and the Pol II pore and funnel domains, which accommodate backtracked RNA in the arrested elongation complex^37^ (Fig. 3d and Supplementary Fig. 4l). The predicted GPN1-GPN3 and Pol II interaction would be blocked in a Pol II elongation complex, through steric clashes with bound nucleic acid and clamp closure. To test this, we incubated GPN1–GPN3 with apo or nucleic acid-bound forms of Pol II in a pulldown binding assay (Fig. 3e). Whereas GPN1–GPN3 was able to associate with Pol II, it did not interact with the Pol II elongation complex, suggesting that its primary binding site indeed lies within the DNA-binding cleft rather than the stalk (RPB4/7) or the C-terminal domain (CTD) as previously suggested^9^. Altogether, these results suggest that GPN1-GPN3 interacts directly and likely transiently with the Pol II DNA-binding cleft and is anchored to the Pol II complex through the flexibly associated, unresolved C-terminal region of RPAP2.

### Structural and biochemical analysis of RPAP2-GPN1-GPN3

**Fig. 4:**
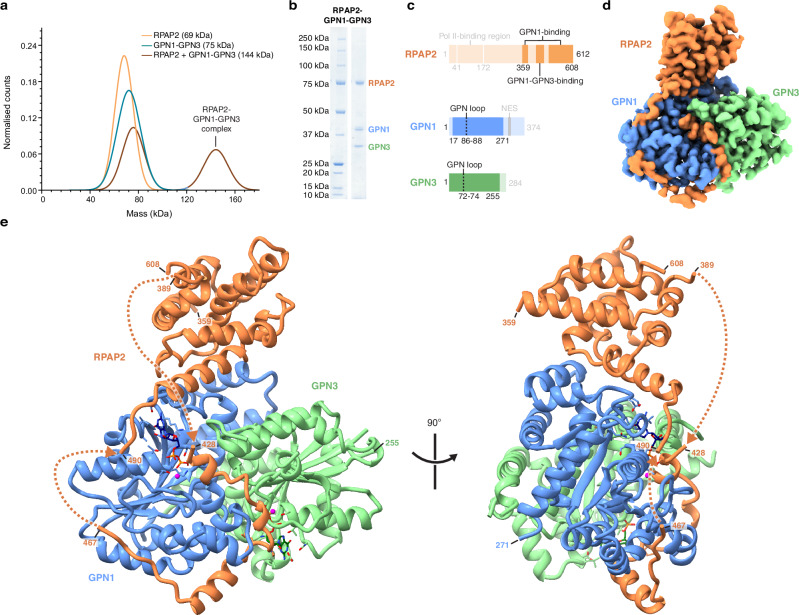
Structural analysis of RPAP2-GPN1-GPN3. **a** Mass photometry analysis (fitted mass distributions) of RPAP2, GPN1-GPN3, and RPAP2-GPN1-GPN3. **b** SDS-PAGE analysis (Coomassie) of size-exclusion-purified RPAP2-GPN1-GPN3, performed in triplicate with similar results. **c** Schematic of RPAP2, GPN1, and GPN3 proteins, in which unresolved regions of the RPAP2-GPN1-GPN3 structure of at least 10 amino acids in length are shown in faded colors. RPAP2, orange; GPN1, blue; GPN3, green. **d** Cryo-EM density map of RPAP2-GPN1-GPN3 colored as in (**c**). **e** Two views of the RPAP2-GPN1-GPN3 structure, with the connectivity of the unresolved regions of RPAP2 indicated with orange dashed lines. Source data are provided as a Source data file.

To visualize how GPN1-GPN3 is anchored to the cytoplasmic Pol II complex through RPAP2, we reconstituted an RPAP2-GPN1-GPN3 complex. This complex was readily observed in mass photometry analysis and size exclusion chromatography (Fig. 4a, b). Pulldown experiments utilizing purified proteins showed that the Pol II-binding region of RPAP2 is not able to directly interact with GPN1-GPN3 (Supplementary Fig. 5a), suggesting that the observed crosslinks between the RPAP2 N-terminus and GPN1-GPN3 were due to proximity in the context of the Pol II-Gdown1-RPAP2-GPN1-GPN3 complex, rather than a direct interaction between the two. Cryo-EM analysis of the purified complex resulted in a 2.8-Å nominal resolution structure, into which the GPN1-GPN3 heterodimer and a C-terminal region of RPAP2 could be modeled (Fig. 4c–e and Supplementary Fig. 5b–h). The majority of GPN1 and GPN3 were well-resolved, except for the very C-terminal ends of both, which were flexible and solvent-exposed. This flexible region includes the GPN1 NES. RPAP2 formed an extended interface with the GPN1-GPN3 dimer. A largely alpha-helical, GPN1-binding domain of RPAP2 was formed by residues 359–389 and 490–608, associating with GPN1 in close proximity to the GTP/GDP binding pocket. A second region of RPAP2 encompassing residues 428–467 bound to the GPN1-GPN3 dimerization interface, continuing along GPN1. A hydrophobic patch previously suggested to associate with disordered hydrophobic peptides^38^ was buried within the GPN1-GPN3 complex (Supplementary Fig. 5i). However, several surface-exposed hydrophobic residues are located at the interface between GPN1 and GPN3, suggesting that other binding pockets may allow allosteric activation of GPN1 GTPase activity as previously described^38^ (Supplementary Fig. 5j). Mapping the crosslinks of the Pol II-RPAP2-GPN1-GPN3 complex onto the structure RPAP2-GPN1-GPN3 showed overall good agreement with the experimentally determined model compared with an AlphaFold3-predicted model (Supplementary Fig. 5k–n), validating the interaction of RPAP2 with GPN1 in proximity to the GPN3 nucleotide-binding pocket.

**Fig. 5:**
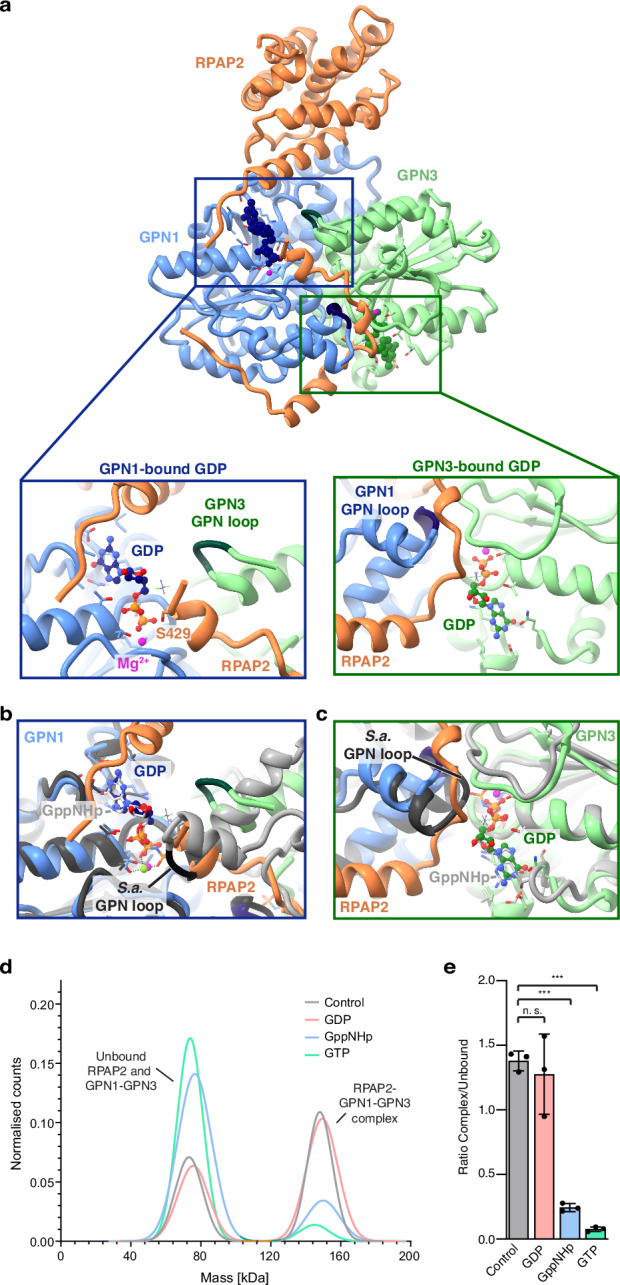
Biochemical and structural analysis of GPN1-GPN3 bound to RPAP2. **a** A view of the GPN1 and GPN3 nucleotide-binding sites with bound GDP. Left, overview image; right, zoom-in views of the GPN1 (top) and GPN3 (bottom) binding pockets. GPN3 GPN loop, dark green; GPN1 GPN loop, dark blue; Mg^2+^ ions, magenta. **b**, **c** Superposition of the human RPAP2-GPN1-GPN3 structure (this study) with that of the archeal SaGPN dimer bound to the non-hydrolyzable GTP analog GppNHp (PDB ID 7ZHF^39^. **b** View of GPN1-bound GDP. Colored as follows: SaGPN monomer 1, dark gray; SaGPN monomer 2, gray; SaGPN-bound GppNHp, light gray, SaGPN-loop black; RPAP2-GPN1-GPN3 structure as in (**a**). **c** View of GPN3-bound GDP. Colored as in (**b**). **d** Mass-photometry (fitted mass distributions) of RPAP2-GPN1-GPN3 complex formation after GPN1-GPN3 pre-treatment with GTP, GppNHp, or GDP. **e** Ratio of the counts of RPAP2-GPN1-GPN3 complex versus unbound proteins as in (**d**), performed in biological triplicate. Data are presented as mean values ± SD. Significance level relative to the control was calculated using an unpaired, two-tailed t-test. n.s not significant, *** *p* < 0.0001. Source data are provided as a Source data file.

Inspection of the density within the nucleotide binding pockets of GPN1 and GPN3 revealed that both were bound to a molecule of GDP, which had been co-purified with the complex after recombinant expression in insect cells (Supplementary Fig. 5o). This observation coupled to the conservation of the GTPase G1-G3 and GPN motifs^38^ indicates that in the context of a GPN1-GPN3 heterodimer, both GPN1 and GPN3 are capable of GTP hydrolysis. In the presence of GTP or its non-hydrolyzable analog, the GPN loop is involved in the coordination of the γ-phosphate, leading to a more closed dimeric arrangement^39^. In the RPAP2-GPN1-GPN3 structure, the GPN-loops of both GPN1 and GPN3 were positioned away from the bound nucleotide, consistent with a GDP-bound state (Supplementary Fig. 5p). RPAP2 passed directly next to the GDP molecules of both GPN1 and GPN3, with a mode of interaction incompatible with the closed, GTP-bound state (Fig. 5a-c). This observation is in agreement with previous work suggesting that GDP may increase the RPAP2-GPN1 binary interaction^7^, and suggested that RPAP2 preferentially recognizes the GDP-bound conformation of the GPN1-GPN3 complex. To test this, we performed mass photometry measurements of GPN1-GPN3 and RPAP2 complexes formed by incubation of RPAP2 with GPN1-GPN3 bound to copurified GDP. Complex formation was additionally assessed after incubation with an excess of GDP, GTP, or the non-hydrolyzable GTP analog GppNHp. The RPAP2-GPN1-GPN3 complex readily formed without additional nucleotide or in the presence of excess GDP. However, RPAP2 binding was inhibited in the presence of GppNHp, and even further reduced in the presence of GTP (Fig. 5d, e). Complementary pulldown experiments qualitatively confirmed the reduction in RPAP2 affinity for GPN1-GPN3 in the presence of GTP (Supplementary Fig. 5q). Together, these results identify RPAP2 as a sensor of GPN1-GPN3 state and allow modeling of a cytoplasmic Pol II structure in which GPN1-GPN3 are anchored to the complex through RPAP2.

## Discussion

The structural and biochemical analysis of cytoplasmic Pol II provides insights into its biogenesis and the functions of key interactors, with implications for Pol II regulation in the nucleus and cytoplasm.

### Gdown1-dependent inhibition of Pol II-factor binding

In the revised Gdown1 structure presented here, three distinct Pol II-binding regions could be discerned and their sequence register assigned. These updates allowed clarification of the interactions responsible for the transcription inhibition activity of Gdown1, a phenomenon first recognized 20 years ago^10^. One intriguing prior observation is that Gdown1 inhibits pausing in vitro^26^, which we could explain as an effect of competition with the pausing factor subunit NELF A. At the same time, Gdown1 has been described to localize together with paused Pol II in ChIP experiments^26^. One intriguing possibility is that Gdown1 may be able to incorporate into a stalled Pol II complex on the way to its termination, for example through the action of Integrator.

The association of Gdown1 with cytoplasmic Pol II complexes together with its role in mitosis and response to stress suggests a possibility for crosstalk and coordination between Pol II assembly and cell growth. For example, Gdown1 may help enforce a requirement for Mediator as transcription is re-started following DNA replication and localization of factors is being re-established. Further work is required to elucidate the role of Gdown1 in the nucleoplasm, especially its role during mitosis and stress response.

### Pol II-Gdown1 interactions in the context of Pol II biogenesis

Gdown1 directly contacts the majority of the Pol II subunits thought to belong to the RPB2 subcomplex assembly intermediate, including RPB2, RPB3, RPB10, and RPB12^1^. Previous mass spectrometry work has suggested that a RPB1 subcomplex also associates with Gdown1 and with the R2TP/Prefoldin-like cochaperone complex, which contains many proteins including Unconventional prefoldin RPB5 interactor 1 (URI1)^1^. Structural prediction suggests that Gdown1 and URI1 directly interact in a way that would conflict with the interaction between Gdown1 and the RPB2 protrusion domain (interface 3)^40^. Speculatively, after the RPB1 and RPB2 subcomplexes are merged, Gdown1 may release URI1 in favor of the three interfaces described in this study. Further work, for example Gdown1 interactome analysis under conditions in which Pol II assembly is stimulated, will be required to investigate a role of Gdown1 in Pol II assembly.

### Interaction of Pol II with factors required for assembly and nuclear import

Although GTP binding was required for yeast GPN1 to immunoprecipitate Pol II^41^, this is not the case for mammalian Pol II, as we observed binding between immobilized GPN1-GPN3 (purified in a GDP-bound state) and Pol II in the absence of added nucleotide. This may be due to the flexible clamp domain of human Pol II, which allows GPN1-GPN3 to associate with the DNA/RNA binding cleft even after RPB1 has joined the assembly. Conversely, in yeast, assembly of the RPB1 clamp might block GPN1-3 binding to the same region. Thus, GTP binding by GPN1 may facilitate Pol II interaction indirectly, through a distinct GPN1 and Pol II binding partner. The human Pol II-GPN1-GPN3 structure prediction suggests that GPN1–GPN3 binding would sterically prevent DNA engagement and clamp closure, similar to the reported ability of RPAP2 to compete with downstream DNA^23,24^. Thus, the DNA binding cleft of Pol II would be chaperoned through protein-protein interactions, which may reduce spurious contacts with nucleic acids in the cytoplasm.

To visualize a potential Pol II and GPN1-GPN3 interaction in the context of cytoplasmic Pol II, we prepared a putative model of the Pol II-Gdown1-RPAP2-GPN1-GPN3 complex. We combined the Pol II-Gdown1-RPAP2 and RPAP2-GPN1-GPN3 complex structures elucidated in this work with the relative orientations of Pol II and GPN1-GPN3 from structure prediction(Supplementary Fig. 6). The resulting model would suggest that GPN1-GPN3 could associate with the Pol II DNA binding cleft without disruption of the Pol II-Gdown1-RPAP2 complex structure. Mapping the Pol II-Gdown1-RPAP2-GPN1-GPN3 complex crosslinks onto this model is compatible with the existence of this state, although the interaction may be transient. In a recent preprint, released as this work was under review^42^, Schmitzová and colleagues performed crosslinking mass spectrometry analysis of cytoplasmic Pol II complexes isolated through an affinity tag on the RPB1 subunit. Their analysis revealed multiple crosslinks between GPN3 and RPB2, also supporting a localization of GPN1-GPN3 to the DNA binding cleft. Furthermore, their work included cryo-EM analysis of a cytoplasmic Pol II complex supplemented with recombinant Gdown1 and RPAP2-GPN1-GPN3 complex to improve stoichiometry. The structure reported in Schmitzová et al. is highly similar to the structure of cytoplasmic Pol II reported here, suggesting that flexibility of bound GPN1-GPN3, rather than substoichiometry in the endogenous complex, prevents visualization of GPN1-GPN3 in the cytoplasmic Pol II complex.

### Function and mechanism of GPN-loop GTPases

GPN1, GPN2, and GPN3 are small eukaryotic GTPases that are required for Pol II assembly and import^8,9^. They belong to a class of proteins known as GPN-loop GTPases, named after a glycine-proline-asparagine (GPN) motif required for their catalytic activity^43^. The GPN-loop GTPase family is conserved from archaea to eukaryotes^9,38,39^ and these enzymes function as constitutive homodimers or heterodimers, independent of GTP- or GDP-bound state^16^. Although homodimers have been observed for recombinantly expressed, purified yeast^38^ and human^44^ GPN1, in a cellular context, eukaryotic GPN1 forms heterodimers with either GPN2 or GPN3^45^. In eukaryotes, the majority of GPN1 exists within a heterodimeric complex with GPN3^16^. Thus, the study of the heterodimeric state is necessary to understand eukaryotic GPN-loop GTPase structure and function.

Despite X-ray crystallographic studies of two archaeal GPN-loop GTPases^39,43^ and isolated yeast Gpn1^38^, how GPN-loop GTPases fulfill their function and the role of GTP hydrolysis in this process remains enigmatic. Analysis of the GTP- and GDP-bound states of the GPN enzyme from the archaea *Sulfolobus acidocaldarius* showed that GTP hydrolysis leads to an allosteric change in the dimerization interface, converting between a closed (GTP-bound) conformation and an open (GDP-bound) conformation^39^. Despite this insight, how this subtle conformational change links to GPN function remained unclear. Furthermore, previous structural and biochemical analysis of isolated yeast Gpn1 (Npa3) revealed that the presence of hydrophobic peptides could stimulate the GTP hydrolysis activity of Gpn1^38^. In this work, the structure of GDP-bound GPN1-GPN3 bound to RPAP2 reveals an extended interaction with GPN1 and the GPN1-GPN3 dimerization interface, with the interface interaction requiring GPN1-GPN3 to be in a GDP-bound conformation. The biochemical results presented here suggest that GTP binding, and perhaps more potently the conformational changes undergone during GTP hydrolysis, destabilize RPAP2 binding. Thus, RPAP2 is the first known effector of GPN-loop GTPases, suggesting a general mechanism by which the GPN dimerization interface, specifically the regions adjacent to the nucleotide-binding pockets, serves as a regulated binding site for effectors.

### Putative expanded model of Pol II biogenesis

**Fig. 6:**
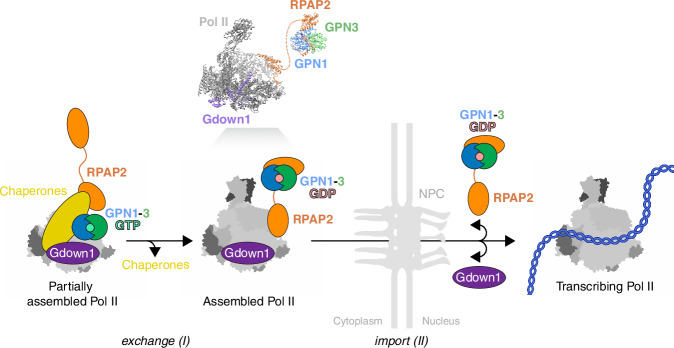
Schematic depicting an expanded model of Pol II biogenesis and biogenesis factor recycling. Gdown1, RPAP2, GPN1-GPN3, and chaperones associate with partially assembled Pol II, assisting in early stages of Pol II assembly and in the joining of Pol II subcomplexes. In the context of partially assembled Pol II, hydrophobic peptides or chaperone-associated factors would act to keep GPN1-GPN3 in a predominantly GTP-bound state. After the completion of Pol II assembly, GPN1-GPN3 adopts a GDP-bound state and remains stably bound to the C-terminus of RPAP2. After import of the Pol II-Gdown1-RPAP2-GPN1-GPN3 complex, GPN1-GPN3 facilitates the recycling of RPAP2 to the cytoplasm for further rounds of biogenesis.

Based on these insights, we speculate that GPN1-GPN3 may function in Pol II biogenesis as a marker and molecular switch facilitating the assembly process. GPN1-GPN3 have been found to associate with partially assembled Pol II complexes, particularly a RPB2- and Gdown1-containing subcomplex before the incorporation of RPB1^1^. Thus, GPN1-GPN3 might associate first with Pol II subcomplexes, possibly via interaction with RPB2 or a chaperone-associated factor, such as URI1. URI1 has been predicted to bind not only Gdown1, but also GPN1 and GPN3^40^. Early assembly factors, for example URI1, or exposed hydrophobic peptides may act as guanine nucleotide exchange factors to keep GPN1-GPN3 in a predominantly GTP-hydrolyzing state, potentially facilitating a chaperone function. Once assembly of the RPB2-subcomplex is complete, GPN1-3 would revert to a GDP-bound state, facilitating high-affinity interaction with RPAP2. RPAP2 could facilitate the joining of the RPB1 subcomplex and remain bound to assembled Pol II through the DNA-binding cleft. This would be consistent with previous genetic work in yeast suggesting that the yeast RPAP2 homolog Rtr1 cooperates with the yeast GPN1-GPN3 homologs (Npa3, Gpn3) to facilitate the assembly of the two largest subunits of Pol II^46^. RPAP2 would then anchor GPN1-3 to assembled Pol II-Gdown1 and all proteins would translocate with Pol II into the nucleus. GPN1-GPN3 would then enable RPAP2 recycling to the cytoplasm by virtue of an NES present within GPN1 (Fig. 6). Further work will be required to elucidate the proteins, which recognize GPN1-GPN3 in the GTP- and GDP-bound states and to clarify the role of the GPN1-GPN3 complex as a chaperone.

## Methods

### Endogenous protein tagging

Wild-type K562 cells (DSMZ) were edited to express an eGFP–3C–Gdown1 fusion protein (final construct Blasticidin - P2A - EGFP-GS linker - AID degron - GS linker − 3C protease site - Gdown1) using a modified CRISPR–Cas9 knock-in protocol as previously described^47^. In brief, the gRNA (5 -AGAATGTGCTCGCTGCCCCG-3′) was inserted into the pLCG (hU6-sgRNA-EFSSpCas9-P2A-mCherry) plasmid and 500 bp regions flanking the Gdown1 start codon from K562 genomic DNA were cloned into a pLPG vector. K562 cells were co-transfected with the HDR donor and Cas9 plasmids and subjected to blasticidin selection (10 μg/mL, Invitrogen). GFP-positive cells were isolated by FACS using a BD FACSAria III (BD Biosciences) and genotyped with primers GCCAGAGTCTCCCAAATCCT and ACAAGTGTCTCGTGGGAAGT. Expression of GFP–Gdown1 was verified by western blot using anti-POLR2M (HPA068141, Sigma Aldrich, 1:1000 dilution) and anti-GFP (sc-9996, Santa Cruz, 1:1000 dilution) antibodies.

### Cell fractionation

For extract preparation, human K562 cells endogenously expressing GFP-AID-3C-Gdown1 were cultured in 12 L RPMI media, 10% FBS (Gibco), 2% GlutaMax (Gibco), 1% sodium pyruvate (Sigma Aldrich) and 1% penicillin-streptomycin (Sigma Aldrich). Cytoplasmic extract (CE) and nuclear extract (NE) were prepared as previously described^48^ and dialyzed against Buffer E (20 mM HEPES-KOH pH 8.2 at 4 °C, 20% (v/v) glycerol, 100 mM KCl, 0.2 mM EDTA, 0.5 mM DTT). Nuclear and cytoplasmic separation was confirmed by Western blot using antibodies against GAPDH1 (MA1-16757, Sigma Aldrich, 1:5000 dilution) and U1snRNP70 (sc-9571, Santa Cruz Biotechnology, 1:1000 dilution) as cytoplasmic and nuclear makers respectively.

### Preparation of GFP nanobody-coupled resin

A plasmid encoding a GFP-nanobody carrying a C-terminal 6xHis tag was a gift from Brett Collins (Addgene plasmid # 49172; RRID:Addgene_49172)^49^. The coding sequence was altered using site-directed mutagenesis to contain two lysine residues between the C-terminus of the insert and the 6xHis tag (GFP nanobody-KK-6xHis), to improve coupling to AminoLink Plus resin. The modified GFP nanobody was expressed in E. coli BL21(DE3)RIL cells. The culture was grown to an optical density 600 nm (OD600) of 0.7 at 37 °C and then induced by 0.5 mM IPTG for 16 h at 18 °C. Cells were collected and resuspended in NaPi buffer (50 mM sodium phosphate pH 7.4 at 25 °C, 200 mM NaCl) with addition of protease inhibitors (PIs, 1 mM PMSF, 1 mM benzamidine, 60 μM leupeptin, and 200 µM pepstatin) and 10 mM imidazole, then sonicated on ice. GFP nanobody was purified using a HisTrap column HP (Cytiva) equilibrated in NaPi buffer with PIs and 10 mM imidazole. The column was washed with NaPi buffer containing 30 mM imidazole, incubated with ATP Wash buffer (NaPi buffer, 30 mM imidazole, 5 mM ATP, 2 mg/mL denatured *E. coli* protein) at 25 °C to remove chaperones and then equilibrated back to 4 °C. GFP nanobody was eluted using a linear gradient of imidazole in NaPi buffer (30 mM to 500 mM). Peak protein fractions were subjected to size exclusion chromatography using a Superdex Increase 75 10/300 GL (Cytiva) equilibrated in Coupling buffer (50 mM sodium phosphate pH 7.2 at 25 °C, 150 mM NaCl). Purified GFP nanobody was concentrated using 10,000 MWCO Amicon Ultra Centrifugal Filter (Merck Millipore) and stored at −80 °C until use.

The GFP-nanobody resin was prepared using purified GFP nanobody coupled to AminoLink Plus resin (0.3 mg/100 uL resin, Thermo Scientific) according to the manufacturer’s protocol and stored in 1x PBS supplemented with 0.02% sodium azide at 4 °C.

### Purification of human Gdown1-associated proteins

The GFP-nanobody resin (homemade for biochemical and structural experiments, GFPtrap (Chromotek) for western blot and mass spectrometry experiments) was first equilibrated using Buffer A (20 mM HEPES-NaOH pH 7.9 at 25 °C, 100 mM NaCl, 0.05% Triton X-100, 2 mM MgCl_2_, 10% (v/v) glycerol, 2 mM DTT). CE or NE from wild-type (control) or GFP-tagged Gdown1 cells was then applied to the resin after dilution 1:1 with Dilution buffer (20 mM HEPES-KOH pH 7.9 at 25 °C, 7.5 mM KCl, 3 mM MgCl_2_, 1 mM DTT) and incubated for 2 h at 4 °C. The resin with bound proteins was washed Buffer A and once with Buffer B (20 mM HEPES-NaOH (pH 7.9 at 25 °C), 100 mM NaCl, 2 mM MgCl_2_, and 2 mM DTT). Protein elution was performed using 3 °C protease for 2 hours at 4 °C. Proteins intended for mass spectrometry analysis were flash-frozen in liquid nitrogen and stored at −80 °C. Cytoplasmic RNA polymerase II complexes destined for XL-MS or cryo-EM were subjected to additional processing as detailed below.

### Mass spectrometry analysis of Gdown1-associated proteins

Samples (CE GFP-tagged, CE control, NE GFP-tagged, NE control) were each prepared in triplicate. Digestion, reduction, and alkylation were carried out as previously described^50^ (project ID: PXD069399). The nano HPLC system (UltiMate 3000 RSLC nano system, Thermo Fisher Scientific) was coupled to an Q Exactive HF-X mass spectrometer equipped with a Proxeon nanospray source. Peptides were loaded onto a trap column (PepMap Ac-claim C18, 5 mm × 300 μm ID, 5 μm particles, 100 Å pore size, Thermo Fisher Scientific) at a flow rate of 25 μl/min using 0.1% trifluoroacetic acid (TFA) as mobile phase. Peptides were analyzed on an analytical column (PepMap Acclaim C18, 500 mm × 75 μm ID, 2 μm, 100 Å, Thermo Fisher Scientific) with a linear gradient from 2-35% B (80% acetonitrile (ACN), 0.1% formic acid (FA)). The Q Exactive HF-X mass spectrometer was operated in data-dependent mode, performing a full scan (m/z range 380–1500, resolution 60,000), followed each by MS/MS scans of the most abundant ions. MS/MS were acquired with an isolation window of 1.0 m/z and fragmentation was achieved with HCD with a collision energy of 28% NCE. For peptide identification, the RAW files were loaded into Proteome Discoverer (version 2.3.0.523, Thermo Scientific). All MS/MS spectra were searched using MSAmanda v2.0.0.12368^51^. For the protein-identification-set, the peptide mass tolerance was set to ±5 ppm and fragment mass tolerance to ±8 ppm, the maximum number of missed cleavages was set to 2, using tryptic enzymatic specificity with proline restriction. Peptide and protein identification was performed in two steps. First, the RAW-files were searched against the human_uniprot_reference_2020-12-05.fasta with beta-methylthiolation on cysteines as fixed modification. The result was filtered to 1% FDR on protein level using the Percolator algorithm^52^. Proteins were filtered to be identified by a minimum of 2 PSMs in 1 sample. Peptides were subjected to label-free quantification using IMP-apQuant^53^. Proteins were quantified by summing unique and razor peptides and applying intensity-based absolute quantification (iBAQ)^54^. Protein-abundance normalization was done by sum normalization of the MaxLFQ algorithm^55^. Identified proteins were filtered to a minimum of 2 peptides. Statistical significance of differentially expressed proteins was determined by limma^56^.

### Cryo-electron microscopy of cytoplasmic Pol II complexes

Cytoplasmic Pol II complexes, recovered using GFP-nanobody affinity resin as described above, were purified using gradient fixation (GraFix)^57^. Briefly, sucrose gradients were prepared using stepwise layering. Light sucrose solution (5% sucrose, 20 mM HEPES-NaOH pH 7.9 at 25 °C, 100 mM NaCl, 2 mM MgCl_2_, 2 mM DTT) and heavy sucrose solution (30% sucrose, 20 mM HEPES-NaOH pH 7.9 at 25 °C, 100 mM NaCl, 0.05% glutaraldehyde) were prepared and gradients were assembled by adding successive layers containing 30%, 23.75%, 17.5%, 11.25% and 5% sucrose (w/v). Each layer was flash-frozen on dry ice for 5 minutes prior to the addition of the next. Once all layers were in place, the gradients were allowed to thaw slowly overnight at 4 °C. Gradient preparation used for separation of cytoplasmic Pol II for further mass-spectrometry band identification did not contain glutaraldehyde. Centrifugation was performed at 32,000 rpm for 16 h at 4 °C. Gradients were fractionated, and the fractions containing Pol II complexes were pooled. To quench residual crosslinker, 50 mM L-lysine (pH 7.8 at 25 °C) was added at 25 °C. Samples were then dialyzed for 24 h at 4 °C in buffer containing 20 mM HEPES-NaOH pH 7.9 at 25 °C, 100 mM NaCl, 2 mM MgCl_2_, and 2 mM DTT. Final concentration of the dialyzed complexes was achieved using 100 kDa MWCO Amicon Ultra centrifugal filters (Merck Millipore). Cytoplasmic Pol II complexes were then applied to wells and overlaid with a 2 nm carbon film, followed by a 30-minute incubation at 4 °C. Quantifoil Cu 300 R1.2/1.3 grids were then floated onto the sample surface to adsorb the carbon layer. Afterwards, grids were vitrified in liquid ethane using a Vitrobot Mark IV (Thermo Scientific, 100% humidity, 4 °C). Cryo-EM grids were loaded into a Thermo Fisher Titan Krios G3i transmission electron microscope (TEM) operating at 300 kV and equipped with a Gatan K3 BioQuantum direct electron detector, using an energy filter with a 10 eV slit width. Data acquisition was performed with EPU software (version 2.11). Images were captured at a nominal magnification of ×130,000, corresponding to a physical pixel size of 0.656 Å. After two collection sessions, a total of 43,150 micrographs were acquired with a defocus range of –0.3 to –3.5 μm. The electron exposure rate was approximately 40.4 e^−^/Å²/s, with each exposure lasting 1.86 s, yielding a cumulative dose of 75 e^−^/Å² across 50 frames, and with an exposure rate on the detector of ~15–17 e^−^/pix/s.

Micrographs were pre-processed (motion correction and CTF estimation) and binned 2-fold to a final pixel size of 1.312 Å using Warp (version 1.0.9)^58^. Particle coordinates were selected using Warp, and dose-weighted particles were extracted and imported into RELION 3.1 for further processing. Data from two collection sessions were independently cleaned using a combination of 2D and 3D classification in RELION 3.1 (Supplementary Fig. 2). The two data sets were merged and subjected to CTF refinement (per-particle defocus and per-micrograph astigmatism) beam tilt refinement, and particle polishing, before a second round of defocus and astigmatism fitting. The resulting refined density was used to generate the model of the Pol II core (excluding the stalk and clamp domains), whereas 3D classification in RELION 3.1 and RELION 5.0 was utilized to improve densities for the modeling of RPAP2 and Gdown1 (Table 1 and Supplementary Fig. 2). Local resolution was calculated in cisTEM (2.0.0 alpha) using the non-fixed resolution estimator described in Rohou^59^. ChimeraX (version 1.10)^60^ was used for visualization.

**Table 1:** Cryo-EM data collection, refinement, and validation statistics

	Pol II-Gdown1-RPAP2 Pol II core map (EMD-55578)	Pol II-Gdown1-RPAP2 Pol II stalk map (EMD-55579)	Pol II-Gdown1-RPAP2 RPAP2 map (EMD-55580)	Pol II-Gdown1-RPAP2 Gdown1 N-term map (EMD-55581)	Pol II-Gdown1-RPAP2 Gdown1 C-term map (EMD-55582)	RPAP2- GPN1-GPN3 (EMD-55585)
Data collection and processing						
Magnification	×130,000	×130,000	×130,000	×130,000	×130,000	×165,000
Voltage (kV)	300	300	300	300	300	300
Electron exposure (e^–^/Å^2^)	75	75	75	75	75	80.3
Defocus range (μm)	−0.3 to −3.5	−0.3 to −3.5	−0.3 to −3.5	−0.3 to −3.5	−0.3 to −3.5	−0.3 to −3
Pixel size (Å)	0.65	0.65	0.65	0.65	0.65	0.5
Symmetry imposed	C1	C1	C1	C1	C1	C1
Initial particle images (no.)	3,496,036	3,496,036	3,496,036	3,496,036	3,496,036	4,950,742
Final particle images (no.)	887,711	117,242	54,739	85,840	127,728	532,839
Map resolution (Å)^a^	2.7 (2.7)	3.1 (3.1)	3.3 (4.0)	2.9 (2.9)	2.8 (2.8)	2.8 (2.8)
FSC threshold	0.143	0.143	0.143	0.143	0.143	0.143
Map resolution range (Å)	2.6–8.0	2.6–8.0	2.6–8.0	2.6–8.0	2.6–8.0	2.3–8.0
Map sharpening *B* factor (Å^2^)^a^	−45.1 (−45.1)	−43.1 (0)	−43.9 (0)	−25.0 (0)	−24.8 (0)	−73.7 (−50)
Refinement	Pol II-Gdown1-RPAP2 (9T5H)					RPAP2-GPN1-GPN3 (9T5J)
Initial models used (PDB code)	8QEP, 9BZ0, 7B7U					
Model resolution (Å)	2.7	3.1	3.2	2.9	3.0	3.0
FSC threshold	0.5	0.5	0.5	0.5	0.5	0.5
Model–map correlation coefficients CC (masked)^b^	0.85	0.85	0.83	0.87	0.81	0.79
Model composition						
Non-hydrogen atoms	29333					5525
Protein residues	3651					684
Ligands	6					2
Nucleotide	-					2
*B* factors (Å^2^)						
Protein	92.25					82.40
Ligand	170.31					55.65
Nucleotide						32.58
R.m.s. deviations						
Bond lengths (Å)	0.004					0.005
Bond angles (°)	1.047					0.742
Validation						
MolProbity score	1.08					1.24
Clashscore	1.64					2.53
Poor rotamers (%)	0.99					0.32
Ramachandran plot						
Favored (%)	97.03					96.72
Allowed (%)	2.94					3.28
Disallowed (%)	0.03					0.00

^a^The resolutions and sharpening factors of the densities used for modeling are indicated in parentheses.

^b^Correlations were calculated for the Pol II core (well-resolved in all maps) plus the region of interest.

### Pol II-Gdown1-RPAP2 model building and refinement

A starting model for Pol II was generated by mutating a previously published model of porcine Pol II lacking the clamp domain (8QEP^61^) to include the corresponding human residues (T74I, S126T). A starting model for the Pol II stalk (RPB4/RPB7) was obtained from a previously published high-resolution Pol II structure (9BZ0^62^). Prediction of a Pol II (no clamp)-Gdown1-RPAP2 structure using AlphaFold3^36^ was combined with a previously solved Pol II-RPAP2 structure (7B7U^23^) in order to assign an identity to residues previously designated as “unknown”. An AlphaFold3 prediction of Pol II (no clamp) bound to Gdown1 was used to generate a starting model for Gdown1.

After fitting the starting model into the core density, areas of poor fit or geometry were adjusted using Isolde (version 1.10.1)^63^ and real space refined using Phenix (version 1.21.2)^64^, applying Isolde-generated restraints (global minimization with reference restraints and ADP refinement). This procedure was used to fit the RPAP2 and Gdown1 N-terminus (15-68) models into the densities. The model for the Gdown1 C-terminus (226-336) was adjusted using Isolde prior to Phenix real-space refinement.

The Pol II core was well-resolved in all classified densities, and was included in all refinements to maintain the interfaces between Pol II and RPAP2 or Gdown1. The final Pol II-Gdown1-RPAP2 model was generated by aligning the Pol II core of all refined models, then merging the fitted RPAP2 and refined Gdown1 regions into a composite model.

### Computational structure prediction

Pol II structure prediction was carried out using the following sequences (NCBI or Uniprot accession codes in parentheses): RPB1 (NP_000928.1), RPB2 (NP_000929.1), RPB3 (NP_116558.1), RPB4 (NP_004796.1), RPB5 (NP_002686.2), RPB6 (NP_068809.1), RPB7 (NP_002687.1), RPB8 (NP_006223.2), RPB9 (NP_006224.1), RPB10 (NP_066951.1), RPB11 (NP_006225.1), RPB12 (NP_005025.1). In all predictions, the unstructured regions RPB6 1-45 and RPB1 1488-1970 were omitted. The majority of the Pol II clamp domain (RPB1 1-360, RPB2 1062-1174) was omitted from the Pol II-TTF2 prediction. The full Pol II clamp domain (RPB1 1-360, RPB1 1414-1466, RPB2 1062-1174), unresolved in the cytoplasmic Pol II cryo-EM structure, was omitted from one Pol II-GPN1-GPN3 prediction. RPAP2-GPN1-GPN3 structure prediction was carried out using the following sequences (Uniprot accession codes in parentheses): RPAP2 (Q8IXW5), GPN1 (Q9HCN4), and GPN3 (Q9UHW5). The Pol II-GPN1-GPN3 (containing or lacking the clamp domain), Pol II-TTF2 (UniProt Q9UNY4), and RPAP2-GPN1-GPN3 structures were predicted using the AlphaFold3 webserver^36^. Visualization and calculation of Coulombic electrostatic potentials were carried out using UCSF ChimeraX^60^.

### Constructs for recombinant RPAP2 and GPN1-GPN3 expression

The open reading frame of human RPAP2 (UniProt Q8IXW5) was cloned into a modified pFastBac-derived vector (438-C) containing an N-terminal 6xHis-MBP tag followed by a 3C protease site^65^. A truncated version of RPAP2, encompassing amino acids 1–215, was cloned into pOPINB *E. coli* vector with an N-terminal 6xHis and 3C protease site^66^. Human GPN1 (UniProt Q9HCN4) was cloned into a second modified pFastBac-derived vector (438-B) to generate a construct containing an N-terminal 6xHis-3C protease site, whereas GPN3 (UniProt Q9UHW5) was cloned into the vector lacking any tag. GPN1 and GPN3 were further subcloned into the same 438 vector using ligation-independent cloning as previously described^65^.

### Protein expression and purification

GPN1 and GPN3 were co-expressed in insect cells. Cells were resuspended in GPN buffer (20 mM HEPES-NaOH pH 7.9 at 25 °C, 200 mM NaCl, 1 mM DTT) with 30 mM imidazole and PIs and sonicated on ice. Cleared lysate was applied to a HisTrap HP column (Cytiva) equilibrated in GPN buffer supplemented with 30 mM imidazole and PIs. Column was washed with GPN buffer with 50 mM imidazole. GPN1-GPN3 complex was eluted with a linear gradient of 50 mM to 500 mM imidazole and cleaved with 3 C protease over night while dialyzing against GPN buffer and 30 mM imidazole. Cleaved proteins were applied to a HisTrap column and flow-through collected. Proteins were separated on a Superdex Increase 75 10/300 GL column (Cytiva) equilibrated in GPN size exclusion buffer (20 mM HEPES pH 7.9 at 25 °C, 100 mM NaCl, 2 mM MgCl_2_, 1 mM DTT). Peak fractions were pooled. For purification of His-GPN1-GPN3 complex for pulldown experiments, protease cleavage and reverse Ni-NTA steps were omitted.

Full-length RPAP2 was expressed in insect cells as previously described^23^. The RPAP2 Pol II-binding domain (RPAP2 (1-215)) was expressed in E. coli BL21(DE3)RIL cells. Cells were grown in LB with 30 µg/mL kanamycin and 100 µg/mL chloramphenicol to OD600 0.7 and protein expressed adding 0.5 mM IPTG for 16 h at 18 °C. RPAP2 full-length and RPAP2 (1-215) protein were purified as described previously with small changes^23^. Briefly, cells were lysed by sonication in RPAP2 buffer (25 mM Tris-HCl pH 8.0 at 25 °C, 500 mM NaCl, 30 mM imidazole, 10% (v/v) glycerol, 10 µM ZnCl_2_, 1 mM DTT) with addition of PIs. The cleared lysates were applied to a HisTrap HP column (Cytiva) equilibrated in RPAP2 buffer with PIs. Two MBPtrap columns (Cytiva) equilibrated in RPAP2 buffer were connected in tandem to the HisTrap column and bound proteins were eluted from HisTrap column using linear gradient of RPAP2 buffer from 30 mM to 500 mM imidazole. Afterwards, MBPTrap columns were washed with RPAP2 buffer and elution performed in RPAP2 buffer containing 100 mM maltose. RPAP2 fractions were pooled and cleaved with 3C protease over night at 4 °C and re-applied on HisTrap column equilibrated in RPAP2 buffer. Flow-through was collected and applied on a Superdex Increase 200 10/300 column (Cytiva) equilibrated in RPAP2 size exclusion buffer (20 mM HEPES-NaOH pH 7.9 at 25 °C, 200 mM NaCl, 10% (v/v) glycerol, 10 µM ZnCl_2_, 1 mM DTT). Fractions containing RPAP2 were pooled and concentrated using a 30,000 MWCO Amicon Ultra Centrifugal Filter (Merck Millipore). RPAP2 (1-215) was purified using the same procedure, omitting the MBPtrap column step.

Porcine Pol II and human Gdown1 were purified as previously described^25^, except that for Gdown1 expression, the culture was grown to OD600 0.7 at 37 °C before induction of expression by addition of 1 mM IPTG for 6 hrs at 25 °C. To prepare Pol II-Gdown1 complex, Pol II was incubated with human Gdown1 in a 1:3 molar ratio and incubated at 4 °C for 30 min, prior to purification over a Sephacryl S-300 HiLoad column equilibrated in Pol II buffer (150 mM NaCl, 5 mM HEPES-KOH pH 7.25 at 25 °C, 10 μM ZnCl_2_, 10 mM DTT). Peak fractions were concentrated as described above. All proteins were aliquoted and frozen in liquid nitrogen prior to storage at −80  °C.

### GTPase activity assay

GTPase activity was measured using the Malachite Green Phosphate Assay kit (Sigma-Aldrich) following the manufacturer’s instructions. 0.66 µM GPN1-GPN3 was incubated with 0.25 mM GTP (Thermo Fischer Scientific) for 60 min at 37 °C in GPN size exclusion buffer. Values were corrected for free phosphate contamination arising from buffer, GPN1-GPN3, and GTP using appropriate controls. Reactions were carried out in triplicate.

### Pull-down interaction assays

His-GPN1-GPN3 and Pol II or Pol II EC were mixed in 1:2 molar ratio in Low salt buffer (20 mM HEPES pH 7.9 at 25 °C, 100 mM NaCl, 2 mM MgCl_2_, 1 mM DTT) and incubated for 30 minutes at 4 °C and then applied on MagneHis beads (Promega) for 30 minutes at 4 °C. Unbound proteins were washed away with High salt wash buffer (20 mM HEPES pH 7.9 at 25 °C, 300 mM NaCl, 2 mM MgCl_2_, 1 mM DTT).

0.66 µM HisGPN1-GPN3 was incubated with 10 mM GTP (Thermo Scientific), 10 mM GMPPNP (Jena Bioscience) or 10 mM GDP (Jena Bioscience) for 30 min at 37 °C and washed once with Low salt buffer. 0.72 µM RPAP2 or 1.3 µM RPAP2(1-215) with addition of 10 mM GTP, 10 mM GMPPNP or 10 mM GDP was added to the beads and incubated for 30 min at 4 °C. Beads were washed with High salt wash buffer supplemented with 30 mM imidazole. Immobilized proteins were eluted with Low salt buffer supplemented with 500 mM imidazole. The proteins were analyzed by SDS-PAGE gel stained with Coomassie. All pull-down assays were performed in triplicate.

### Pol II-Gdown1-RPAP2-GPN1-GPN3 reconstitution and size exclusion analysis

Pol II, Gdown1, RPAP2 and GPN1-GPN3 were incubated in a 1:3:3:3 molar ratio for 30 min at 4 °C. Sample was loaded onto a Superdex 200 Increase 3.2/300 size exclusion column (Cytiva) equilibrated in buffer containing 20 mM HEPES-NaOH pH 7.9 at 25 °C, 100 mM NaCl, 2 mM MgCl_2_, 10 µM ZnCl_2_, 1 mM DTT. The peak fractions were loaded on an SDS-PAGE gel, which was subsequently stained with Coomassie (InstantBlue).

### Mass photometry

For Pol II complex formation, Pol II and Pol II-Gdown1 complex with GPN1-GPN3 and/or RPAP2 was prepared by incubating the proteins in 1:2:2 molar ratio for 30 minutes at 4 °C. Samples were diluted to a final concentration of 100 nM using Dilution buffer (20 mM HEPES-NaOH pH 7.9 at 25 °C, 100 mM NaCl, 2 mM MgCl_2_, 10 µM ZnCl_2_, 1 mM DTT). Samples and respective controls were measured using Refeyn Two MP mass photometer (Refeyn Ltd) at a final concentration 20 nM. The movies were collected for 60 s. The movies were processed with DiscoverMP software. The instrument was calibrated using MfP1 standard (Refeyn Ltd) before each measurement.

For RPAP2-GPN1-GPN3 assembly experiments performed without added nucleotide, GPN1-GPN3 and RPAP2 were added in a 1:1 molar ratio and incubated for 30 min at 4 °C prior to measurement. For nucleotide mass-photometry experiments, GPN1-GPN3 was incubated with 10 mM GTP (Thermo Scientific), 10 mM GMPPNP (Jena Bioscience) or 10 mM GDP (Jena Bioscience) for 30 min at 37 °C and then RPAP2 was added in a 1:1 molar ratio and incubated for 30 min on ice. All samples were measured in triplicate. Significance was calculated using GraphPad Prism 10.

### Chemical crosslinking and mass spectrometry

Cytoplasmic Pol II samples were enriched using ProteoMiner protein enrichment kit (Bio-Rad Laboratories). For crosslinking, 50 µL of disuccinimidyl sulfoxide (DSSO, Cayman Chemical) was added at a final concentration of 1.5 mM and incubated for 6 minutes and quenched with 3 µL of 1 M ammonium bicarbonate (Sigma-Aldrich). For the in vitro reconstituted sample, Pol II, Gdown1, RPAP2 and GPN1-GPN3 were mixed in a molar ratio of 1:3:2:2 and incubated for 30 minutes at 4 °C. The complex was incubated for 30 min with 3 mM BS3 (Thermo Fisher) at 4 °C and quenched with a final concentration of 60 mM ammonium bicarbonate (Sigma-Aldrich).

The crosslinked proteins were denatured by adding 8 M urea and reduced and alkylated using TCEP and IAA respectively. Samples were digested using Lys-C (FUJIFILM Wako Chemicals U.S.A. Corporation) for 1 h and after dilution to 1 M urea with trypsin (Promega) overnight. Afterwards samples were acidified with TFA, peptides were cleaned using Sep-Pak tC18 1 cc Vac Cartridges (Waters) and dried in a vacuum centrifugation. Peptides were dissolved in 20 µL SEC buffer (77.5 % H2O, 22.5 % ACN, 0.1 % TFA) and loaded onto a Superose 30 Increase column (Cytiva) to enrich fractions containing crosslinked peptides^67^. Samples containing crosslinked peptides were dried and dissolved in mobile phase A (98% H2O, 2% ACN, 0.1% FA) and injected onto an Ultimate 3000 RSLCnano System coupled to an Orbitrap Eclipse Tribrid Mass Spectrometer (both Thermo Fisher Scientific). Peptides were separated on a PepMap RSLC EASY-Spray column (C18, 2 µm, 100 Å, 75 µm x 15 cm, Thermo Fisher Scientific). Separation occurred at 300 nL·min-1 with a linear gradient from 2-35% mobile phase B (2% H2O, 98% ACN, 0.1% FA) within 60 min resulting in a total method time of 94 min. Orbitrap Eclipse was equipped with FAIMS Pro alternating between three different CVs (−50,−60,−75) operating in positive ionization mode. MS1 scans were recorded at a resolution of 60,000 @200 m/z in a range from 400-1600 m/z. Precursors with a charge of 3–8 were isolated with a 1.6 m/z window and fragmented with HCD with a stepped collision energy of 21,27 and 33 % NCE. MS2 spectra were recorded at a resolution of 30,000 @200 m/z.

DSSO crosslinked samples were searched using MS Annika^68^ within the Proteome Discoverer software suite against the same database as the BS3 samples. The predefined DSSO workflow was used and CSM and crosslink level FDR was set to 1 % and only high confidence identifications were considered for the subsequent analysis. Spectra were manually verified using the PD inbuilt spectra viewer. For BS3, raw files were converted to mgf using MSConvert^69^ and searched using xiSEARCH (v1.8.5^16^). The database consisted of the expected polymerase subunits and 40 additional proteins representing the most abundant proteins identified in a corresponding proteome profiling. To evaluate the identified crosslinks xiFDR (v2.3) was employed at a maximum Residue Pair FDR of 1% crosslink and identifications were also manually verified using xiView^70^. Crosslink bar plots and histogram plots of Cα-Cα crosslink distances were generated using xiView^70^. Crosslink mapping to structural models was performed using the XMAS ChimeraX plugin^71^.

### Cryo-electron microscopy of reconstituted RPAP2-GPN1-GPN3 complex

GPN1-GPN3 and RPAP2 were incubated in a 1:1 molar ratio for 30 min at 4 °C. Samples were subjected to size exclusion on a Superdex 200 Increase 3.2/300 column (Cytiva) equilibrated in buffer (cooled to 4 °C) containing 20 mM HEPES-NaOH pH 7.9 at 25 °C, 100 mM NaCl, 2 mM MgCl_2_, 10 µM ZnCl_2_, 1 mM DTT. Fractions containing the RPAP2-GPN1-GPN3 complex were pooled and concentrated to 0.6 mg/mL using a 30,000 MWCO Amicon Ultra Centrifugal Filter (Merck Millipore). 3.5 µL of the sample was applied to Quantifoil R0.6/1 holey carbon grids that had been glow discharged for 40 s (25 mA current, 7.0 × 10−^1 ^mbar vacuum). Using a Vitrobot Mark IV set to 100% humidity and 4 °C, grids were blotted for 7 s and immediately plunge frozen in liquid ethane and stored in liquid nitrogen. Cryo-EM grids were imaged using a Thermo Fisher Titan Krios G3i TEM operating at 300 kV and equipped with a Gatan K3 BioQuantum direct electron detector, using an energy filter with a 10 eV slit width. Data acquisition was performed using EPU software (version 2.11). Images were captured at a nominal magnification of ×165,000, corresponding to a physical pixel size of 0.50 Å. A total of 14,362 micrographs were collected with a defocus range of –0.4 to –3 μm. The electron exposure rate was 69.4 e^−^/Å²/s, with each exposure lasting 1.16 s, yielding a cumulative dose of 80.3 e^−^/Å² across 80 frames. The exposure rate on the detector was ~16 e^−^/pix/s.

Micrographs were pre-processed in RELION 5.0, and CTF estimation was performed using CTFFIND-4.1^72^. Particle coordinates were selected in cryoSPARC (version 4.4.1)^73^ using the Blob Picker. Extracted particles were cleaned first using cryoSPARC (2D classification, followed by heterogeneous refinement). To improve apparent anisotropy in the resolution of the resulting map, particles were re-imported to RELION 5.0 and subjected to additional 3D classification. Final refined densities were generated in RELION using blush regularization.

### GPN1-GPN3-RPAP2 model building and refinement

A model for the GPN1-GPN3-RPAP2 complex containing two molecules of GDP and two Mg^2+^ ions was generated using AlphaFold3. RPAP2 1-311 was deleted and the molecule was rigid body fit into the RPAP2-GPN1-GPN3 map, sharpened with a b-factor of −50, in ChimeraX. Regions of the model that did not agree with the density were deleted, and the model was manually rebuilt and adjusted in Coot^74^ and using Isolde. Unassigned density around GPN1-GPN3 was assigned to RPAP2 based on side chain densities observed in the cryo-EM map, with RPAP2-GPN1 crosslinks used for validation. Phenix real space refinement was then carried out using Isolde-suggested parameters (global minimization with reference restraints and ADP refinement). A DeepEMhancer-postprocessed map was used for display only^75^. An illustrative model of a putative Pol II-Gdown1-RPAP2-GPN1-GPN3 complex was generated using the position of GPN1 relative to RPB2 observed in the Pol II (no clamp)-GPN1-GPN3 structure prediction. The RPAP2-GPN1-GPN3 structure was aligned to GPN1, and the Pol II-Gdown1-RPAP2 structure was aligned to RPB2.

### Reporting summary

Further information on research design is available in the Nature Portfolio Reporting Summary linked to this article.

## Supplementary information

Supplementary Information

Reporting Summary

Transparent Peer Review file

## Source data

Source Data

## Data Availability

The cryo-EM maps generated in this study were deposited to the EM Data Bank under the accession codes: EMD-55583 (Pol II-Gdown1-RPAP2 composite map), EMD-55578 (Pol II-Gdown1-RPAP2 Pol II core map), EMD-55579 (Pol II-Gdown1-RPAP2 Pol II stalk map), EMD-55580 (Pol II-Gdown1-RPAP2 RPAP2 map), EMD-55581 (Pol II-Gdown1-RPAP2 Gdown1 N-terminus map), EMD-55582 (Pol II-Gdown1-RPAP2 Gdown1 C-terminus map), and EMD-55585 (RPAP2-GPN1-GPN3 map). Model coordinates were deposited to the PDBe under the accession codes: 9T5H (Pol II-Gdown1-RPAP2 complex structure) and 9T5J (GPN1-GPN3-RPAP2 structure). Immunoprecipitation-mass spectrometry and crosslinking-mass spectrometry proteomics data have been deposited to the ProteomeXchange Consortium via the PRIDE partner repository with the dataset identifiers PXD071638 and PXD070852. AlphaFold3 structure predictions have been deposited to the Zenodo repository (10.5281/zenodo.20687910). Previously published model coordinates were utilized and are available at the PDB under the accession codes 8QEP, 9BZ0, and 7B7U. Source data are provided with this paper.
