# Supplementary material for: Structure of cytoplasmic RNA polymerase II
Source: Nat Commun. 2026 Jul 13;17:8640. doi: 10.1038/s41467-026-75416-8 (PMC13486763; doi:10.1038/s41467-026-75416-8)
Supplement: Supplementary file 1 [file 41467_2026_75416_MOESM1_ESM.pdf]

Supplementary Figures

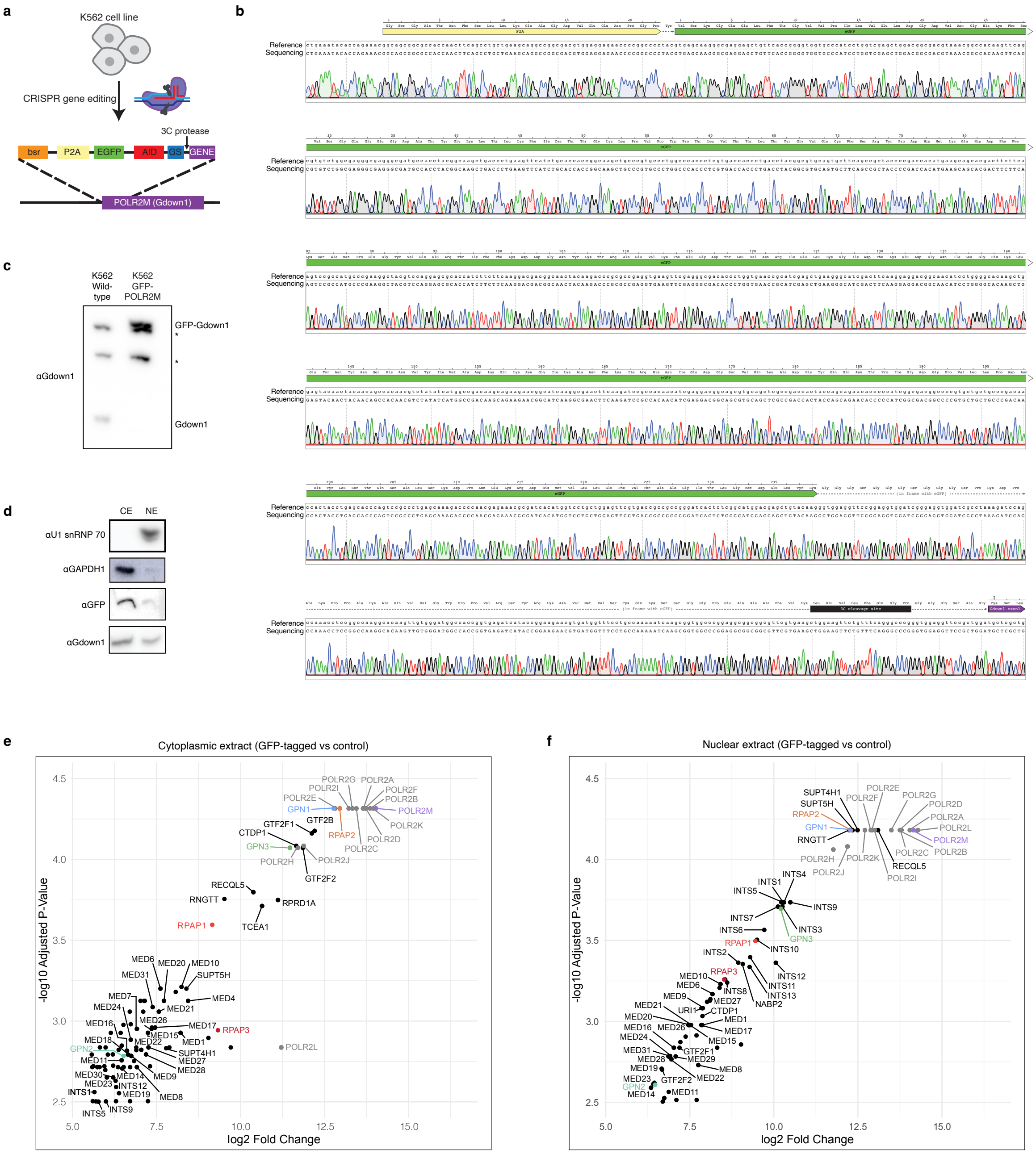

Supplementary Figure 1 Isolation and compositional analysis of endogenous Gdown1-associated complexes. **a**, Schematic of the edited Gdown1 endogenous locus in the CRISPR-edited K562 cell line. bsr, blasticidin resistance marker; P2A, intervening porcine teschovirus-1 2A; AID, auxin-inducible degron domain; GS, Gly-Ser linker. **b**, Validation of endogenous tagging by Sanger sequencing, showing the region from the introduced P2A site to the start of Gdown1. Reference, target sequence; Sequencing, Sanger sequencing result. **c**, Validation of endogenous tagging by western blotting, probing for Gdown1. Nonspecific bands labeled with an asterisk (*). The experiment was repeated three times, with similar results. **d**, Western blot analysis of representative cytoplasmic and nucleoplasmic extract preparations,

probing for the cytoplasmic marker (GAPDH), nuclear marker (U1snRNP70). Expression of Gdown1 confirmed by probing against GFP and Gdown1. The experiment was repeated four times, with similar results. **e**, Expanded zoom-in view of the Volcano plot of cytoplasmic Gdown1-enriched factors shown in Fig. 1b. Plot generated in R-studio. **f**, Expanded zoom-in view of the Volcano plot of nucleoplasmic Gdown1-enriched factors shown in Fig. 1c. Plot generated in R-studio. Pol II subunits, gray; GPN1-3, blue/green; RPAP1-2, orange/red; Gdown1 (POLR2M), purple. Source data are provided as a Source Data file.

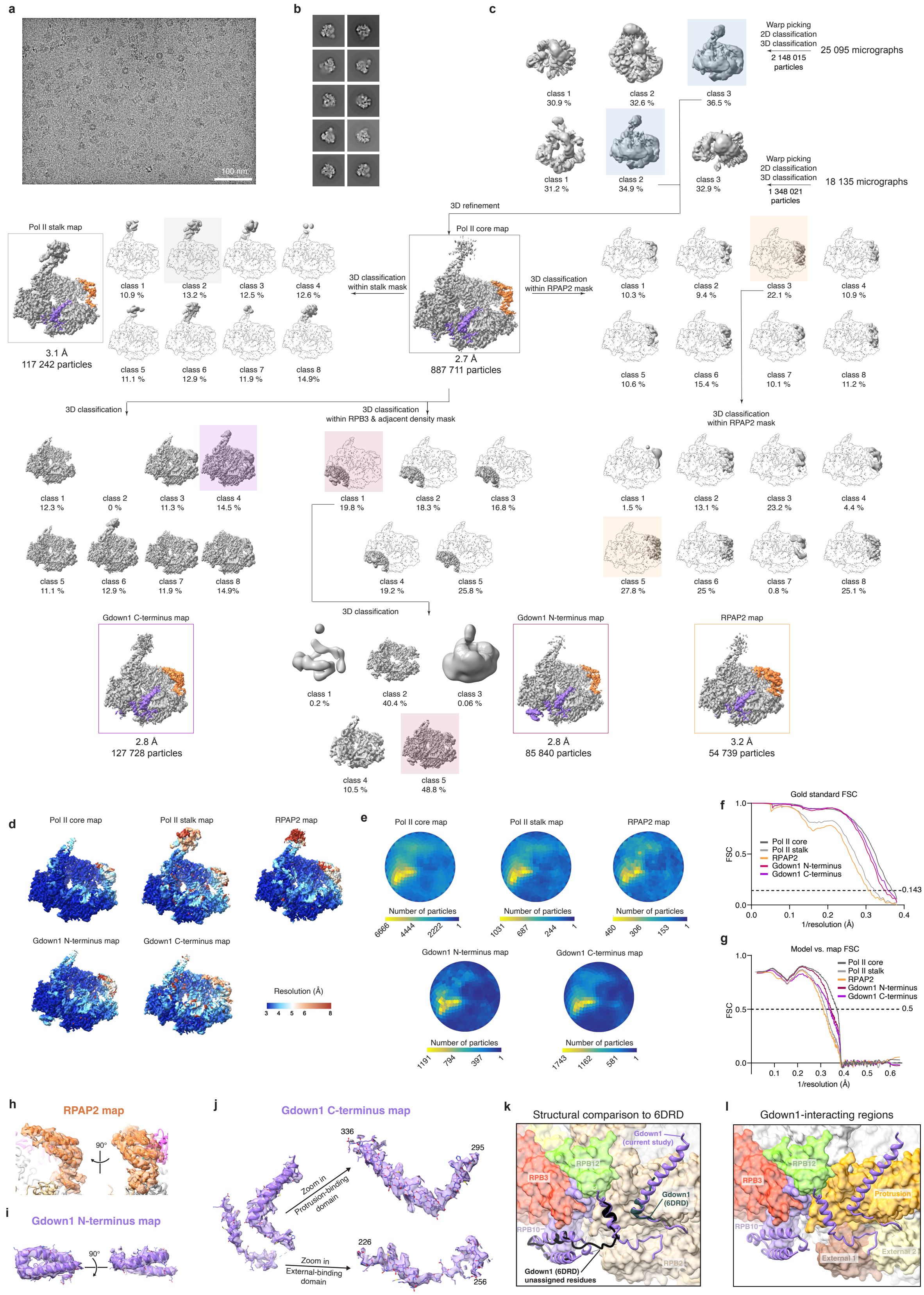

Supplementary Figure 2 Cryo-EM analysis of cytoplasmic Pol II. **a**, Representative cryo-EM micrograph field of view at -1.3 µm defocus. Scale bar 100 nm. **b**, Representative 2D classes **c**, Processing tree outlining the image processing pipeline used to generate the five final cryo-EM densities. **d**, Non-b-factor sharpened maps

colored by estimated local resolution. **e**, Angular distribution plots of the five final cryo-EM densities. **f**, Fourier shell correlation plots for the five final cryo-EM densities. **g**, Model-versus-map correlation for the five final reconstitutions. **h**, Cryo-EM density of RPAP2 with the model superimposed. **i**, Cryo-EM density of the Gdown1 N-terminus (RPB10-binding domain) with the model superimposed. **j**, Left, the Gdown1 C-terminus model overlayed with cryo-EM density. Right, zoom-ins of the protrusion- and external-binding domains of Gdown1. **k**, Comparison of the Pol II-Gdown1-RPAP2 structure with a previously published Pol II-Gdown1 structure (PDB ID 6DRD[13]). Coloring is as follows: RPB2, tan; RPB3, red; RPB10, violet; RPB12, green; Gdown1 (this study, ribbon), purple; Gdown1 (PDB ID 6DRD, ribbon): assigned residues, slate; unassigned residues, black. **l**, Regions of Pol II that interact with Gdown1. RPB2 protrusion, gold; RPB2 external 1, brown; RPB2 external 2, light yellow; RPB3, red; RPB10, violet; RPB12, green; Gdown1 (ribbon), purple.

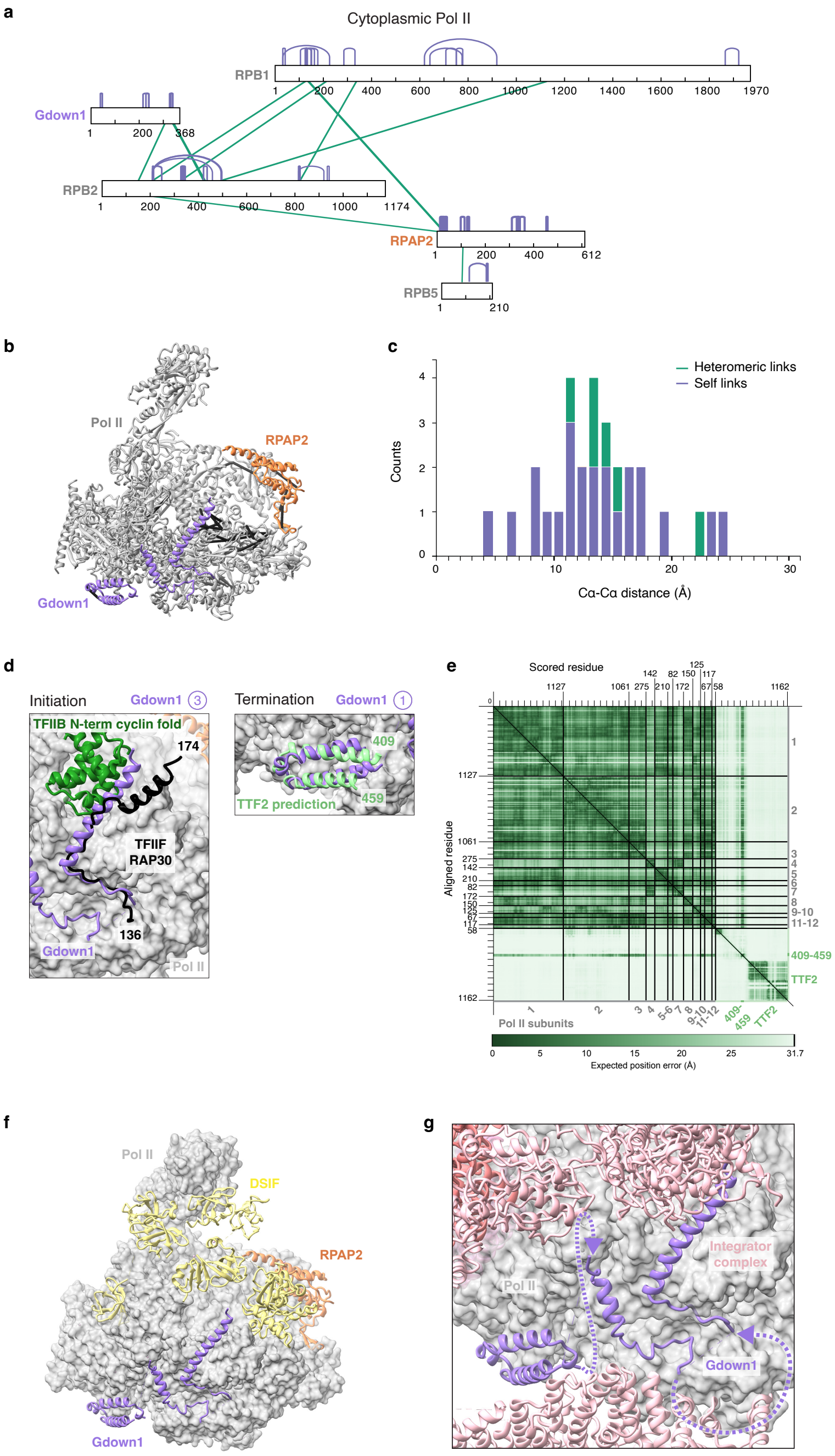

Supplementary Figure 3 Architecture of cytoplasmic Pol II and comparison to other Pol II-bound structures. **a**, Diagram of identified crosslinks in the cytoplasmic Pol II. Violet, self crosslinked peptides; green, heteromeric crosslinked peptides. **b**, Identified crosslinked peptides in the endogenous cytoplasmic Pol II sample mapped to the Pol II-Gdown1-RPAP2 structure. Pol II, gray; Gdown1, purple; RPAP2, orange; crosslinks, black. **c**, Cα-Cα distance of identified crosslinked peptides of the cytoplasmic Pol II sample mapped to the Pol II-Gdown1-RPAP2 structure. **d**, Left, superposition of the Pol II-Gdown1-RPAP2 structure with a structure of the TFIID- and Mediator-containing preinitiation complex. The Gdown1 protrusion-binding domain, TFIIB B-core N-terminal cyclin fold, and TFIIF small subunit protrusion-binding region are shown (left, PDB ID 7ENA[32]). Right, superposition of the Pol II-Gdown1-RPAP2 structure with a Pol

II-TTF2 structural prediction, showing the TTF2 region overlapping with the Gdown1 RPB10-binding domain. Colored as follows: Pol II, gray; Gdown1, purple; TFIIF, black; TFIIB, dark green; TTF2, light green. **e**, Predicted Aligned Error (PAE) plot for the Pol II (no clamp)-TTF2 structure prediction. Total residue numbers for each subunit are indicated on the top and left. Pol II subunits indicated with gray numbers. The displayed region of TTF2 (409-459) is indicated with a green bar, and the hidden region is indicated with a light green bar. pTM, 0.76; ipTM, 0.73. **f**, Superposition of cytoplasmic Pol II with DSIF (PDB ID 8UHD[75]). RPAP2, orange; DSIF, yellow; other coloring as in (c). **g,** Zoom-in comparison of the Pol II binding interfaces the Integrator complex (PDB 8RBX[29]) with those of Gdown1, as in Fig. 2d. The connectivity of unresolved regions of Gdown1 are indicated with purple dashed lines. Colored as follows: Integrator, pink; Integrator cleavage module, dark pink. Source data are provided as a Source Data file.

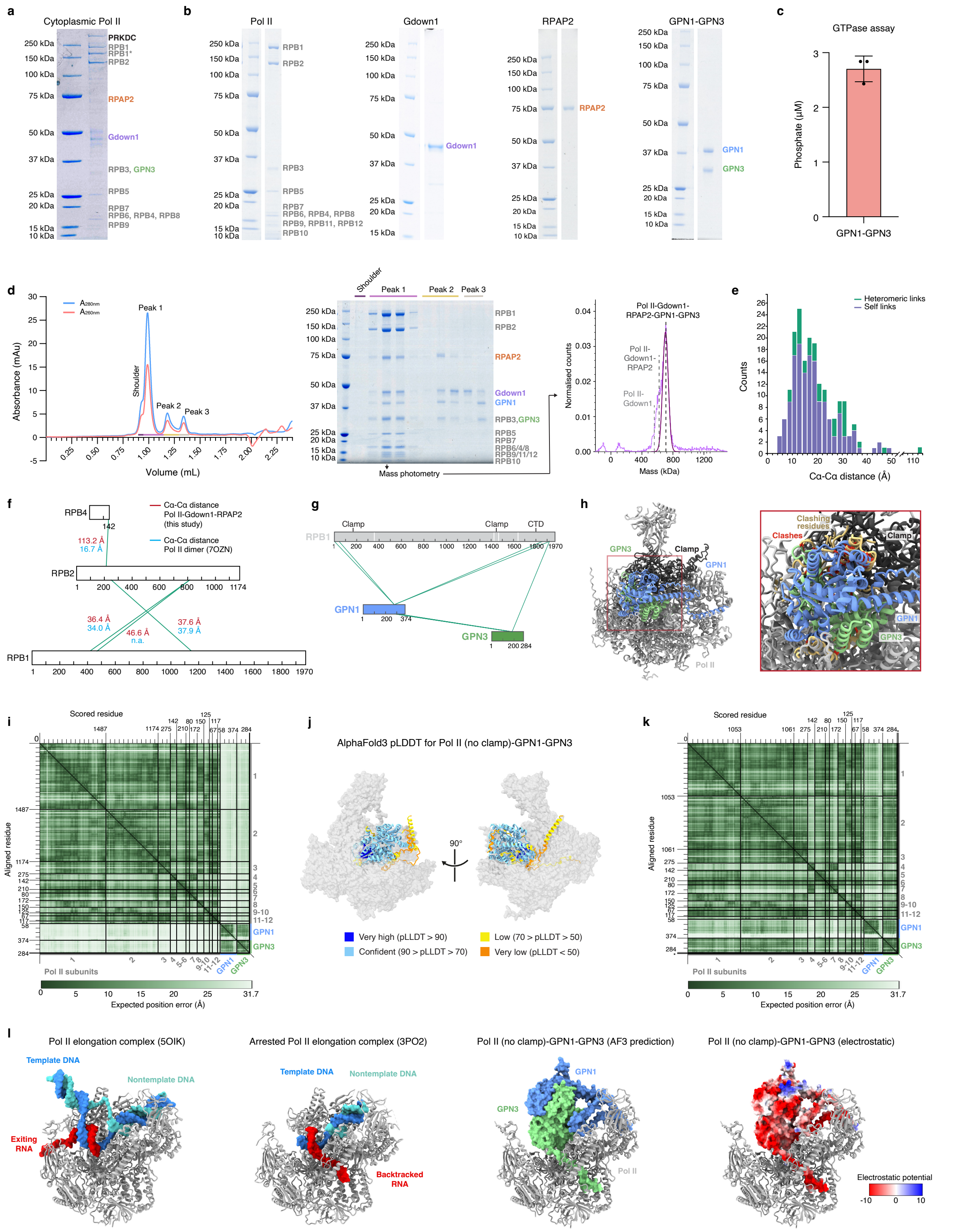

Supplementary Figure 4 Reconstitution and characterization of Pol II-Gdown1-RPAP2-GPN1-GPN3 complexes. **a**, Representative fraction of cytoplasmic Pol II after sucrose gradient purification. Proteins identified in mass-spectrometry of excised bands are labelled. RPB1* indicates a degraded form of RPB1 with the flexible C-terminal repeat domain removed (IIb form). **b**, SDS-PAGE gels showing purified Pol II, Gdown1, RPAP2 and GPN1-GPN3 complex. **c**, GTPase activity of purified GPN1-GPN3 complex. Y-axis, final concentration of released phosphate. **d**, Size exclusion chromatography of the

reconstituted Pol II-Gdown1-RPAP2-GPN1-GPN3 complex was performed three times, with similar results. Left, chromatogram; Center, SDS-PAGE analysis of the indicated fractions; Right, Mass photometry analysis of the indicated fraction. **e**, Cα-Cα distance of identified crosslinked peptides of in vitro Pol II-Gdown1-RPAP2-GPN1-GPN3 mapped to cytoplasmic Pol II structure. **f**, Comparison of Cα-Cα distances mapped to the Pol II-Gdown1-RPAP2 structure (this study) and the Pol II dimer (PDB ID 7OZN[34]), showing the one extreme outlier that is well explained by the dimer. **g**, Diagram of identified crosslinks in the in vitro Pol II-Gdown1-RPAP2-GPN1-GPN3 sample between the RPB1 subunit and the GPN1-GPN3 complex. Violet, self crosslinked peptides; green, heteromeric crosslinked peptides. **h**, Pol II-GPN1-GPN3 structure prediction. Left, model overview. Pol II, gray; GPN1, blue; GPN3, green. Right, zoom-in view. Clashes (too close contacts with overlap < 0.6 Å, H-bond distance < 0.4 Å) are shown with red lines, with Pol II residues involved in these contacts in yellow. **i**, Predicted Aligned Error (PAE) plot for the Pol II-GPN1-GPN3 structure prediction. Total residue numbers for each subunit are indicated on the top and left. Pol II subunits indicated with gray numbers. pTM, 0.82; ipTM, 0.8. **j**, Pol II (no clamp)-GPN1-GPN3 structure prediction. Pol II in gray, GPN1-GPN3 prediction colored by AlphaFold3 pLLDT confidence scores. **k**, Predicted Aligned Error (PAE) plot for the Pol II (no clamp)-GPN1-GPN3 structure prediction, labeled as in (i). Asterisk indicates an additional chain containing RPB1 residues 1467-1487. pTM, 0.87; ipTM, 0.86. **l**, Comparison of side views of the Pol II elongation complex (PDB ID 5OIK) and arrested elongation complex (PDB ID 3PO2[37]) structures to the Pol II-GPN1-GPN3 structure prediction. Nucleic acids and GPN1-GPN3 are shown in surface representation. Far right, the same view of the Pol II-GPN1-GPN3 structure prediction, with the GPN1-GPN3 surface colored by electrostatic potential. RPB2 was removed from view for clarity. Source data are provided as a Source Data file.

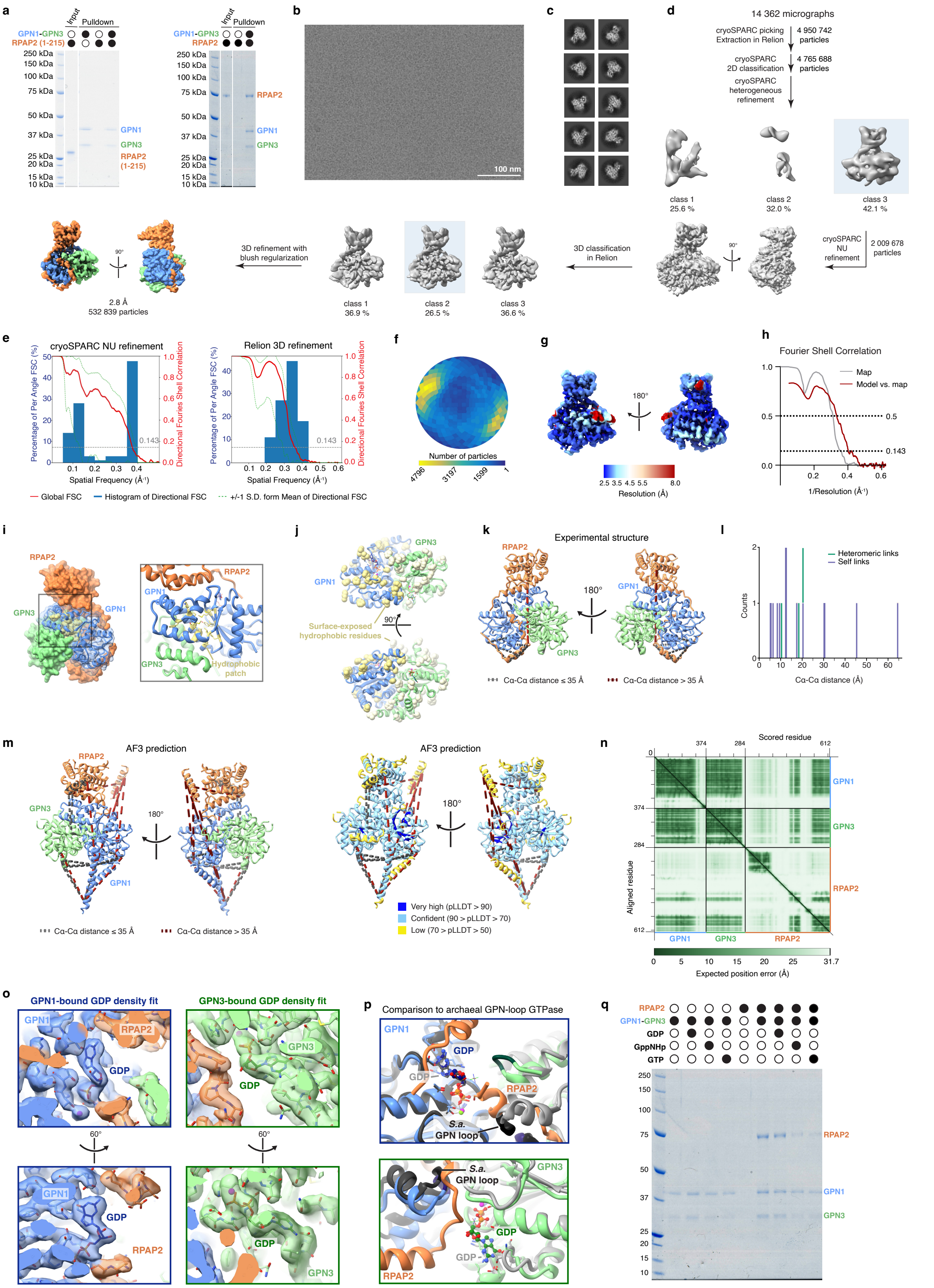

Supplementary Figure 5 Cryo-EM and biochemical analysis of the RPAP2-GPN1-GPN3 complex. **a**, Pull-down of RPAP2 and RPAP2(1-215) via immobilized His-GPN1-GPN3. Experiments were carried out in triplicate. **b**, Representative cryo-EM micrograph field of view at -1.9 µm defocus. **c**, Representative 2D classes. **d**, Processing tree outlining the

steps taken to generate the RPAP2-GPN1-GPN3 reconstruction. **e**, Directional FSC plots of the cryoSPARC NU refinement (left) and final Relion 3D refinement (right) of the RPAP2-GPN1-GPN3 complex, with sphericity values of 0.732 and 0.826, respectively. Plots generated using the 3DFSC server[78]. **f**, Angular distribution plot for RPAP2-GPN1-GPN3 final reconstruction. **g**, Non-b-factor sharpened map of RPAP2-GPN1-GPN3 colored by estimated local resolution. **h**, Fourier shell correlation plot for the final cryo-EM density, model-versus-map correlation for the final reconstruction and model. **i**, Overview and zoom-in view of a previously identified GPN1 hydrophobic patch in the context of the RPAP2-GPN1-GPN3 structure. Left, GPN1 surface shown in transparent blue overlayed on the GPN1 ribbon model. RPAP2, orange; GPN1, blue; GPN3, light green; residues of hydrophobic patch, yellow. **j**, View of the surface-exposed hydrophobic residues of GPN1-GPN3. Ala, Val, Cys, Pro, Leu, Iso, Met, Trp, and Phe residues with a solvent-accessible surface area cutoff of 25 (calculated in ChimeraX) are shown as spheres. GPN1 hydrophobic residues, yellow; GPN3 hydrophobic residues, light yellow. **k**, Identified crosslinked peptides in the in vitro Pol II-Gdown1-RPAP2-GPN1-GPN3 sample mapped to the RPAP2-GPN1-GPN3 structure. Cα-Cα distance ≤ 35 Å, gray; Cα-Cα distance > 35 Å, dark red. **l**, Cα-Cα distance of crosslinked peptides identified in the in vitro Pol II-Gdown1-RPAP2-GPN1-GPN3 sample mapped to the RPAP2-GPN1-GPN3 structure. **m**, Identified crosslinked peptides in the in vitro Pol II-Gdown1-RPAP2-GPN1-GPN3 sample mapped to the AlphaFold3-predicted RPAP2-GPN1-GPN3 structure. Very low confidence regions (pLDDT <50) were hidden for clarity. A large number of outlier crosslinks are visible, and the AlphaFold3 model failed to predict the extended interactions of RPAP2 with GPN1 and the GPN1-GPN3 interface. Left, colored by subunit; Right, colored by pLLDT confidence score, Cα-Cα distances colored as in (k). **n**, Predicted Aligned Error (PAE) plot for the RPAP2-GPN1-GPN3 prediction. Total residue numbers for each subunit are indicated on the top and left. PTM, 0.54; iPTM, 0.58. **o**, Overlay of the cryo-EM density and final RPAP2-GPN1-GPN3 model, zoomed in on the GPN1 and GPN3 GDP-binding pockets. **p**, Comparison of the human RPAP2-GPN1-GPN3 GDP binding pocket (this study) with that of the archaeal SaGPN dimer bound to GDP (PDB ID 7ZHK[39]), superimposed. Colored as follows: SaGPN monomer 1, dark gray; SaGPN monomer 2, gray; SaGPN-bound GDP, light gray, SaGPN-loop black; RPAP2-GPN1-GPN3 structure as in (a). **q**, Pull-down of RPAP2 via immobilized His-GPN1-GPN3 pre-treated with GTP, GppNHp, or GDP. The experiment was performed in triplicate. Source data are provided as a Source Data file.

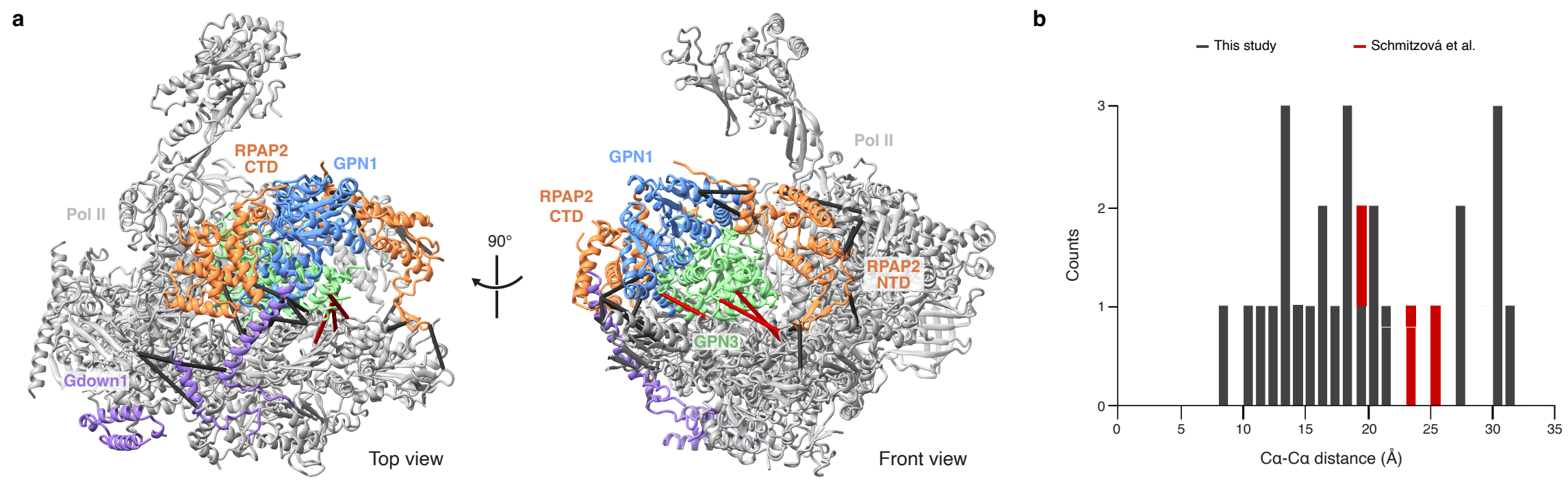

Supplementary Figure 6 Analysis of a putative Pol II-Gdown1-RPAP2-GPN1-GPN3 model. **a**, Inter-protein crosslinks observed in the in vitro Pol II-Gdown1-RPAP2-GPN1-GPN3 sample mapped to a putative model of Pol II-Gdown1-RPAP2 with GPN1-GPN3 docked on the Pol II DNA-binding cleft. Crosslinks between Pol II subunits were excluded. Colored as follows: Pol II, gray; Gdown1, purple; RPAP2, orange; GPN1, blue; GPN3, green; Inter-protein crosslinks identified in this study, dark gray; GPN3-RPB2 crosslinks identified in Schmitzová et al.[42], red. **b**, Histogram plot of Cα-Cα distances of crosslinks shown in (a).

Additional Supplementary Material

Supplementary Note 1 TFIIF association with Gdown1 purified from cytoplasmic extracts.

Despite the well characterized competition of Gdown1 and RPAP2 with TFIIF for binding to Pol II, TFIIF was identified as enriched in the Gdown1 cytoplasmic pulldown. This observation suggests that either (1) TFIIF binds to Gdown1 as part of another complex, or (2) that either Gdown1 or TFIIF can be anchored to the Pol II complex through an additional protein adapter. We observed that CTDP1, also known as TFIIF-associating CTD phosphatase 1 (FCP1), is likewise particularly enriched in cytoplasmic pulldown. From previous high-throughput pairwise protein-protein interaction prediction[40], CTDP1 has been identified as a highly confident binder of RPB7, part of the Pol II stalk, through its N-terminal region. CTDP1 is a well characterized binder of the small subunit of TFIIF through its central and C-terminal domains[79,80]. Thus, TFIIF may be bound to the Gdown1-Pol II complex via interaction with CTDP1. The interaction of CTDP1 with Pol II in the cytoplasm may help keep the RPB1 CTD of Pol II in a dephosphorylated state, in preparation for recruitment to promoters after import into the nucleus.

Supplementary Note 2 Stoichiometry of the reconstituted Pol II-Gdown1-RPAP2-GPN1-GPN3 complex

Relative band intensity of RPAP2 and GPN1 proteins after SDS-PAGE analysis and Coomassie (InstantBlue) staining of size exclusion-purified, reconstituted Pol II-Gdown1-RPAP2-GPN1-GPN3 complex suggested that these proteins might be sub-stoichiometrically associated with the complex. To quantify this, we performed mass photometry of the size exclusion-purified, reconstituted Pol II-Gdown1-RPAP2-GPN1-GPN3 complex. This analysis indicated that the majority of the purified complex corresponded to the complete assembly, with a shoulder peak signaling the presence of a small subpopulation of Pol II complexes lacking GPN1-3 and/or RPAP2.
