# Supplementary material for: Structure of cytoplasmic RNA polymerase II
Source: Nat Commun. 2026 Jul 13;17:8640. doi: 10.1038/s41467-026-75416-8 (PMC13486763; doi:10.1038/s41467-026-75416-8)
Supplement: Supplementary file 2 [file 41467_2026_75416_MOESM2_ESM.pdf]

nature portfolio

Corresponding author(s): Carrie Bernecky

Last updated by author(s): Jun 16, 2026

Reporting Summary

Nature Portfolio wishes to improve the reproducibility of the work that we publish. This form provides structure for consistency and transparency in reporting. For further information on Nature Portfolio policies, see our Editorial Policies and the Editorial Policy Checklist.

Statistics

For all statistical analyses, confirm that the following items are present in the figure legend, table legend, main text, or Methods section.

n/a	Confirmed	
☐	☒	The exact sample size (*n*) for each experimental group/condition, given as a discrete number and unit of measurement
☐	☒	A statement on whether measurements were taken from distinct samples or whether the same sample was measured repeatedly
☐	☒	The statistical test(s) used AND whether they are one- or two-sided<br>*Only common tests should be described solely by name; describe more complex techniques in the Methods section.*
☒	☐	A description of all covariates tested
☐	☒	A description of any assumptions or corrections, such as tests of normality and adjustment for multiple comparisons
☐	☒	A full description of the statistical parameters including central tendency (e.g. means) or other basic estimates (e.g. regression coefficient) AND variation (e.g. standard deviation) or associated estimates of uncertainty (e.g. confidence intervals)
☐	☒	For null hypothesis testing, the test statistic (e.g. *F*, *t*, *r*) with confidence intervals, effect sizes, degrees of freedom and *P* value noted<br>*Give P values as exact values whenever suitable.*
☒	☐	For Bayesian analysis, information on the choice of priors and Markov chain Monte Carlo settings
☒	☐	For hierarchical and complex designs, identification of the appropriate level for tests and full reporting of outcomes
☒	☐	Estimates of effect sizes (e.g. Cohen's *d*, Pearson's *r*), indicating how they were calculated

Our web collection on statistics for biologists contains articles on many of the points above.

Software and code

Policy information about availability of computer code

Data collection	EPU 2.11
Data analysis	Cryo-EM analysis: Warp 1.0.9, Relion 3.1, Relion 5.0, CTFFIND-4.1, ChimeraX 1.10, cryoSPARC 4.4.1, cisTEM 2.0.0 alpha, Isolde 1.10.1, Phenix 1.21.2, Coot 0.9.6<br>Mass spectrometry analysis: MSAmanda v20.0.12368, xiSEARCH v1.8.5, xiFDR v2.3, xiView, XMAS ChimeraX plugin<br>Mass photometry analysis: DiscoverMP<br>Biochemical analysis: GraphPad Prism 10<br>Structure prediction: AlphaFold3

For manuscripts utilizing custom algorithms or software that are central to the research but not yet described in published literature, software must be made available to editors and reviewers. We strongly encourage code deposition in a community repository (e.g. GitHub). See the Nature Portfolio guidelines for submitting code & software for further information.

Data

Policy information about availability of data

All manuscripts must include a data availability statement. This statement should provide the following information, where applicable:

- Accession codes, unique identifiers, or web links for publicly available datasets
- A description of any restrictions on data availability
- For clinical datasets or third party data, please ensure that the statement adheres to our policy

The cryo-EM maps generated in this study were deposited to the EM Data Bank under the accession codes: EMD-55583 (Pol II-Gdown1-RPAP2 composite map), EMD-55578 (Pol II-Gdown1-RPAP2 Pol II core map), EMD-55579 (Pol II-Gdown1-RPAP2 Pol II stalk map), EMD-55580 (Pol II-Gdown1-RPAP2 RPAP2 map), EMD-55581 (Pol II-Gdown1-RPAP2 Gdown1 N-terminus map), EMD-55582 (Pol II-Gdown1-RPAP2 Gdown1 C-terminus map), and EMD-55585 (RPAP2-GPN1-GPN3 map). Model coordinates were deposited to the PDBe under the accession codes: 9T5H (Pol II-Gdown1-RPAP2 complex structure) and 9T5J (GPN1-GPN3-RPAP2 structure). Immunoprecipitation-mass spectrometry and crosslinking-mass spectrometry proteomics data have been deposited to the ProteomeXchange Consortium via the PRIDE partner repository with the dataset identifiers PXD071638 and PXD070852. AlphaFold3 structure predictions have been deposited to the Zenodo repository under the DOI 10.5281/zenodo.20687910. Previously published model coordinates were utilized and are available at the PDBe under the accession codes 8QEP, 9BZ0, and 7B7U. Source Data are provided with this paper.

Research involving human participants, their data, or biological material

Policy information about studies with human participants or human data. See also policy information about sex, gender (identity/presentation), and sexual orientation and race, ethnicity and racism.

Reporting on sex and gender	n/a
Reporting on race, ethnicity, or other socially relevant groupings	n/a
Population characteristics	n/a
Recruitment	n/a
Ethics oversight	n/a

Note that full information on the approval of the study protocol must also be provided in the manuscript.

Field-specific reporting

Please select the one below that is the best fit for your research. If you are not sure, read the appropriate sections before making your selection.

☒ Life sciences ☐ Behavioural & social sciences ☐ Ecological, evolutionary & environmental sciences

For a reference copy of the document with all sections, see nature.com/documents/nr-reporting-summary-flat.pdf

Life sciences study design

All studies must disclose on these points even when the disclosure is negative.

Sample size	No sample size calculation was performed. Biochemical assays and mass-spectrometry of pull-downs from cell extracts were performed in triplicate to allow statistical analysis of the data. The number of collected micrographs was chosen based on expected heterogeneity and flexibility of the analyzed complex and the availability of microscope resources.
Data exclusions	No data were excluded.
Replication	Sample preparation and analysis were reproducible.
Randomization	During 3D refinement, cryo-EM data were split randomly into two halves for gold-standard FSC determination. Biochemical and mass spectrometry experiments were not randomized, but contained appropriate controls.
Blinding	Blinding is not relevant or feasible for these types of experiments. The biochemical and structural experiments presented here are rigorously quality controlled through other criteria and mechanisms as accepted within the field.

Reporting for specific materials, systems and methods

We require information from authors about some types of materials, experimental systems and methods used in many studies. Here, indicate whether each material, system or method listed is relevant to your study. If you are not sure if a list item applies to your research, read the appropriate section before selecting a response.

Materials & experimental systems

n/a	Involved in the study
☐	☒ Antibodies
☐	☒ Eukaryotic cell lines
☒	☐ Palaeontology and archaeology
☐	☒ Animals and other organisms
☒	☐ Clinical data
☒	☐ Dual use research of concern
☒	☐ Plants

Methods

n/a	Involved in the study
☒	☐ ChIP-seq
☒	☐ Flow cytometry
☒	☐ MRI-based neuroimaging

Antibodies

Antibodies used	Purification: 8WG16 (hybridoma line provided by N. Thompson and R. Burgess), anti-GFP nanobody (Addgene plasmid # 49172; http://n2t.net/addgene:49172; RRID:Addgene_49172)<br>Primary [name (supplier name; catalog number; clone name; lot number; dilution)]: anti-GAPDH (Sigma-Aldrich; G8795; GAPDH-71.1; 2219037; 1:5000), anti-U1snRNP 70 (Santa Cruz Biotechnology; sc-9571; C-18; G0816; 1:1000), anti-POLRM2 (Sigma-Aldrich; HPA06814; polyclonal; R101484; 1:1000), anti-GFP (Santa Cruz Biotechnology; sc-9996; B-2; G1819; C1726; 1:1000)<br>Secondary [name (supplier name; catalog number; clone name; lot number; dilution)]: goat-anti-mouse coupled with HRP (abcam; ab5870; polyclonal; 1022871-6; 1:10000), goat-anti-rabbit coupled with HRP(Sigma-Aldrich; A0545; polyclonal; 069M4835V; 1:10000)
Validation	8WG16 antibody was used to purify RNA polymerase II, and anti-GFP nanobody was used to purify GFP-Gdown1. All antibodies are validated by the manufacturer.

Eukaryotic cell lines

Policy information about cell lines and Sex and Gender in Research

Cell line source(s)	Leukemia cell line K562 (source: ATCC, cat. no. CCL-243)
Authentication	The cell line was authenticated by short tandem repeat analysis (see Muhar et al. 2018, Science, DOI: 10.1126/science.aao2793)
Mycoplasma contamination	The cell line was confirmed to be Mycoplasma negative.
Commonly misidentified lines (See ICLAC register)	No commonly misidentified cell lines were used in this study.

Animals and other research organisms

Policy information about studies involving animals; ARRIVE guidelines recommended for reporting animal research, and Sex and Gender in Research

Laboratory animals	This study did not involve laboratory animals.
Wild animals	This study did not involve wild animals.
Reporting on sex	n/a
Field-collected samples	This study did not involve field-collected samples.
Ethics oversight	This project does not raise any ethical concerns. Pig thymus, used for Pol II purification, is sourced from animals approved for food consumption through an officially approved facility in Sieghartskirchen, Lower Austria.

Note that full information on the approval of the study protocol must also be provided in the manuscript.

Plants

Seed stocks	n/a
Novel plant genotypes	n/a
Authentication	n/a
