# Supplementary material for: Structure of cytoplasmic RNA polymerase II
Source: Nat Commun. 2026 Jul 13;17:8640. doi: 10.1038/s41467-026-75416-8 (PMC13486763; doi:10.1038/s41467-026-75416-8)
Supplement: Supplementary file 3 [file 41467_2026_75416_MOESM3_ESM.pdf]

nature portfolio

Peer Review File

Structure of cytoplasmic RNA polymerase II

Corresponding Author: Dr Carrie Bernecky

This file contains all reviewer reports in order by version, followed by all author rebuttals in order by version.

Version 0:

Reviewer comments:

Reviewer #1

(Remarks to the Author)
The manuscript by Hlavata et al. presents an extensive integrative structural, biochemical, and biophysical analysis of a cytoplasmic human RNA polymerase II (Pol II) assembly intermediate involving Gdown1, RPAP2, and the small GTPase heterodimer GPN1–GPN3. The authors combine endogenous and recombinant protein purification, cryo-EM, and crosslinking mass spectrometry to derive atomic models of Pol II–Gdown1–RPAP2 and the RPAP2–GPN1–GPN3 subcomplex. Based on these data as well as mass photometry and pull-down experiments, the authors outline proposed roles of RPAP2 and GPN1-GPN3 in cytoplasmic RNA Pol II assembly, and they identify multiple sterical incompatibilities between Pol II-bound Gdown1 and transcription factors, thereby explaining the mechanism of previously observed cellular roles of Gdown1.

The cryo-EM structures of Pol II–Gdown1–RPAP2 and RPAP2–GPN1–GPN3 are convincing and well supported by XL-MS. The endogenous purification strategy is a clear strength and increases the confidence that the observed complexes represent physiological intermediates. Furthermore, structure determination of a complex including GPN1 and GPN3 - which were not visualised in the context of the Pol II assembly intermediate, presumably due to disorder - is a welcome extension of the authors' analysis.

The proposed mechanisms rely heavily on existing literature data and, in some instances, AlphaFold modelling but are not directly tested in cellular assays. This approach is entirely appropriate to support most of the authors' conclusions; however, the discussion of the function of GPN1-GPN3 as a "critical molecular switch" (line 416 and to a lesser degree line 92) is somewhat speculative and the authors should consider toning down these statements in order to avoid overstating their extended Pol II biogenesis model, given the available evidence.

The manuscript is generally well written, though there are some redunancies, and some sections might benefit from another editing/re-writing pass to increase clarity.

We have compiled a list of specific points that the authors should address when revising their manuscript. If suitably revised, the manuscript will be a strong candidate for publication in Nature Communications.

Specific points:

Abstract:

"... reveal a GTP-controlled switch during the final stages of Pol II biogenesis..." - given that it is not entirely clear what this switch controls and how it works (and given that the part that is understood is captured in a previous sentence), the authors should consider removing this clause from the abstract.

Introduction:

Line 79: "except for during transcriptionally silent developmental stages": "for" is probably redundant.

Results:

Line 127 is redundant with the beginning of the subsequent section.

Line 129: In this section, the fact that GPN1-GPN3 were present in the biochemically purified complex but could not be observed in the cryo-EM density could be clearly stated earlier than is currently the case (e.g. in an additional sentence after the "summary" sentence in line 139).

Line 141: "However, due to improved density, and a previously existing model for Pol II-Gdown1 (ref. 15) could be generated...": Meaning unclear. This should probably be rephrased. Additionally, after reading the remainder of this sentence and the following sentence, it is unclear if the authors' new model is consistent with the previous model (ref. 15) or not.

Line 149: NES is spelled out here but abbreviated in the introduction.

Line 158: "Although few inter-subunit crosslinks were observed, all were consistent with the Pol II-Gdown1-RPAP2 structure...": Considering the small number of crosslinks observed, it would be feasible (and useful) to add a figure panel in which these are explicitly mapped onto the model.

Line 215: "Additionally, previous work has indicated a direct interaction between GPN1 and both Pol II and RPAP2 to GPN1": Meaning unclear (final "to GPN1" redundant?).

Line 222: "We assessed complex formation by mass photometry and size-exclusion chromatography, both of which indicated assembly of the Pol II–Gdown1–RPAP2–GPN1–GPN3 complex": The display of a single gel filtration fraction (?) as an indication of complex formation is not convincing evidence (Fig. 3a). The mass photometry data appear to provide much stronger evidence (Fig. 3b). It may be best to reword this sentence. The mass photometry data are indicative while the size exclusion fraction is merely consistent. Otherwise, a suitable extended portion of the gel filtration run should be shown to demonstrate co-elution as a single peak.

Line 254: "Structure prediction of Pol II-GPN1-GPN3 suggested that GPN1-GPN3 interacts with the DNA-binding cleft of Pol II through electrostatic interactions with negatively charged regions of GPN1 and especially GPN3, with the negatively charged GPN3 C-terminal tail predicted to associate with the pore and tunnel which accommodate backtracked RNA in the arrested elongation complex35 (Fig. 3d, Extended Data Fig. 4g,h).": This sentence is very long, difficult to understand, and should be re-written.

Furthermore, the authors should display the PAE plot in addition to the pLDDT scores (shown in Extended Data Fig. 4g) for the Pol II-GPN1-GPN3 structure prediction. The pLDDT metric is a local metric that is useful to assess whether the folding of the individual domains is reliable, but it does not inform on whether a protein-protein interaction (or the relative positioning of proteins or protein domains) is accurate.

Line 283: "GPN1-GPN3 formed the core of the structure, with the very C-terminal ends of both flexible and solvent-exposed...": This is difficult to understand. Maybe rephrase, or change to "... both proteins flexible...".

Line 285: The description of these interactions should be complemented with a visualisation; probably, just labelling some of the segments in question in Fig. 5d, e would help already.

Line 308: The sentence starting in line 311 should probably immediately follow this sentence, and it should be clearly stated that the GTP conformation is predicted to lead to a steric clash. Otherwise, these sentences appear to be unnecessarily vague.

Line 319/320: "non-hydrolyzable GTP" should be "non-hydrolysable GTP analogue".

Line 423: "guanine exchange factors" should be "guanine nucleotide exchange factors".

Methods:

Line 781/782: Map anisotropy is mentioned. The authors should provide a metric for map anisotropy (e.g. as derived from the 3D FSC validation server) among their validation statistics and should show the output from such a program in an Extended Data/Supplementary Figure.

Line 796: "A DeepEMhancer-postprocessed map was used for display only": Can the authors confirm that:
- a conventionally post-processed map was deposited as the main map to the EMDB, not the DeepEMhancer map?
- all model refinement and all validation (e.g. model vs. map FSC) was performed using a conventionally post-processed map?
If not, the sentence cited above should be re-worded or the data analysis modified. In this case, the authors should consider that using DeepEMhancer-treated maps for model refinement and validation is currently not a generally accepted practice in the field.

Figures:

Fig. 5f should be promoted to have its own main text figure (i.e. Fig. 6). As-is, it is unnecessarily compressed and difficult to see. The figure legend for this panel should then be expanded from its current form.

Reviewer #2

(Remarks to the Author)
I co-reviewed this manuscript with one of the reviewers who provided the listed reports. This is part of the Nature Communications initiative to facilitate training in peer review and to provide appropriate recognition for Early Career Researchers who co-review manuscripts.

Reviewer #3

(Remarks to the Author)
"Structure of Cytoplasmic RNA Polymerase II"
In this work, the authors employ an integrative structural biology approach, incorporating CryoEM, alphafold and crosslinking mass-spectrometry analysis together with biophysical characterization to investigate the structure of the RNA Pol II-Gdown1-RPAP2-GPN1/3 complex, representing a late-stage polymerase biogenesis intermediate. The authors build on this characterization through separate structural analysis of the RPAP2-GPN1/3 complex, identifying a GDP-dependency in the interaction between RPAP2 and GPN1/3, giving insight into the possible mechanism of polymerase complex assembly. This article is clear, concise and well-written, covering an important yet under-investigated aspect of polymerase biology. We would recommend this manuscript for publication after addressing the following issues:

1. Has the tagged Gdown1 cell line been published previously? Could the authors include a validation of the endogenous tagging?

2. The authors mention that there are many significantly enriched proteins from the AP-MS experiment. Could the authors clarify the level of confidence and fold enrichment at which proteins were considered significantly enriched? Also, the volcano does not give any indication about the stoichiometry of the factors compared to Pol II, which may be interesting to show in light of the following work and to give, at least indicative information about Pol II-bound and Pol II-unbound fractions in the cytoplasm.

3. In this paper, the authors extensively employ alphafold to model the potential complex formed between the polymerase and GPN1/3 complex and TTF2. However, aside from pLDDT scores, no other confidence metrics are included. Could the authors include PAE plots, iPTM and PTM scores for each prediction. Without these it is not possible to assess accurately the quality of the prediction in each instance.

4. In figure 3a, the molecular weight ladder should be labelled on the coomassie stained gel.

5. While investigating the GPN1/3-Pol II interaction, the authors collect XL-MS data which suggested, together with the mass photometry data, the presence of an interaction. This is then followed by an alphafold prediction of GPN1/3 binding to the pore and the cleft of the polymerase, consistent with the author's data showing a polymerase conformational requirement for this interaction. However, the XL-MS data presents a great opportunity to validate the alphafold prediction, could the authors map the identified crosslinks onto the prediction to determine if there is agreement between the data, including Ca-Ca distance cutoff violation to probe further if the binding mode predicted is supported by the biophysical data.

6. For the RPAP2-GPN1/3 complex, in extended data figure 5, please include a histogram showing the range of Ca-Ca crosslinks observed for the complex to aid in the assessment of the violation rate between the crosslinks and the solved structure. This would be required to state that there is good agreement between the structure and XL-MS data.

7. Please include a viewing angle distribution for the cryoEM data to assess map anisotropy.

8. In extended data figures 4a, 4b and 5a, please include molecular weight ladders.

Reviewer #4

(Remarks to the Author)
This manuscript by Hlavata et al describes a combination of biochemistry and cryo-EM to study the cytoplasmic form of RNA polymerase II (PolII) prior to complete assembly and import into the nucleus. The authors fractionated lysate from a human cell line to isolate a cytoplasmic complex using an affinity handle on Gdown1, which binds PolII. In addition to PolII, further proteins could be purified, which have been implicated in PolII assembly (RPAP2, and the GPN1-GPN3 heterodimer). The manuscript comes to similar conclusions as a recent bioRxiv pre-print from Patrick Cramer and Christian Dienemann, which is reassuring. However, I'd like to emphasize that the present manuscript is a much more thorough piece of work. I am in favor of publication but would encourage the authors to consider my comments below.

I understand that the following comment is not very useful (because it is not very constructive) but I found the introduction difficult to follow. I think it would be very useful if it is more accessible to a broader readership.

Can the clamp in the reconstruction of endogenous PolII-RPAP2-Gdown1 be seen in low-pass filtered maps? I understand that this is not the only human PolII reconstruction that “suffers” from this but I find this very curious. Could it be oscillating or is it disordered? Have the authors tried focused 3D classification? I’m sure they have but it would be useful to comment on this in the paper. I find it remarkable that it is so disordered and that we have almost no signal for it. It is consistent with the recent pre-print from the Cramer lab and reminiscent of the observation by the Roeder lab (Jishage et al., NSMB 2019) – although in the latter case nuclear (and thus assembled) PolII was used as far as I know?!

Line 222-240: I assume the authors can support the interpretation from the mass-photometry in Fig. 3b with SEC experiments and SDS-PAGE of the peak fractions. If so, it needs to be shown. If feasible, SDS PAGE should show individual peak fractions so we can appreciate if the composition changes as a function of position along the peaks. In Fig. 3a RPAP2 and GPN1-GPN3 appear sub-stoichiometric – please comment in manuscript? The turquois trace in Fig. 3b shows three (deconvoluted) peaks – is this a mistake or was the mass-photometry data deconvoluted – if so, please state in legend. It would also be useful to label the peaks (e.g. with roman numerals) and write in the figure or legend what each peak likely corresponds to.

Line 238: Fig. 3b instead of 2b?

Line 238-line 240: The statement that the experiment in Fig. 3b proofs that PolII in complex with RPAP2-GPN1-GPN3 is monomeric in the cytoplasm is only true if we assume that addition of Gdown1-RPAP2-GPN1-GPN3 to mature PolII (presumably purified porcine PolII was the source for this experiment) mimics the cytoplasmic complex prior to import – do we know that? Please comment on that in the text or mention as a potential caveat and/or limitation.

Line 253-269: AF3 presumably predicts PolII with an ordered and closed clamp domain – the authors should comment on this since they then argue that an open clamp is likely required for binding and they do the biochemical experiments described in lines 261-269 to support this claim.

Line 242-269: Along the same lines, it would be important to state more clearly what the experimental proof is for assuming GPN1-GPN3 on its own binds the PolII cleft (aside from an AF3 prediction). The crosslinking mass spectrometry (Extended Fig. 4f) shows cross-links to the N-terminus and the disordered CTD - not necessarily the clamp, correct? Also, why was the AF3 prediction done with PolII and GPN1-GPN3, when the mass spectrometry was done with PolII-Gdown1-RPAP2-GPN1-GPN3? What does the AF3 prediction look like for the whole complex?

Extended Fig. 4f: Are the crosslinks between GPN1-GPN3 and RPB1 on the one hand and with RPAP2 on the other hand compatible? In other words, does the crosslinking data support two alternative binding modes of GPN1-GPN3 (to the cleft and to the RPAP2 CTD) or could crosslinks to the clamp and RPAP2 be satisfied at the same time?

Paragraph starting at line 271 and Fig. 3: The authors have presumably aimed to obtain a cryo-EM reconstruction of the PolII-Gdown1-RPAP2-GPN1-GPN3 and/or PolII-RPAP2-GPN1-GPN3 complex using the recombinant proteins and porcine PolII, correct? Please comment on why this may have failed or, alternatively, why there was no attempt to do that? Do we know if binding of all or a subset of these factors can "reverse" PolII assembly (see also one of the previous comments)? Have they considered adding recombinant RPAP2, GPN1-GPN3 to the endogenous cytoplasmic PolII complex similar to what had been described in the Cramer lab pre-print? If not, why not – please comment on that in the manuscript?

Line 301-325 and Fig.5: Why is the peak for the complex higher in presence of GDPNP compared to GTP – is there some binding issue of the analogue to the complex and we see some apo form? Also, apo and GDP are identical – are we sure GDP binds in the experimental conditions used here?

Line 335: Please be more specific – what about Gdown1 is revised compared to previous work?

Line 445: the reference is missing

Line 570: Can you see particles at a defocus of -0.3? How reliable is picking at such a low defocus value?

Version 1:

Reviewer comments:

Reviewer #1

(Remarks to the Author)
The authors have addressed our comments and concerns. We recommend publication of the manuscript in Nature Communications.

That said, we would encourage the authors to try to find alternative wording for "... reveal a molecular switch governing biogenesis factor association..." in the abstract to more accurately reflect the experimental findings reported in the paper. However, in order to avoid undue delays to the publication process, this should be agreed between the authors and the editor and will not require further external review.

Reviewer #2

(Remarks to the Author)
I co-reviewed this manuscript with one of the reviewers who provided the listed reports. This is part of the Nature Communications initiative to facilitate training in peer review and to provide appropriate recognition for Early Career Researchers who co-review manuscripts.

Reviewer #3

(Remarks to the Author)
The authors have clarified the few issues/remarks and the manuscript can be accepted in its current form.

Reviewer #4

(Remarks to the Author)
Thank for sending the revised manuscript. The authors have addressed all my concerns and I support publication.

Open Access This Peer Review File is licensed under a Creative Commons Attribution 4.0 International License, which permits use, sharing, adaptation, distribution and reproduction in any medium or format, as long as you give appropriate credit to the original author(s) and the source, provide a link to the Creative Commons license, and indicate if changes were made.
In cases where reviewers are anonymous, credit should be given to 'Anonymous Referee' and the source.
The images or other third party material in this Peer Review File are included in the article’s Creative Commons license, unless indicated otherwise in a credit line to the material. If material is not included in the article’s Creative Commons license and your intended use is not permitted by statutory regulation or exceeds the permitted use, you will need to obtain permission directly from the copyright holder.
To view a copy of this license, visit https://creativecommons.org/licenses/by/4.0/

List of responses to reviewer comments

"Structure of cytoplasmic RNA polymerase II"
(Annamaria Hlavata, Benjamin Neuditschko, Ulla Schellhaas, Clemens Plaschka, Franz Herzog, and Carrie Bernecky)

Nature Communications manuscript NCOMMS-25-94449-T

Responses are in dark blue *italics*. Text changes are indicated in red.

REVIEWER COMMENTS

Reviewer #1 (Remarks to the Author):

The manuscript by Hlavata et al. presents an extensive integrative structural, biochemical, and biophysical analysis of a cytoplasmic human RNA polymerase II (Pol II) assembly intermediate involving Gdown1, RPAP2, and the small GTPase heterodimer GPN1–GPN3. The authors combine endogenous and recombinant protein purification, cryo-EM, and crosslinking mass spectrometry to derive atomic models of Pol II–Gdown1–RPAP2 and the RPAP2–GPN1–GPN3 subcomplex. Based on these data as well as mass photometry and pull-down experiments, the authors outline proposed roles of RPAP2 and GPN1-GPN3 in cytoplasmic RNA Pol II assembly, and they identify multiple sterical incompatibilities between Pol II-bound Gdown1 and transcription factors, thereby explaining the mechanism of previously observed cellular roles of Gdown1.

The cryo-EM structures of Pol II–Gdown1–RPAP2 and RPAP2–GPN1–GPN3 are convincing and well supported by XL-MS. The endogenous purification strategy is a clear strength and increases the confidence that the observed complexes represent physiological intermediates. Furthermore, structure determination of a complex including GPN1 and GPN3 - which were not visualised in the context of the Pol II assembly intermediate, presumably due to disorder - is a welcome extension of the authors' analysis.

We thank the reviewer, and very much appreciate the positive feedback.

The proposed mechanisms rely heavily on existing literature data and, in some instances, AlphaFold modelling but are not directly tested in cellular assays. This approach is entirely appropriate to support most of the authors' conclusions; however, the discussion of the function of GPN1-GPN3 as a "critical molecular switch" (line 416 and to a lesser degree line 92) is somewhat speculative and the authors should consider toning down these statements in order to avoid overstating their extended Pol II biogenesis model, given the available evidence.

We have edited the discussion text around line 416 to clarify, as we indeed aim to speculate on potential functional roles of GPN1-GPN3 in the context of Pol II assembly. The section title now reads: "Putative expanded model of Pol II biogenesis"

And the sentence around line 416 now reads:
"Based on these insights, we speculate that GPN1-GPN3 may function in Pol II biogenesis as a marker and molecular switch facilitating the assembly process."

The manuscript is generally well written, though there are some redunancies, and some sections might benefit from another editing/re-writing pass to increase clarity.

We have edited the text, particularly the introduction and description of the Pol II-GPN1-GPN3 structure prediction, and hope that this has improved the clarity.

We have compiled a list of specific points that the authors should address when revising their manuscript. If suitably revised, the manuscript will be a strong candidate for publication in Nature Communications.

Specific points:

Abstract:

"... reveal a GTP-controlled switch during the final stages of Pol II biogenesis..." - given that it is not entirely clear what this switch controls and how it works (and given that the part that is understood is captured in a previous sentence), the authors should consider removing this clause from the abstract.

We have rephrased the final sentence of the abstract to the following:
"The combined results uncover a network of interactions that chaperone cytoplasmic Pol II to prevent aberrant interactions, reveal a molecular switch governing biogenesis factor association, and suggest a general mechanism for the action of GPN-loop GTPase family of enzymes."

Introduction:

Line 79: "except for during transcriptionally silent developmental stages": "for" is probably redundant.

We have edited the text as suggested. It now reads:
"...which keeps Gdown1 predominantly localized to the cytoplasm except during transcriptionally silent developmental stages..."

Results:

Line 127 is redundant with the beginning of the subsequent section.

We have moved the first sentence of the subsequent section up, replacing line 127 to avoid this redundancy.

Line 129: In this section, the fact that GPN1-GPN3 were present in the biochemically purified complex but could not be observed in the cryo-EM density could be clearly stated earlier than is currently the case (e.g. in an additional sentence after the "summary" sentence in line 139).

We have rephrased the text as follows:
"Cryo-EM analysis of the resulting complexes revealed the structure of Pol II bound to Gdown1 and RPAP2 at a nominal resolution of 2.7 Å. In the reconstruction, 12-subunit Pol II, the Pol II-binding region of RPAP2, and three distinct Pol II-binding regions of Gdown1, but no GPN1-GPN3 density, could be resolved. As expected for an RPAP2-bound Pol II complex, no nucleic acid density was visible."

Line 141: "However, due to improved density, and a previously existing model for Pol II-Gdown1 (ref. 15) could be generated...": Meaning unclear. This should probably be rephrased. Additionally, after reading the remainder of this sentence and the following sentence, it is unclear if the authors' new model is consistent with the previous model (ref. 15) or not.

The previous model for the Gdown1 protrusion-binding domain is consistent with our revised model, but a larger region of sequence could be modeled in the current work. The rest was significantly changed – two additional Gdown1 Pol II-binding domains were previously modeled as a smaller, continuous peptide with no sequence register assigned.

We have rephrased this portion of the text to clarify:
"Due to improved density, a previously existing model for Pol II-Gdown1 could be revised. In the updated model, the N-terminal Pol II-binding regions of Gdown1 were revealed to be discontinuous, and the sequence register of both regions of Gdown1 could be assigned. Furthermore, the model for the Gdown1 region binding to the RPB2 protrusion could be extended"

Line 149: NES is spelled out here but abbreviated in the introduction.

We have replaced "nuclear export sequence" and "cytoplasmic anchoring sequence" with the abbreviations NES and CAS as defined in the introduction.

Line 158: "Although few inter-subunit crosslinks were observed, all were consistent with the Pol II-Gdown1-RPAP2 structure...": Considering the small number of crosslinks observed, it would be feasible (and useful) to add a figure panel in which these are explicitly mapped onto the model.

We have expanded Supplementary Fig. 3 to show the mapped crosslinks.

Line 215: "Additionally, previous work has indicated a direct interaction between GPN1 and both Pol II and RPAP2 to GPN1": Meaning unclear (final "to GPN1" redundant?).

Yes – thank you, we have deleted the unintended extra "to GPN1".

Line 222: "We assessed complex formation by mass photometry and size-exclusion chromatography, both of which indicated assembly of the Pol II–Gdown1–RPAP2–GPN1–GPN3 complex": The display of a single gel filtration fraction (?) as an indication of complex formation is not convincing evidence (Fig. 3a). The mass photometry data appear to provide much stronger evidence (Fig. 3b). It may be best to reword this sentence. The mass photometry data are indicative while the size exclusion fraction is merely consistent. Otherwise, a suitable extended portion of the gel filtration run should be shown to demonstrate co-elution as a single peak.

We have now included a size exclusion chromatogram for Pol II–Gdown1–RPAP2–GPN1–GPN3, with SDS-PAGE analysis of each peak and mass photometry measurements of a fraction containing the Pol II–Gdown1–RPAP2–GPN1–GPN3 complex (Extended Data Fig. 4).

Line 254: "Structure prediction of Pol II-GPN1-GPN3 suggested that GPN1-GPN3 interacts with the DNA-binding cleft of Pol II through electrostatic interactions with negatively charged regions of GPN1 and especially GPN3, with the negatively charged GPN3 C-terminal tail predicted to associate with the pore and tunnel which accommodate backtracked RNA in the arrested

elongation complex35 (Fig. 3d, Extended Data Fig. 4g,h).": This sentence is very long, difficult to understand, and should be re-written.

We have rephrased this section for clarity, and to add additional information about the clashes observed when Pol II containing the clamp domain is used for structure prediction. The text now reads:
"Structure prediction of Pol II-GPN1-GPN3 lacking the clamp domain, which was unresolved and presumably mobile in the Pol II-Gdown1-RPAP2 structure, yielded a higher-confidence structure without clashes (Supplementary Fig. 4j,k). Analysis of this model suggested that GPN1-GPN3 interacts with the DNA-binding cleft of Pol II through electrostatic interactions mediated by negatively charged regions of GPN1 and GPN3. Extensive interactions were observed between the negatively charged GPN3 C-terminal tail and the Pol II pore and funnel domains, which accommodate backtracked RNA in the arrested elongation complex(Fig. 3d, Supplementary Fig. 4l)."

Furthermore, the authors should display the PAE plot in addition to the pLDDT scores (shown in Extended Data Fig. 4g) for the Pol II-GPN1-GPN3 structure prediction. The pLDDT metric is a local metric that is useful to assess whether the folding of the individual domains is reliable, but it does not inform on whether a protein-protein interaction (or the relative positioning of proteins or protein domains) is accurate.

We have now added PAE plots for all predicted structures to Supplementary Figures 3, 4 and 5.

Line 283: "GPN1-GPN3 formed the core of the structure, with the very C-terminal ends of both flexible and solvent-exposed...": This is difficult to understand. Maybe rephrase, or change to "... both proteins flexible...".

We have rephrased this section to read:
"The majority of GPN1 and GPN3 were well-resolved, except for the very C-terminal ends of both, which were flexible and solvent-exposed. This flexible region includes the GPN1 NES."

Line 285: The description of these interactions should be complemented with a visualisation; probably, just labelling some of the segments in question in Fig. 5d, e would help already.

We have labeled the RPAP2 segments and the last resolved amino acids of GPN1 and GPN3 in Fig. 4e.

Line 308: The sentence starting in line 311 should probably immediately follow this sentence, and it should be clearly stated that the GTP conformation is predicted to lead to a steric clash. Otherwise, these sentences appear to be unnecessarily vague.

We have reorganized the text to discuss the known GTP-dependent conformational changes earlier. The section now reads:
"This observation coupled to the conservation of the GTPase G1-G3 and GPN motifs indicates that in the context of a GPN1-GPN3 heterodimer, both GPN1 and GPN3 are capable of GTP hydrolysis. In the presence of GTP or its non-hydrolyzable analogue, the GPN loop is involved in the coordination of the γ-phosphate, leading to a more closed dimeric arrangement. In the RPAP2-GPN1-GPN3 structure, the GPN-loops of both GPN1 and GPN3 were positioned away from the bound nucleotide, consistent with a GDP-bound state (Supplementary Fig. 5p). RPAP2 passed directly next to the GDP molecules of both GPN1 and GPN3, with a mode of interaction incompatible with the closed, GTP-bound state (Fig. 5a-c)."

Line 319/320: "non-hydrolyzable GTP" should be "non-hydrolysable GTP analogue".

We have edited this to "GppNHp" for clarity.

Line 423: "guanine exchange factors" should be "guanine nucleotide exchange factors".

We have edited the text accordingly.

Methods:

Line 781/782: Map anisotropy is mentioned. The authors should provide a metric for map anisotropy (e.g. as derived from the 3D FSC validation server) among their validation statistics and should show the output from such a program in an Extended Data/Supplementary Figure.

Extended Data Fig. 5 now includes the histogram and directional FSC plot from the 3D FSC validation server for both the final RPAP2-GPN1-GPN3 reconstruction and the intermediate cryosparc 3D reconstruction. We also have included angular distribution plots for all 3D reconstructions in the manuscript.

Line 796: "A DeepEMhancer-postprocessed map was used for display only": Can the authors confirm that:

- a conventionally post-processed map was deposited as the main map to the EMDB, not the DeepEMhancer map?

Yes – the current version of the entry includes the b-factor sharpened map (b-factor of -50) as the main map. The DeepEMhancer map and unsharpened map are deposited as additional densities. The half maps and post-processing mask are also available.

- all model refinement and all validation (e.g. model vs. map FSC) was performed using a conventionally post-processed map?

Yes, this is the case - model refinement and validation were carried out using the conventionally post-processed map (sharpened with a b-factor of -50).

If not, the sentence cited above should be re-worded or the data analysis modified. In this case, the authors should consider that using DeepEMhancer-treated maps for model refinement and validation is currently not a generally accepted practice in the field.

We completely agree with the reviewer.

Figures:

Fig. 5f should be promoted to have its own main text figure (i.e. Fig. 6). As-is, it is unnecessarily compressed and difficult to see. The figure legend for this panel should then be expanded from its current form.

We have moved the schematic to a new Fig. 6 as suggested.

Reviewer #2 (Remarks to the Author):

I co-reviewed this manuscript with one of the reviewers who provided the listed reports. This is part of the Nature Communications initiative to facilitate training in peer review and to provide appropriate recognition for Early Career Researchers who co-review manuscripts.

We thank the reviewer for their effort in reading and commenting on our manuscript.

Reviewer #3 (Remarks to the Author):

“Structure of Cytoplasmic RNA Polymerase II”

In this work, the authors employ an integrative structural biology approach, incorporating CryoEM, alphafold and crosslinking mass-spectrometry analysis together with biophysical characterization to investigate the structure of the RNA Pol II-Gdown1-RPAP2-GPN1/3 complex, representing a late-stage polymerase biogenesis intermediate. The authors build on this characterization through separate structural analysis of the RPAP2-GPN1/3 complex, identifying a GDP-dependency in the interaction between RPAP2 and GPN1/3, giving insight into the possible mechanism of polymerase complex assembly. This article is clear, concise and well-written, covering an important yet under-investigated aspect of polymerase biology. We would recommend this manuscript for publication after addressing the following issues:

We thank the reviewer for their constructive comments.

1. Has the tagged Gdown1 cell line been published previously? Could the authors include a validation of the endogenous tagging?

No, this is a new cell line. We have added Sanger sequencing and western blot validation to Extended Data Fig. 1b and c.

2. The authors mention that there are many significantly enriched proteins from the AP-MS experiment. Could the authors clarify the level of confidence and fold enrichment at which proteins were considered significantly enriched? Also, the volcano does not give any indication about the stoichiometry of the factors compared to Pol II, which may be interesting to show in light of the following work and to give, at least indicative information about Pol II-bound and Pol II-unbound fractions in the cytoplasm.

We considered a P-value of less than 0.05 significant. We now mention this in the text description and the Figure 1 legend. The zoom-in views show proteins with adjusted P-value of less than 0.0001, and now include this value in the legend as well. We did not set any cutoffs based on fold enrichment, which was, however, correlated with the calculated P-value.

In Revision Figure 1 below, we show a heat map and bar plot of the mass spectrometry normalized areas for the subunits of Pol II, RPAPs, and GPN proteins. The normalized areas of RPAP2 and GPN1 are higher than multiple Pol II subunits, suggesting they these proteins are present at roughly comparable levels to Pol II. However, because of the semi-quantitative nature of this mass spectrometry experiment (performed without spike-in normalization), we do not feel confident using the normalized area to estimate stoichiometry. We can, however, add this data to Supplementary Fig. 1 if the reviewer strongly prefers.

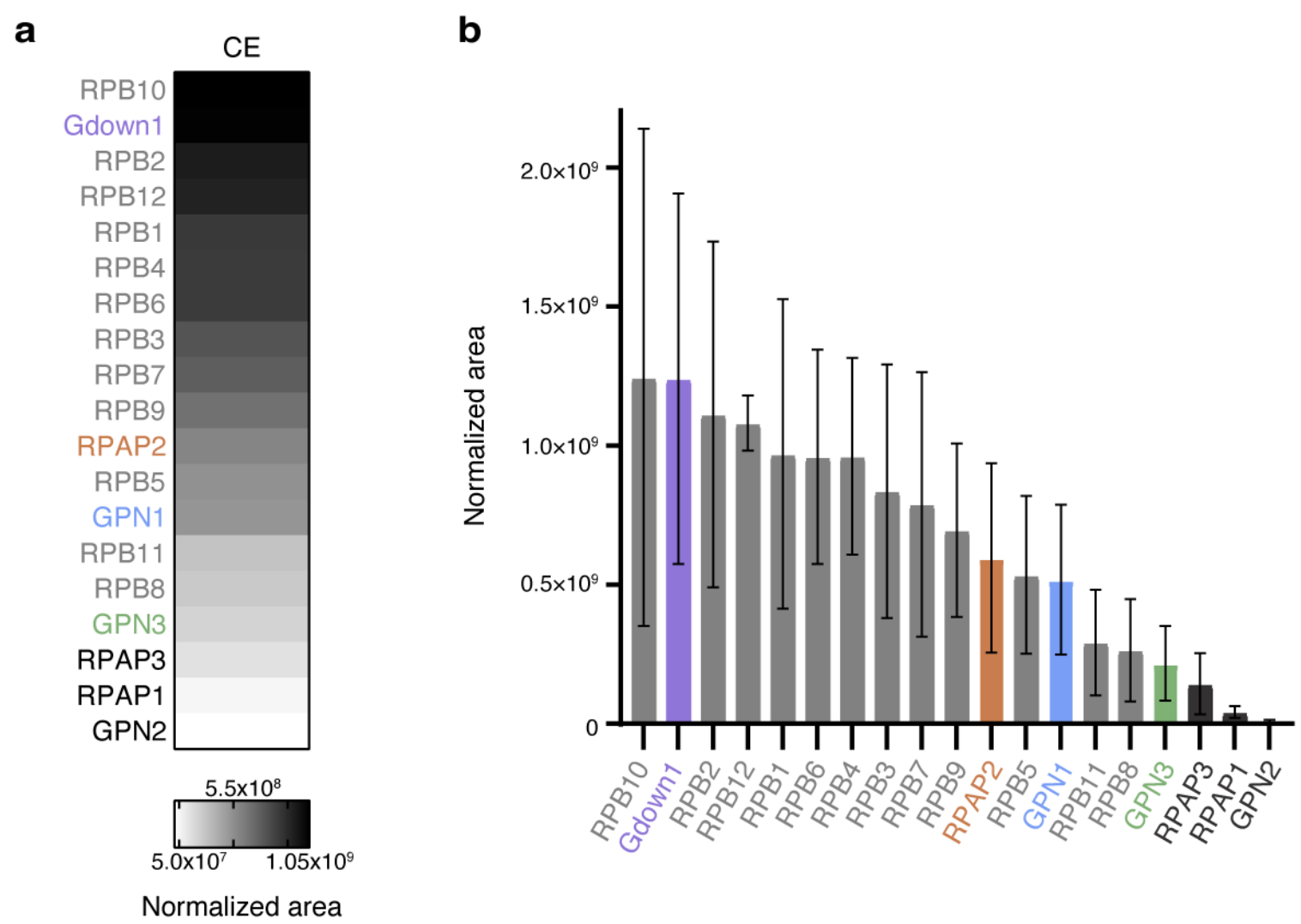

***Revision Figure 1**. Semi-quantification of the relative abundance of Pol II subunits and biogenesis factors. **a**, Heatmap indicating the abundance of cytoplasmic Gdown1-associating, Pol II biogenesis-related proteins. Intensity-Based Absolute Quantitation (iBAQ) was applied for protein area quantification. **b**, Bar graph showing normalized abundance +/- standard deviation for proteins in (a). Proteins are ordered by average normalized area.*

3. In this paper, the authors extensively employ alphafold to model the potential complex formed between the polymerase and GPN1/3 complex and TTF2. However, aside from pLDDT scores, no other confidence metrics are included. Could the authors include PAE plots, iPTM and PTM scores for each prediction. Without these it is not possible to assess accurately the quality of the prediction in each instance.

We have now added PAE plots for all predicted structures (Supplementary Figures 3, 4, and 5), and PTM and iPTM scores to the respective figure legends.

4. In figure 3a, the molecular weight ladder should be labelled on the coomassie stained gel.

The marker lane has been added to Fig. 3a and Fig. 4b.

5. While investigating the GPN1/3-Pol II interaction, the authors collect XL-MS data which suggested, together with the mass photometry data, the presence of an interaction. This is then followed by an alphafold prediction of GPN1/3 binding to the pore and the cleft of the polymerase, consistent with the author's data showing a polymerase conformational requirement for this interaction. However, the XL-MS data presents a great opportunity to validate the alphafold prediction, could the authors map the identified crosslinks onto the prediction to determine if there is agreement between the data, including Ca-Ca distance cutoff violation to probe further if the binding mode predicted is supported by the biophysical data.

The Pol II-GPN1-3 AphaFold prediction in the original version of the manuscript included a version of Pol II lacking the clamp domain, as this produced a more reliable prediction for the complex (see updated Supplementary Fig. 4) without the structural clashes observed when predicting a clamp-containing complex. To accommodate GPN1-GPN3 in the DNA-binding cleft, the Pol II clamp would need to adopt a different conformation. We apologize that this was not adequately described in the submitted manuscript, and have now substantially rephrased this section in the text.

We have mapped the crosslinks of GPN1-GPN3 with Pol II into a predicted structure of Pol II-GPN1-GPN3 (including the Pol II clamp domain) (Revision Figure 2). The XL-MS data include two crosslinks between the clamp domain and GPN1, with Ca-Ca distances are 40.75 Å and

61.2 Å. However, both of the crosslinks occur between the Pol II clamp, which appears flexible in our complex, and a helix of GPN1 that, although confidently predicted (pLDDT >70), is unresolved in our cryo-EM structure and also likely flexible/unstructured. Thus, although the XL-MS data do show clamp-GPN1 crosslinks, due to this flexibility they cannot be used for validation.

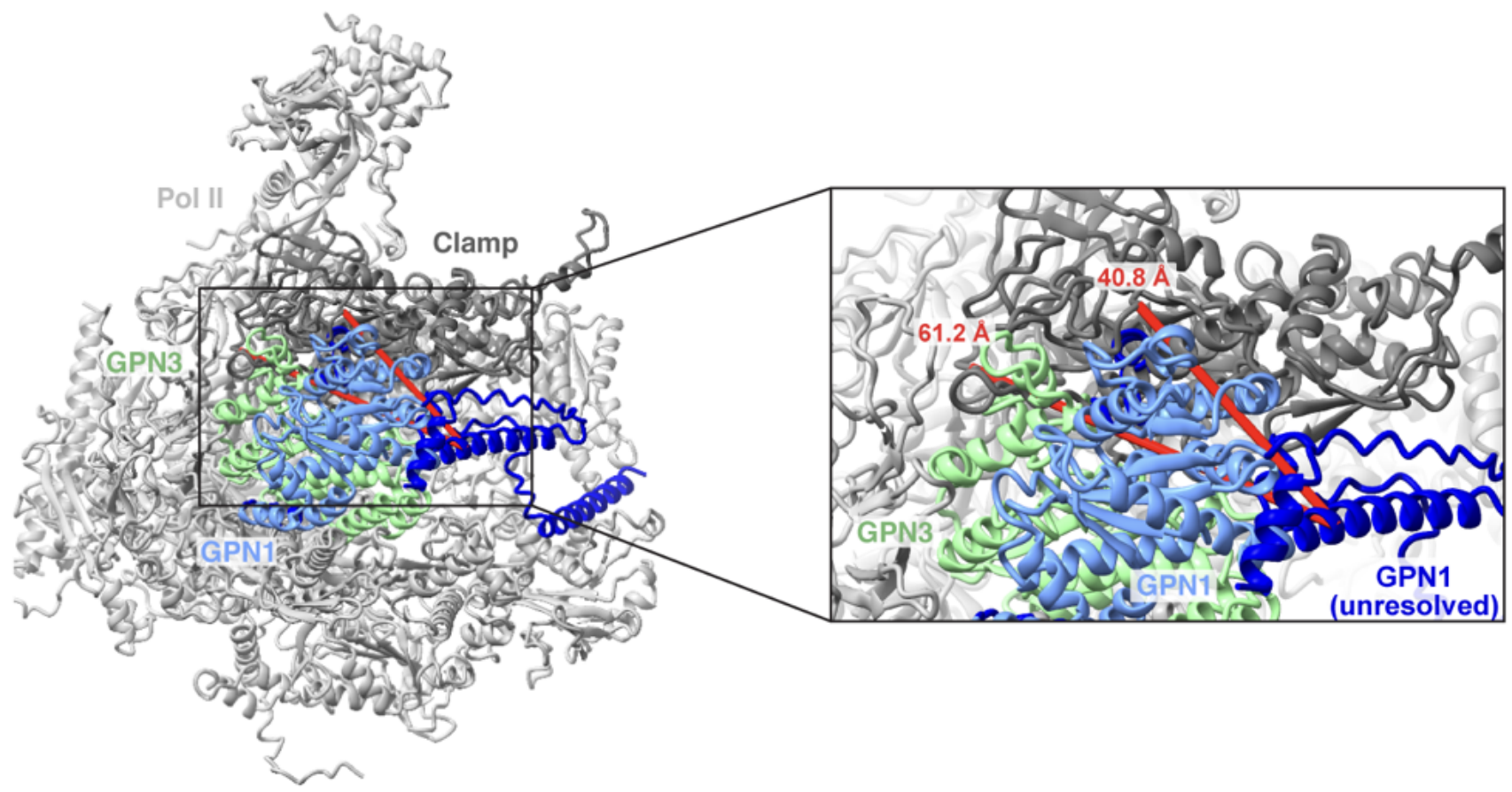

***Revision Figure 2**. Clamp-GPN1 crosslinks mapped onto the Pol II-GPN1-GPN3 structure prediction. The AlphaFold 3 prediction of Pol II containing the clamp domain bound to GPN1 and GPN3 (left), with a zoom-in view shown on the right. Crosslinks between the clamp domain and a region of GPN1 unresolved in our cryo-EM reconstruction (dark blue) are shown in red.*

6. For the RPAP2-GPN1/3 complex, in extended data figure 5, please include a histogram showing the range of Ca-Ca crosslinks observed for the complex to aid in the assessment of the violation rate between the crosslinks and the solved structure. This would be required to state that there is good agreement between the structure and XL-MS data.

A histogram of the Ca-Ca distances for the RPAP2-GPN1-GPN3 complex has been added to Extended Data Fig. 5.

7. Please include a viewing angle distribution for the cryoEM data to assess map anisotropy.

We have added angular distribution plots for the cryo-EM data to Supplementary Figs. 2 and 5.

8. In extended data figures 4a, 4b and 5a, please include molecular weight ladders.

We have added the molecular weight markers as requested.

Reviewer #4 (Remarks to the Author):

This manuscript by Hlavata et al describes a combination of biochemistry and cryo-EM to study the cytoplasmic form of RNA polymerase II (PolII) prior to complete assembly and import into the nucleus. The authors fractionated lysate from a human cell line to isolate a cytoplasmic complex using an affinity handle on Gdown1, which binds PolII. In addition to PolII, further proteins could be purified, which have been implicated in PolII assembly (RPAP2, and the GPN1-GPN3 heterodimer). The manuscript comes to similar conclusions as a recent bioRxiv pre-print from Patrick Cramer and Christian Dienemann, which is reassuring. However, I'd like to emphasize that the present manuscript is a much more thorough piece of work. I am in favor of publication but would encourage the authors to consider my comments below.

We thank the reviewer for their time and feedback.

I understand that the following comment is not very useful (because it is not very constructive) but I found the introduction difficult to follow. I think it would be very useful if it is more accessible to a broader readership.

We have edited the introduction, and hope that the clarity is now improved. In particular, we have reorganized the intro to avoid the repeated introduction of biogenesis factors and have substantially rephrased the paragraph in which these proteins are introduced.

Can the clamp in the reconstruction of endogenous PolII-RPAP2-Gdown1 be seen in low-pass filtered maps? I understand that this is not the only human PolII reconstruction that "suffers" from this but I find this very curious. Could it be oscillating or is it disordered? Have the authors tried focused 3D classification? I'm sure they have but it would be useful to comment on this in the paper. I find it remarkable that it is so disordered and that we have almost no signal for it. It is consistent with the recent pre-print from the Cramer lab and reminiscent of the observation by the Roeder lab (Jishage et al., NSMB 2019) – although in the latter case nuclear (and thus assembled) PolII was used as far as I know?!

We were not able to identify any density with features of the Pol II clamp. Focused 3D classification (Relion and cryosparc) and 3D variability analysis (cryosparc) with tight or loose masks encompassing the clamp region revealed only "blobs" of density, similar to what has been seen in a DNA-less class obtained from a Pol II elongation complex data set (Bernecky et al. Nature 2016, Extended Data Fig. 2).

To our knowledge, the clamp domain has not been resolved in any mammalian Pol II cryo-EM reconstruction in the absence of nucleic acids, including the DNA-less Pol II class described above (Bernecky et al. Nature 2016), Pol II dimers (Aibara et al. NAR 2021), Pol II-RPAP2 (Fianu et al. Commun Biol 2021, Wang et al. Cell Reports 2022) and Pol II-Gdown1 (Jishage et al. NSMB 2019). This may be true beyond vertebrates, as an apo-Pol II class derived from Drosophila nuclear extracts also appears to feature a poorly resolved clamp region (Venette-Smith et al. bioRxiv 2025, Fig. 2).

Even in Pol II structures containing nucleic acids, slight variations in clamp conformation can be seen (Bernecky et al. NSMB 2017, Supplementary Fig. 1: "closed, intermediate, and open"). In structures of Pol II bound to inhibitory Alu RNA, the clamp domain is relatively poorly resolved, abeit visible, in structures in which the inhibitory RNA adopts a "canonical" fold. However, the clamp domain is well-resolved in a subset of data containing RNA that is folded into a more EC-like conformation (Tluckova et al. NSMB 2025). It is likely that nucleic acids act to "dock" the clamp domain, and in their absence (or the absence of stabilizing transcription factors) the clamp opens and swivels with a large range of motion, leading to this "invisibility".

We have edited the text as follows to address this point:
"... the Pol II clamp and active site were not resolved, even after extensive 3D classification. Thus, the Pol II clamp domain appears highly flexible, consistent with previous studies of nucleic acid-free Pol II complexes."

Line 222-240: I assume the authors can support the interpretation from the mass-photometry in Fig. 3b with SEC experiments and SDS-PAGE of the peak fractions. If so, it needs to be shown. If feasible, SDS PAGE should show individual peak fractions so we can appreciate if the

composition changes as a function of position along the peaks. In Fig. 3a RPAP2 and GPN1-GPN3 appear sub-stoichiometric – please comment in manuscript?

Extended Data Fig. 4 now includes a size exclusion chromatogram for Pol II–Gdown1–RPAP2–GPN1–GPN3, with SDS-PAGE analysis of the fractions within each peak and mass photometry measurements of a fraction containing the Pol II–Gdown1–RPAP2–GPN1–GPN3 complex. We also replaced the gel image in Fig. 3a – we now display a lane with a higher sample load, so that all proteins are more visible. We comment on the stoichiometry in a new Supplementary Text 2:

"Relative band intensity of RPAP2 and GPN1 proteins after SDS-PAGE analysis and Coomassie (InstantBlue) staining of size exclusion-purified, reconstituted Pol II-Gdown1-RPAP2-GPN1-GPN3 complex suggested that these proteins might be sub-stoichiometrically associated with the complex. To quantify this, we performed mass photometry of the size exclusion-purified, reconstituted Pol II-Gdown1-RPAP2-GPN1-GPN3 complex. This analysis indicated that the majority of the purified complex corresponded to the complete assembly, with a shoulder peak signaling the presence of a small subpopulation of Pol II complexes lacking GPN1-3 and/or RPAP2."

The turquois trace in Fig. 3b shows three (deconvoluted) peaks – is this a mistake or was the mass-photometry data deconvoluted – if so, please state in legend. It would also be useful to label the peaks (e.g. with roman numerals) and write in the figure or legend what each peak likely corresponds to.

Yes- the deconvoluted data are shown, as the figure otherwise becomes quite crowded. We now state in the figure legend that the fitted mass distributions are shown. We have also added labels for the peaks.

The source data image files include mass photometry histograms for three replicates per sample, with each sample shown in a separate graph for legibility. Raw mass photometry counts will be available as source data.

Line 238: Fig. 3b instead of 2b?

We have corrected the figure reference.

Line 238-line 240: The statement that the experiment in Fig. 3b proofs that PolII in complex with RPAP2-GPN1-GPN3 is monomeric in the cytoplasm is only true if we assume that addition of Gdown1-RPAP2-GPN1-GPN3 to mature PolII (presumably purified porcine PolII was the source for this experiment) mimics the cytoplasmic complex prior to import – do we know that? Please comment on that in the text or mention as a potential caveat and/or limitation.

We do believe that porcine Pol II purified from whole cell extract mimicks Pol II purified from the cytoplasm, based on several observations:

1. The structure of the resolved regions of Pol II indistinguishable from that of previously solved cryo-EM structures obtained with Pol II purified from whole cell lysate (Tluckova et al. NSMB 2025). Excluding the clamp domain, the structure is also extremely similar to Pol II purified from nuclear extracts, with only only small conformational changes, likely due to the presence of nucleic acid (Kujirai et al. Nat Comm 2025). However, based on these analyses, we cannot

exclude that there are differences in post-translational modifications, especially within the C-terminal domain of RPB1 which is not structurally resolved.

2. The structure of the Pol II-RPAP2 complex prepared from isolated porcine Pol II and recombinant RPAP2 (Fianu et al. Comm Biol 2021) is essentially the same as what was observed within the cytoplasmic complex (this manuscript and Schmitzová et al. bioRxiv 2025).

3. We can reconstitute a complex containing Gdown1, RPAP2, and GPN1-3 using porcine Pol II purified from whole cell extracts. Additionally, we can purify a complex enriched in Gdown1, RPAP2, and GPN1-3 from nuclear extracts, when Gdown1 affinity is used. Thus, is is likely that the main difference in cytoplasmic Pol II just before import and Pol II in the nucleus is the complement of bound factors.

Still, to remain conservative, we have changed the phrasing to say "strongly suggest" rather than "indicate", and the text now reads:

"Taken together, these observations strongly suggest that cytoplasmic Pol II exists predominantly in a monomeric form in proliferating cells, consistent with the lack of observed dimers in cryo-EM of the native, cytoplasmic Pol II."

Line 253-269: AF3 presumably predicts PolII with an ordered and closed clamp domain – the authors should comment on this since they then argue that an open clamp is likely required for binding and they do the biochemical experiments described in lines 261-269 to support this claim.

The Pol II-GPN1-3 AphaFold prediction in the original version of the manuscript included a version of Pol II lacking the clamp domain, because of structural clashes between Pol II and GPN1-GPN3 when the clamp domain was included. We realize that this was not adequately described in the submitted manuscript, and have now rephrased this in the main text, methods, and Extended Data Fig. 4.

Line 242-269: Along the same lines, it would be important to state more clearly what the experimental proof is for assuming GPN1-GPN3 on its own binds the PolII cleft (aside from an AF3 prediction). The crosslinking mass spectrometry (Extended Fig. 4f) shows cross-links to the N-terminus and the disordered CTD - not necessarily the clamp, correct?

Our main evidence that GPN1-GPN3 binds to the Pol II cleft is the experiment in Fig. 3e, showing that the addition of elongation complex nucleic acids (which would fill and close the DNA-binding cleft) abolishes the binding of GPN1-GPN3 to Pol II. If GPN1-GPN3 primarily bound through the RPB4/7 stalk or CTD as previously described, we would have expected nucleic acids to have no effect. We have edited the text as follows to make this clearer:

"The predicted GPN1-GPN3 and Pol II interaction would be blocked in a Pol II elongation complex, through steric clashes with bound nucleic acid and clamp closure. To test this, we incubated GPN1–GPN3 with apo or nucleic acid-bound forms of Pol II in a pulldown binding assay (Fig. 3e)."

The complete clamp domain of Pol II includes RBP1 1-360, RBP1 1414-1466, and RBP2 1062-1174. In our XL-MS data, one of the crosslinks between GPN1 and RPB1 is indeed to the very N-terminus of RPB1, a region of the clamp domain not resolved in the structure of elongation

complexes. The second crosslink of GPN1 to to RBP1 (K42) falls within a well-ordered region of the clamp.

In the recent cytoplasmic Pol II structure preprint from the Cramer lab, Schmitzová et al. performed XL-MS of purified cytoplasmic Pol II complexes. The authors observed three crosslinks between GPN3 and RBP2, in the region of the DNA-binding cleft. When mapped to our AF3 prediction of Pol II (without clamp)-GPN1-GPN3, these crosslinks occur between residues with Cα-Cα distances <20 Å, further supporting a localization of GPN1-GPN3 to the DNA binding cleft, at least within the context of the full complex. We have added a section within the discussion to point this out.

Also, why was the AF3 prediction done with PolII and GPN1-GPN3, when the mass spectrometry was done with PolII-Gdown1-RPAP2-GPN1-GPN3? What does the AF3 prediction look like for the whole complex?

The AF3 prediction was carried out for Pol II, GPN1, and GPN3 to investigate the direct interaction between GPN1-GPN3 and Pol II. Because of protein size limitations with the AlphaFold3 server, it was not possible to predict the full complex with all subunits of Pol II included. In Revision Fig. 3 below, we show the AF3 prediction for the whole complex, but in which the Pol II clamp and RPB4/7 stalk are excluded. The interaction between Pol II and GPN1-GPN3 is essentially unchanged.

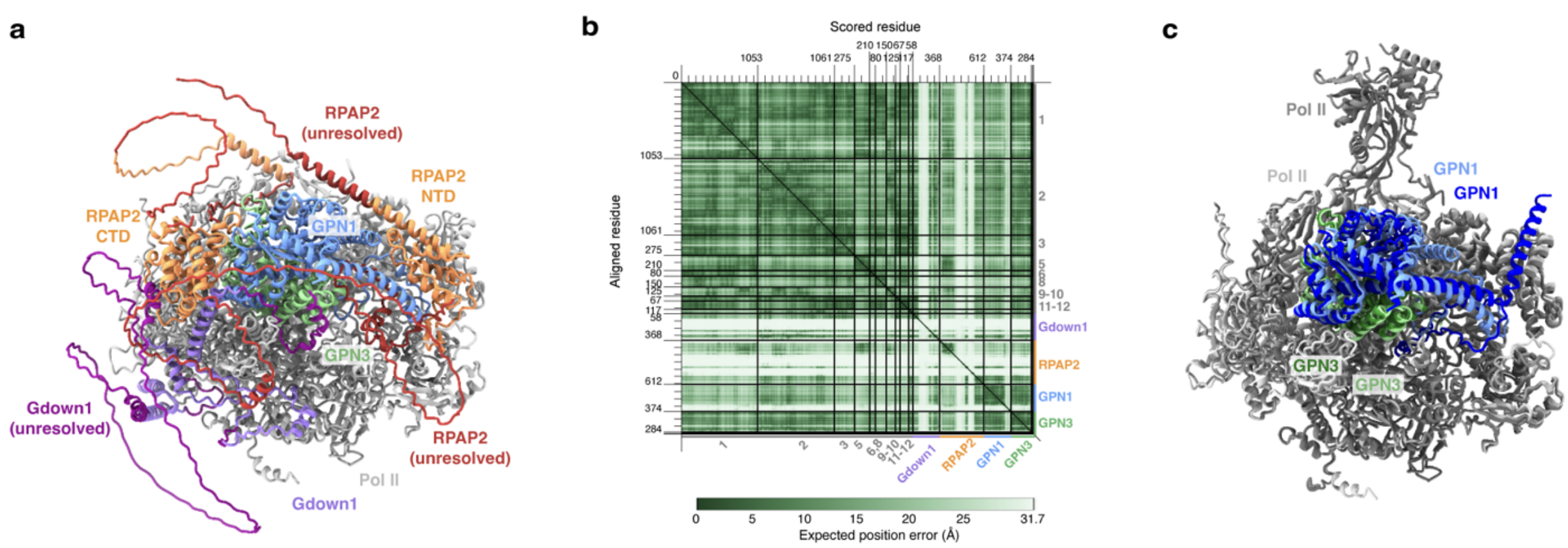

Revision Figure 3*. Comparison of Pol II-Gdown1-RPAP2-GPN1-GPN3 and Pol II-GPN1-GPN3 predicted structures.* ***a****, AlphaFold3 prediction of Pol II-Gdown1-RPAP2-GPN1-GPN3 without the Pol II clamp and stalk. Regions of RPAP2 and Gdown1 not resolved in our cytoplasmic Pol II structure are colored red-brown and dark purple, respectively.* ***b****, PAE plot of structure prediction shown in (a). PTM, 0.84; iPTM, 0.82.* ***c****, Overlay of structure predictions of Pol II (no clamp, no stalk)-Gdown1-RPAP2-GPN1-GPN3 and Pol II (no clamp)-GPN1-GPN3, after alignment of the RPB2 subunits of each, with only Pol II, GPN1, and GPN3 shown. Pol II (no clamp, no stalk)-Gdown1-RPAP2-GPN1-GPN3 is shown in lighter colors (Pol II, light gray; GPN1, light blue, GPN3, light green), and Pol II (no clamp)-GPN1-GPN3 shown in darker colors (Pol II, gray; GPN1, blue; GPN3, green).*

Extended Fig. 4f: Are the crosslinks between GPN1-GPN3 and RPB1 on the one hand and with RPAP2 on the other hand compatible? In other words, does the crosslinking data support two alternative binding modes of GPN1-GPN3 (to the cleft and to the RPAP2 CTD) or could crosslinks to the clamp and RPAP2 be satisfied at the same time?

The crosslinks appear compatible, although they generally occur in flexible regions of one or both of the proteins involved, limiting their usefulness as structural restraints. Like GPN1, the RPAP2 C-terminus crosslinks to RBP1 residues within the clamp domain.

Pol II binds RPAP2 through its structured N-terminal domain, which is followed by a disordered linker, and finally the RPAP2 C-terminal domain which links to GPN1-GPN3. The alpha-helical, GPN1-binding domain of RPAP2 would face away from the Pol II binding cleft, in proximity to the disordered C-terminus of Gdown1, which does crosslink to the RPAP2 C-terminal domain. GPN1-GPN3 would remain in close proximity to the RPB1 clamp domain.

To summarize, simultaneous binding of GPN1-GPN3 to the cleft and to the RPAP2 CTD appears possible, but GPN1-GPN3 localization to the cleft may be transient in the context of the Pol II-Gdown1-RPAP2-GPN1-GPN3 complex.

We have added an additional Supplementary figure 6, referenced in the discussion section, to visualize a putative Pol II-Gdown1-RPAP2-GPN1-GPN3 complex with GPN1-GPN3 docked in the DNA-binding cleft. We generated this illustrative model by merging the RPAP2-GPN1-GPN3 structure into the Pol II-Gdown1-RPAP2 structure in the position predicted in the Pol II (no clamp)-GPN1-GPN3 AF3 prediction. We have mapped our GPN1, GPN3, and RPAP2 interlinks, as well as the three crosslinks between GPN3 and RPB2 reported in Schmitzová et al. bioRxiv 2025.

Paragraph starting at line 271 and Fig. 3: The authors have presumably aimed to obtain a cryo-EM reconstruction of the PolII-Gdown1-RPAP2-GPN1-GPN3 and/or PolII-RPAP2-GPN1-GPN3 complex using the recombinant proteins and porcine PolII, correct? Please comment on why this may have failed or, alternatively, why there was no attempt to do that? Do we know if binding of all or a subset of these factors can "reverse" PolII assembly (see also one of the previous comments)? Have they considered adding recombinant RPAP2, GPN1-GPN3 to the endogenous cytoplasmic PolII complex similar to what had been described in the Cramer lab pre-print? If not, why not – please comment on that in the manuscript?

As the reviewer suspected, we did also perform cryo-EM of the reconstituted Pol II-Gdown1-RPAP2-GPN1-GPN3 complex. We prepared cryo-EM grids of the size-exclusion purified complex, and used cryosparc for the initial analysis. We could obtain a reconstruction at a nominal resolution of 3.3 Å, but the reconstruction looked essentially the same as what we obtained after analysis of the endogenous cytoplasmic complex (Revision Figure 4 below), with no hint of density for GPN1-GPN3 visible. This led us to believe that flexibility rather than stoichiometry of the complex was the main limiting factor. We therefore decided to focus our efforts on elucidating the structure of the GPN1-GPN3-RPAP2 complex, rather than pursuing this analysis further.

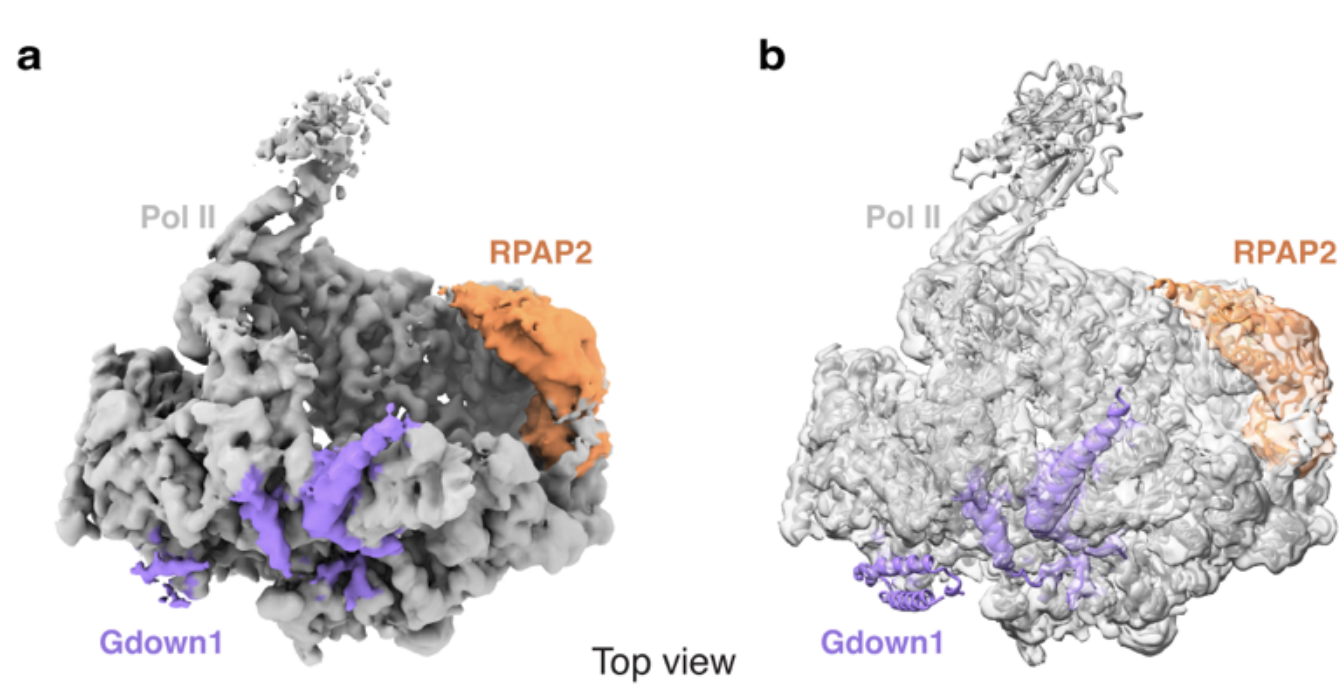

Revision Figure 4*. Cryo-EM analysis of the in vitro reconstituted Pol II-Gdown1-RPAP2-GPN1-GPN3 complex.* ***a****, Cryo-EM reconstruction of the in vitro reconstituted Pol II-Gdown1-RPAP2-GPN1-GPN3 complex, unsharpened map. Pol II, gray; RPAP2, orange, Gdown1, purple.* ***b****, The cytoplasmic Pol II-Gdown1-RPAP2 structural model with the density in (a) overlaid.*

We do not believe it is likely that any of these specific factors (i.e. RPAP2, GPN1, GPN3) can reverse Pol II assembly, particularly since we have not observed the formation of any smaller

Pol II subcomplexes in mass photometry or size exclusion experiments, and because these factors are all present and can associate with Pol II in the nucleus after import.

As the supplemented cytoplasmic Pol II structure in Schmitzová et al. appears essentially identical to the structure we resolved, we can be confident that stoichiometry was not the reason that GPN1-GPN3 were not resolved in our cryo-EM analysis. We have now added the following text discussion section:
"Furthermore, their [Schmitzová et al.'s] work included cryo-EM analysis of a cytoplasmic Pol II complex supplemented with recombinant Gdown1 and RPAP2-GPN1-GPN3 complex to improve stoichiometry. The structure reported in Schmitzová et al. is highly similar to the structure of cytoplasmic Pol II reported here, suggesting that flexibility of bound GPN1-GPN3, rather than substoichiometry in the endogenous complex, prevents visualization of GPN1-GPN3 in the cytoplasmic Pol II complex."

Line 301-325 and Fig.5: Why is the peak for the complex higher in presence of GDPNP compared to GTP – is there some binding issue of the analogue to the complex and we see some apo form? Also, apo and GDP are identical – are we sure GDP binds in the experimental conditions used here?

We do see a consistently higher peak for the RPAP2-bound complex in the presence of GDPNP. We believe that this may be because GTP hydrolysis may more efficiently prevent RPAP2-GPN1/3 complex assembly, and we speculate on this in the discussion.

We did not measure apo GPN1-GPN3 in this experiment, but rather GDP-bound GPN1-GPN3, as it is purified from insect cells. Thus, the experiment should indeed look the same, as long as the presence of 10 mM nucleotide per se does not change RPAP2-GPN1/3 complex stability. We have clarified this in the text.

Line 335: Please be more specific – what about Gdown1 is revised compared to previous work?

We have revised the text near lines 142 and 335 of the original submission. These sections now read:
"However, due to improved density, a previously existing model for Pol II-Gdown1 could be revised. In the updated model, the N-terminal Pol II-binding regions of Gdown1 were revealed to be discontinuous, and the sequence register of both regions of Gdown1 could be assigned. Furthermore, the model for the Gdown1 region binding to the RPB2 protrusion could be extended"
and
"In the revised Gdown1 structure presented here, three distinct Pol II-binding regions could be discerned and their sequence register assigned. These updates allowed clarification of the interactions responsible for the transcription inhibition activity of Gdown1"

Line 445: the reference is missing

The missing reference has been added.

Line 570: Can you see particles at a defocus of -0.3? How reliable is picking at such a low defocus value?

If the micrograph is denoised, the particles are just barely visible at this defocus. The number of picked particles (cryoSPARC blob picker) is reduced from an average of 450 particles per micrograph to an average of 40.

Although we did not actively exclude the low defocus micrographs, the low defocus particles made up a very small percentage of the final data set. Only 0.15% of the particles originated from micrographs with a defocus of -0.5 µm or closer to focus, and only two micrographs with a defocus of -0.3 µm were included.

List of responses to reviewer comments

"Structure of cytoplasmic RNA polymerase II"
(Annamaria Hlavata, Benjamin Neuditschko, Ulla Schellhaas, Clemens Plaschka, Franz Herzog, and Carrie Bernecky)

Nature Communications manuscript NCOMMS-25-94449-T

Responses are in dark blue *italics*. Text changes are indicated in red.

REVIEWERS' COMMENTS

Reviewer #1 (Remarks to the Author):

The authors have addressed our comments and concerns. We recommend publication of the manuscript in Nature Communications.

That said, we would encourage the authors to try to find alternative wording for "... reveal a molecular switch governing biogenesis factor association..." in the abstract to more accurately reflect the experimental findings reported in the paper. However, in order to avoid undue delays to the publication process, this should be agreed between the authors and the editor and will not require further external review.

We have slightly edited the abstract as suggested by the reviewer, to be as accurate as possible. The related sections of the abstract now read:

"Further biochemical and cryo-EM analysis reveals how RPAP2 tethers GPN1–GPN3 to the complex" and "...reveal a molecular switch regulating biogenesis factor association...".

We thank the reviewer (and all other reviewers) for their time and help in improving the manuscript.

Reviewer #2 (Remarks to the Author):

I co-reviewed this manuscript with one of the reviewers who provided the listed reports. This is part of the Nature Communications initiative to facilitate training in peer review and to provide appropriate recognition for Early Career Researchers who co-review manuscripts.

Reviewer #3 (Remarks to the Author):

The authors have clarified the few issues/remarks and the manuscript can be accepted in its current form.

Reviewer #4 (Remarks to the Author):

Thank for sending the revised manuscript. The authors have addressed all my concerns and I support publication.
