# Supplementary material for: Structure of cytoplasmic RNA polymerase II
Source: Nat Commun. 2026 Jul 13;17:8640. doi: 10.1038/s41467-026-75416-8 (PMC13486763; doi:10.1038/s41467-026-75416-8)
Supplement: Supplementary file 4 [file 41467_2026_75416_MOESM4_ESM.zip › Source_data_figure_3/Source_data_figure_3.pdf]

a

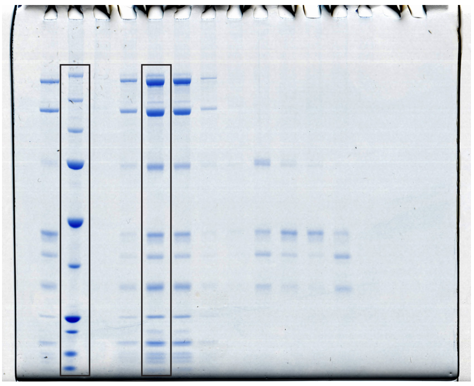

b

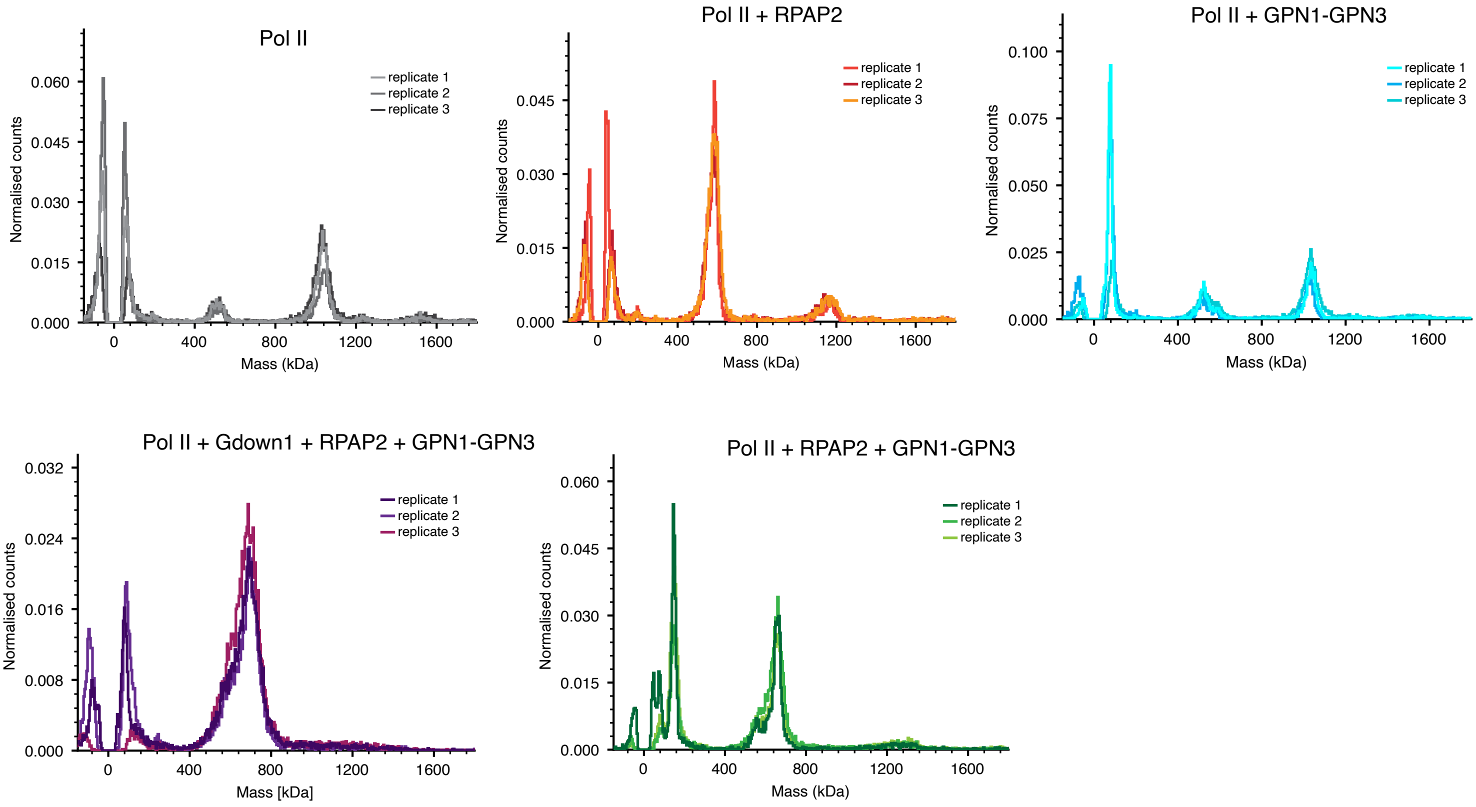
Pol II
replicate 1
replicate 2
replicate 3
Normalised counts
0.060
0.045
0.030
0.015
0.000
0
400
800
1200
1600
Mass (kDa)
Pol II + RPAP2
replicate 1
replicate 2
replicate 3
Normalised counts
0.045
0.030
0.015
0.000
0
400
800
1200
1600
Mass (kDa)
Pol II + GPN1-GPN3
replicate 1
replicate 2
replicate 3
Normalised counts
0.100
0.075
0.050
0.025
0.000
0
400
800
1200
1600
Mass (kDa)
Pol II + Gdown1 + RPAP2 + GPN1-GPN3
replicate 1
replicate 2
replicate 3
Normalised counts
0.032
0.024
0.016
0.008
0.000
0
400
800
1200
1600
Mass [kDa]
Pol II + RPAP2 + GPN1-GPN3
replicate 1
replicate 2
replicate 3
Normalised counts
0.060
0.045
0.030
0.015
0.000
0
400
800
1200
1600
Mass (kDa)

e

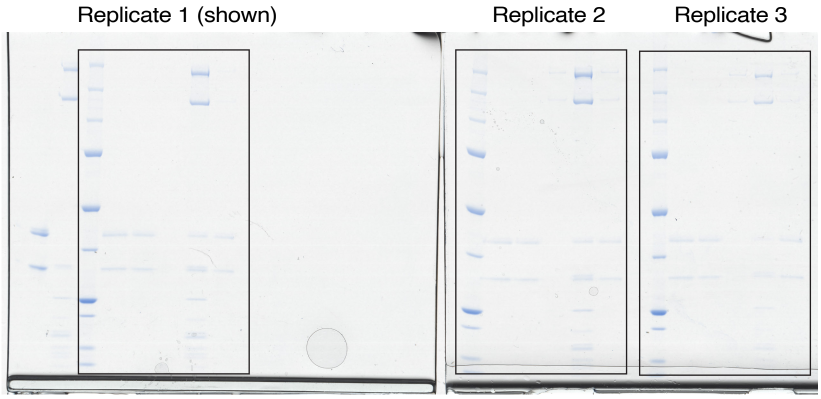
Replicate 1 (shown)
Replicate 2
Replicate 3
