# Supplementary material for: Structure of cytoplasmic RNA polymerase II
Source: Nat Commun. 2026 Jul 13;17:8640. doi: 10.1038/s41467-026-75416-8 (PMC13486763; doi:10.1038/s41467-026-75416-8)

a

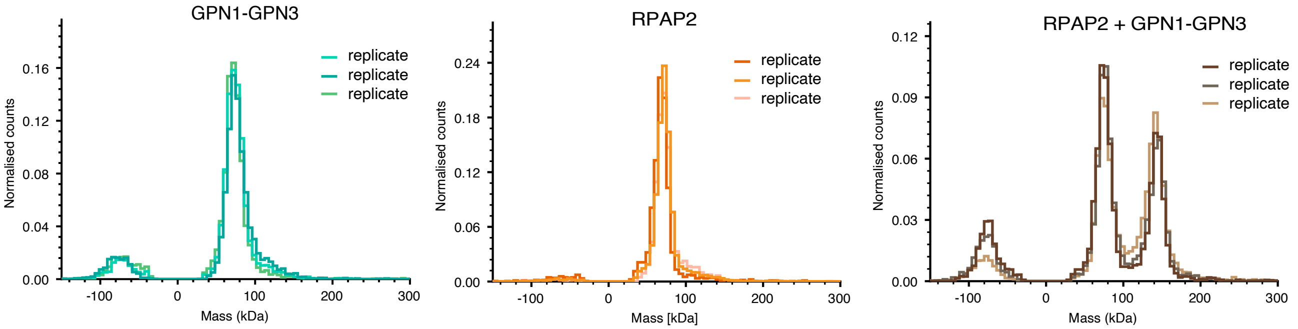
GPN1-GPN3
replicate
replicate
replicate
Normalised counts
0.16
0.12
0.08
0.04
0.00
-100
0
100
200
300
Mass (kDa)
RPAP2
replicate
replicate
replicate
0.24
0.18
0.12
0.06
0.00
Mass [kDa]
RPAP2 + GPN1-GPN3
replicate
replicate
replicate
0.12
0.09
0.06
0.03
0.00

b

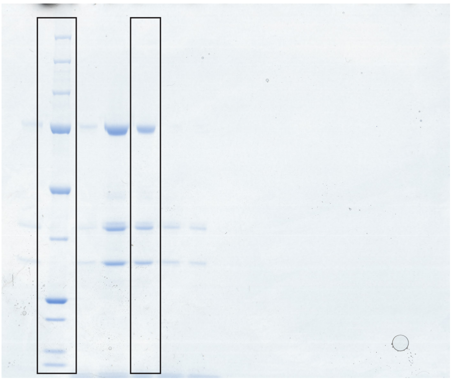

Supplement: Supplementary file 4 [file 41467_2026_75416_MOESM4_ESM.zip › Source_data_figure_4/Source_data_figure_4.pdf]
