# Supplementary material for: Structure of cytoplasmic RNA polymerase II
Source: Nat Commun. 2026 Jul 13;17:8640. doi: 10.1038/s41467-026-75416-8 (PMC13486763; doi:10.1038/s41467-026-75416-8)
Supplement: Supplementary file 4 [file 41467_2026_75416_MOESM4_ESM.zip › Source_data_figure_5/Source_data_figure_5.pdf]

d

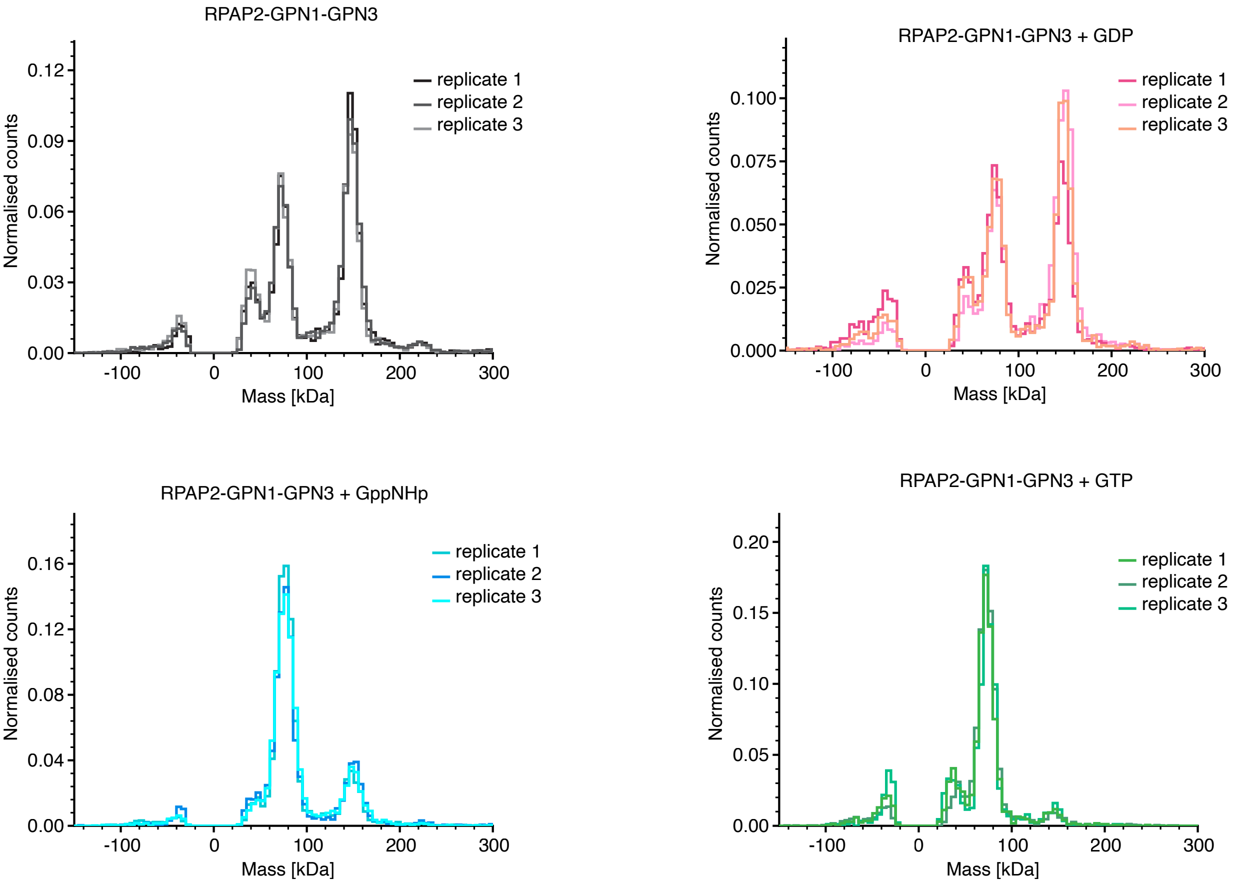
RPAP2-GPN1-GPN3
Normalised counts
0.12
0.09
0.06
0.03
0.00
-100
0
100
200
300
Mass [kDa]
replicate 1
replicate 2
replicate 3
RPAP2-GPN1-GPN3 + GDP
Normalised counts
0.100
0.075
0.050
0.025
0.000
-100
0
100
200
300
Mass [kDa]
replicate 1
replicate 2
replicate 3
RPAP2-GPN1-GPN3 + GppNHp
Normalised counts
0.16
0.12
0.08
0.04
0.00
-100
0
100
200
300
Mass [kDa]
replicate 1
replicate 2
replicate 3
RPAP2-GPN1-GPN3 + GTP
Normalised counts
0.20
0.15
0.10
0.05
0.00
-100
0
100
200
300
Mass [kDa]
replicate 1
replicate 2
replicate 3
