# Supplementary material for: Structure of cytoplasmic RNA polymerase II
Source: Nat Commun. 2026 Jul 13;17:8640. doi: 10.1038/s41467-026-75416-8 (PMC13486763; doi:10.1038/s41467-026-75416-8)
Supplement: Supplementary file 4 [file 41467_2026_75416_MOESM4_ESM.zip › Source_data_supplementary_figure_1.pdf]

c

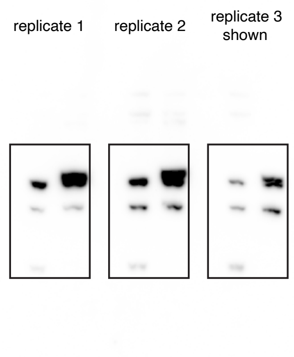
replicate 1
replicate 2
replicate 3
shown

d

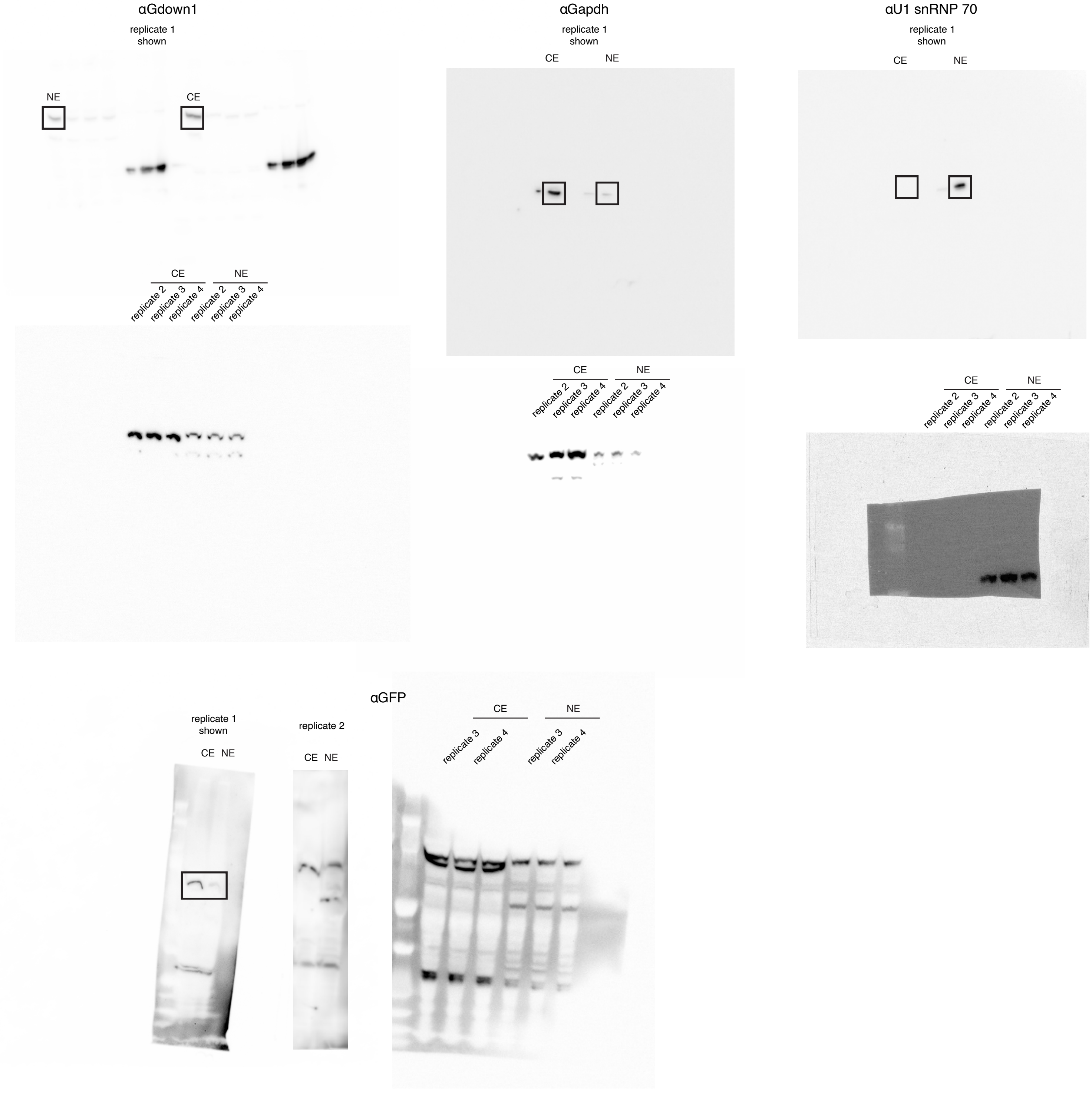
αGdown1
replicate 1
shown
NE
CE
CE
NE
replicate 2
replicate 3
replicate 4
replicate 2
replicate 3
replicate 4
αGapdh
replicate 1
shown
CE
NE
CE
NE
replicate 2
replicate 3
replicate 4
replicate 2
replicate 3
replicate 4
αU1 snRNP 70
replicate 1
shown
CE
NE
CE
NE
replicate 2
replicate 3
replicate 4
replicate 2
replicate 3
replicate 4
αGFP
replicate 1
shown
CE
NE
replicate 2
CE
NE
CE
NE
replicate 3
replicate 4
replicate 3
replicate 4
