# Supplementary material for: Structure of cytoplasmic RNA polymerase II
Source: Nat Commun. 2026 Jul 13;17:8640. doi: 10.1038/s41467-026-75416-8 (PMC13486763; doi:10.1038/s41467-026-75416-8)

a

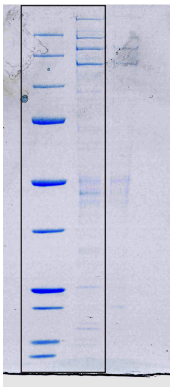

b

Pol II

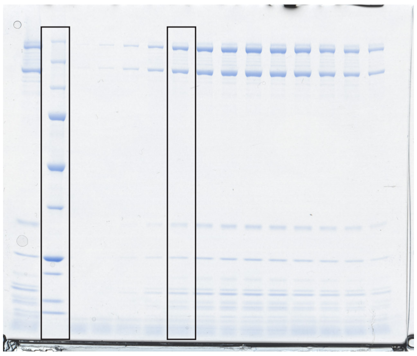

RPAP2

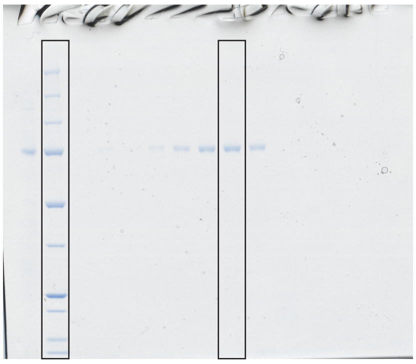

GPN1-GPN3

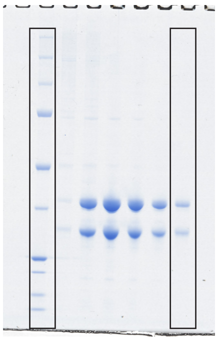

Gdown1

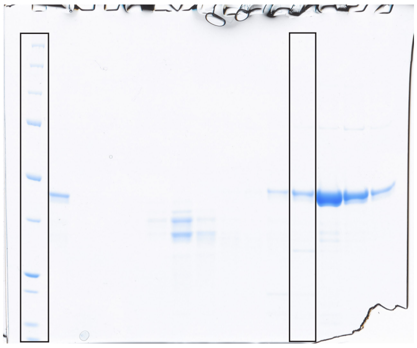

d

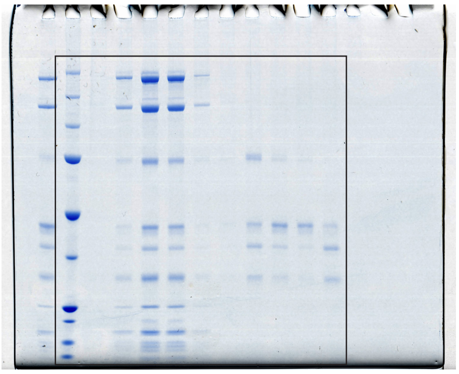

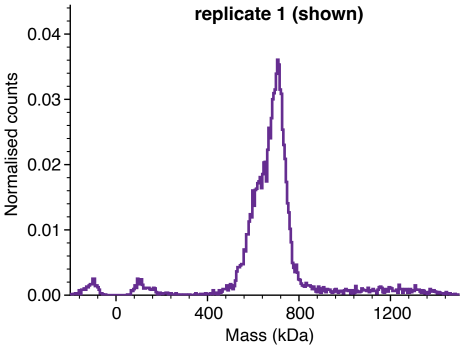

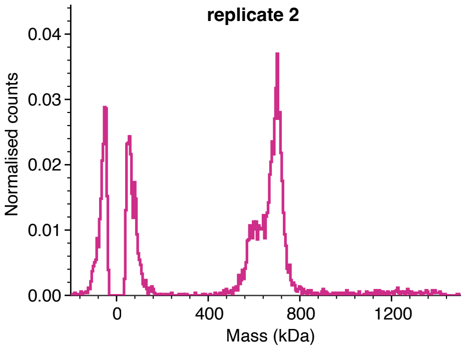

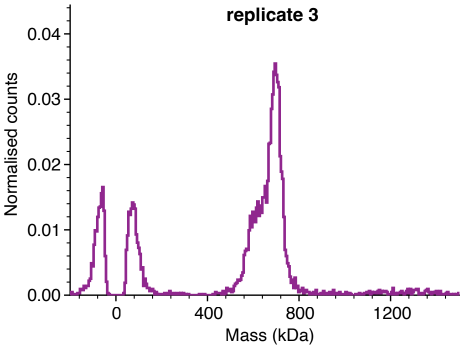

Supplement: Supplementary file 4 [file 41467_2026_75416_MOESM4_ESM.zip › Source_data_supplementary_figure_4/Source_data_supplementary_figure_4.pdf]
