# Supplementary material for: Structure of cytoplasmic RNA polymerase II
Source: Nat Commun. 2026 Jul 13;17:8640. doi: 10.1038/s41467-026-75416-8 (PMC13486763; doi:10.1038/s41467-026-75416-8)
Supplement: Supplementary file 4 [file 41467_2026_75416_MOESM4_ESM.zip › Source_data_supplementary_figure_5.pdf]

a

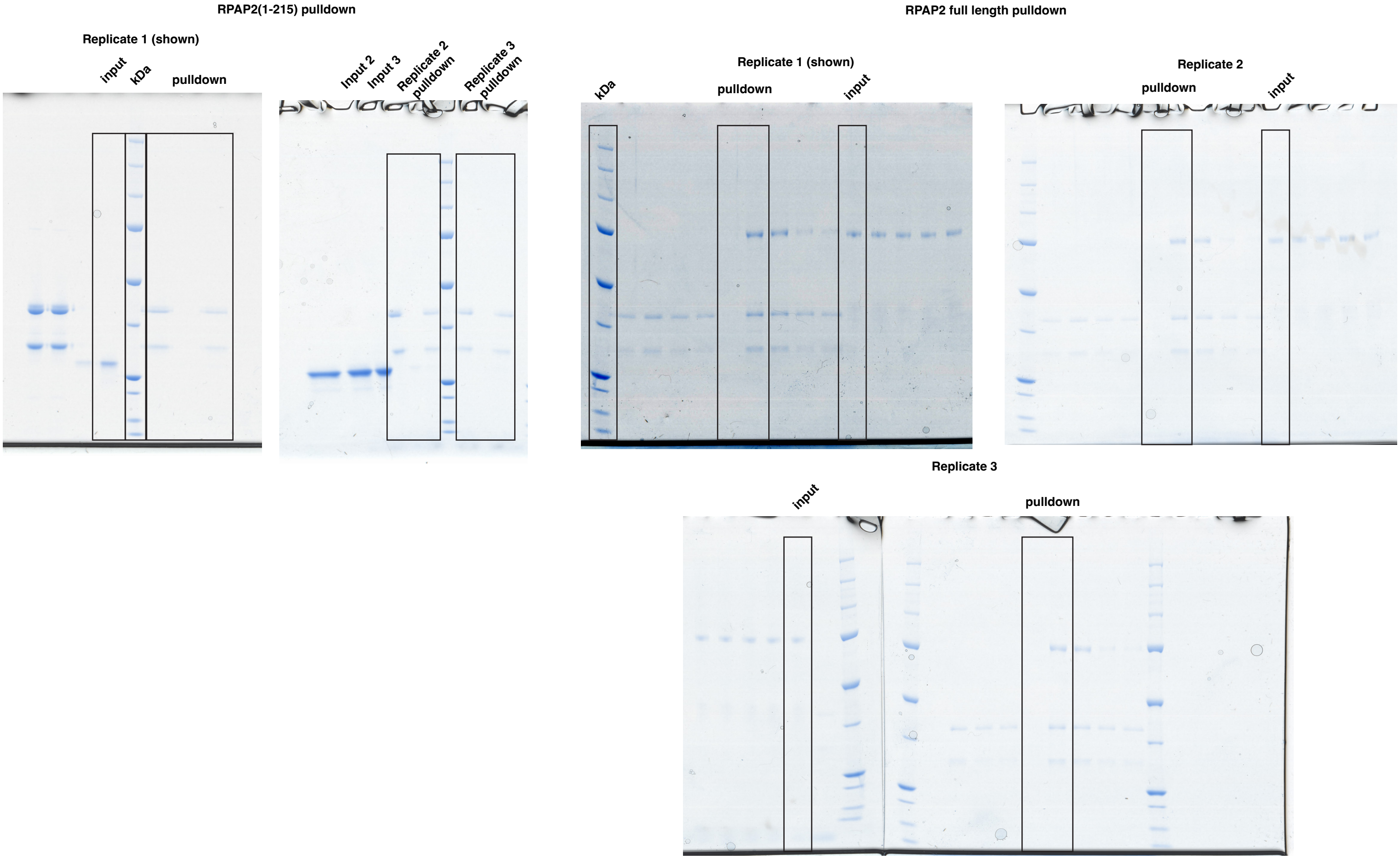
RPAP2(1-215) pulldown
Replicate 1 (shown)
input
kDa
pulldown
Input 2
Input 3
Replicate 2 pulldown
Replicate 3 pulldown
RPAP2 full length pulldown
Replicate 1 (shown)
kDa
pulldown
input
Replicate 2
pulldown
input
Replicate 3
input
pulldown

q

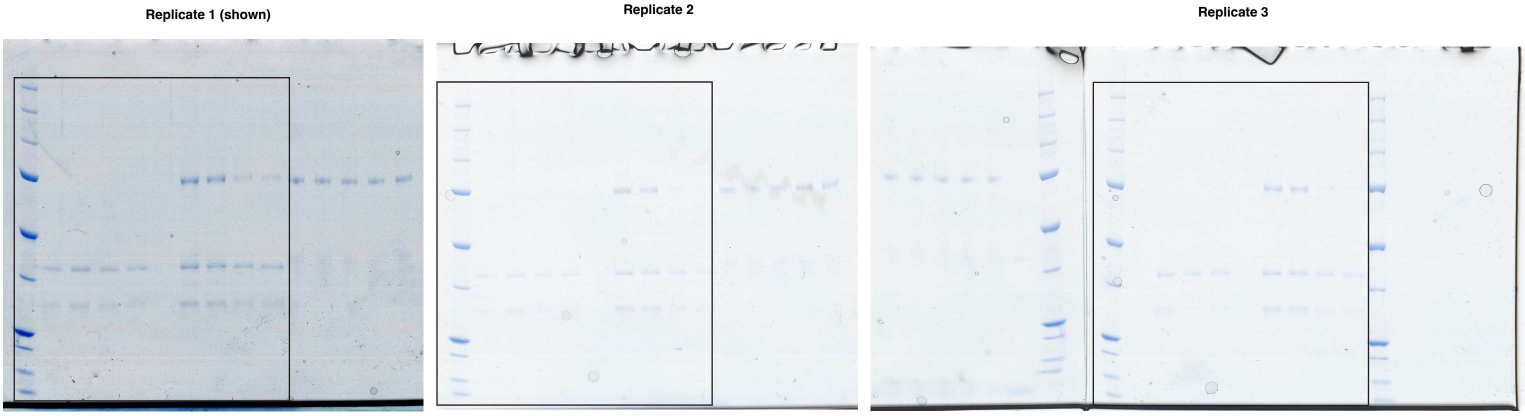
Replicate 1 (shown)
Replicate 2
Replicate 3
